# ScillyHAB: A Multi-Disciplinary Survey of Harmful Marine Phytoplankton and Shellfish Toxins in the Isles of Scilly: Combining Citizen Science with State-of-the-Art Monitoring in an Isolated UK Island Territory

**DOI:** 10.3390/md23120478

**Published:** 2025-12-15

**Authors:** Andrew D. Turner, Karl J. Dean, Adam M. Lewis, David M. Hartnell, Zoe Jenkins, Beth Bear, Amy Mace, Nevena Almeida, Rob van Ree, Kerra Etchells, Issy Tibbs, Patrick Jesenko, Loveday Lewin, Natalie Robey, Nikki Banfield, Shamina Page, George Belsham, Benjamin H. Maskrey, Robert G. Hatfield

**Affiliations:** 1 Centre for Environment Fisheries and Aquaculture Science (Cefas), Barrack Road, Weymouth, Dorset DT4 8UB, UK; karl.dean@cefas.gov.uk (K.J.D.); adam.lewis@cefas.gov.uk (A.M.L.); david.hartnell@cefas.gov.uk (D.M.H.); shamina.page@cefas.gov.uk (S.P.); george.belsham@cefas.gov.uk (G.B.); robert.hatfield@cefas.gov.uk (R.G.H.); 2 ScillyHAB Field Office, Normandy House, St. Mary’s, Isles of Scilly TR21 0NY, UK; zoe.ellen1999@gmail.com (Z.J.);; 3 Cefas, Pakefield Road, Lowestoft NR33 0HT, UKamy.mace@cefas.gov.uk (A.M.); r.vanree@apemltd.co.uk (R.v.R.); 4 Five Islands Academy, Church Road, St. Mary’s, Isles of Scilly TR21 0NA, UK; 5 School of Life Sciences, University of Sussex, Falmer, Brighton BN1 9QG, UK; l.lewin@sussex.ac.uk; 6 Isles of Scilly Wildlife Trust, Trenoweth, Isles of Scilly TR21 0NS, UK

**Keywords:** Harmful Algal Blooms (HABs), shellfish toxins, Isles of Scilly, citizen science, Tetrodotoxin (TTX), nanopore sequencing

## Abstract

The Isles of Scilly are an archipelago of islands in the far southwest of the UK which contain numerous beds of wild bivalve molluscs which are recreationally harvested for local consumption. However, the islands have never previously been assessed for the presence of harmful algae and their shellfish toxin metabolites which can cause serious human health impacts. This study sought to address these knowledge gaps through the analysis of seawater and shellfish tissues for microalgae and toxins utilizing portable and lab-based microscopy, nanopore sequencing, chemical analysis and immunoassay kits. The study design was affected by the national COVID-19 lockdown which enforced implementation of citizen-led sampling and in-field microscopy. Microscopy and sequencing approaches led to the confirmation of multiple HAB species of concern, including those potentially responsible for production of neurotoxic and diarrhetic shellfish toxins. A portable microscope was successfully utilized in the field for recognition of microalgae and for early warning of potential shellfish toxicity events. Chemical analysis of cockle, clam and mussel samples confirmed the detection of paralytic, diarrhetic and amnesic shellfish toxins, with an unusual okadaic acid group toxin profile reaching a maximum toxicity of approximately half the regulatory limit as defined by EU law. The Sensoreal Alert Lateral Flow Assay was used to screen and highlight samples containing higher concentrations of DSP toxins. Furthermore, Tetrodotoxin was detected for the first time in the UK in cockle and grooved carpet shells. Multiple saxitoxin analogues were also detected in two echinoderm species, with this providing the first ever report of paralytic shellfish toxins in the spiny starfish, *Marthasterias glacialis*. The toxin profiles in the two species varied significantly with a dominance of GTX4 in *Luidia ciliaris* as opposed to a dominance of STX in *Marthasterias glacialis.* Overall, the study showed that a multi-method assessment of a previously unexplored region within the UK territory contained microalgae and toxins of concern to human health, and that a citizen-led programme could be instigated using portable microscopy and rapid toxin testing to assess the early warning for potentially harmful microalgae and toxins in the region, with confirmatory analysis being conducted to establish actual levels of risk for local consumers of seafood.

## 1. Introduction

Marine Harmful Algal Blooms (HABs) result from the proliferation of certain algal species which can impact upon aquatic ‘One Health’, including the health of aquatic animals, humans and even entire ecosystems [1,2,3,4,5,6]. Many different factors are known to cause such blooms including changes in water temperature, salinity, stratification, weather events and nutrient inputs [7]. HABs can impact health either directly, through oxygen-depletion or sweater, or from the direct exposure of algal cells or their metabolites to other organisms such as fin fish, or indirectly, with the transfer of phycotoxins from contaminated organisms to higher trophic levels [8,9,10]. The majority of HAB species are from the Dinoflagellate phylum, although certain diatom, raphidophyte and haptophyte species are also important organisms in relation to both animal and ecosystem health as well as some diatoms such as *Pseudo-nitzchia* affecting human health [2,11,12]. Toxins produced by many of the HAB species are known to affect human health, with a wide range of poisoning syndromes recognized following consumption of contaminated seafood. Most notably, bivalve mollusc shellfish can accumulate these toxins through filter feeding on microalgae, with health impacts varying depending on the biological mode of action of the toxins ingested [4,13,14] as well as the concentration of toxins in the shellfish flesh [15].

Given the prevalence of multiple phycotoxin-producing HAB species around the world and the inherent risks to the health of seafood consumers, there is a legislative requirement in most regions of the world to conduct official control testing of shellfisheries for both phytoplankton identification of potentially toxic taxa and cell density enumeration in water samples as well as the analysis of shellfish flesh for regulated biotoxins [16,17,18,19,20]. In the European Union (EU) as well as the United Kingdom (UK), testing must be conducted routinely for phytoplankton and toxins associated with Paralytic Shellfish Poisoning (PSP), Amnesic Shellfish Poisoning (ASP), Diarrhetic Shellfish Poisoning (DSP) and Azaspiracid Shellfish Poisoning (AZP) as well as for yessotoxins (YTXs) [16,17,18,19,20]. PSP is the primary cause of marine toxin-related mortality in seafood consumers globally [4,21,22], with the causative toxins, saxitoxins, originating from four HAB genera; *Alexandrium, Centrodinium, Gymnodinium* and *Pyrodinium* [23,24,25] and found across hugely variable environmental conditions [14,26]. ASP, caused by domoic acid and related isomers, produced by diatoms of the genus *Pseudo-nitzschia,* also occurs globally but to date has only been associated with relatively few incidents of human intoxication, most notably in Canada during the 1980s with health impacts including gastrointestinal issues and long-term neurological disorders including memory defects [27,28,29,30,31,32,33]. A more recent event with lower impact was reported in Spain, when two human poisoning cases were recorded in a family who recreationally harvested mussels from a classified shellfish growing area which was already closed to harvesting [34]. DSP, the most commonly occurring shellfish toxin poisoning syndrome in UK waters is also found globally and is associated with the presence of Okadaic acid (OA) and the related dinophysistoxins (DTXs) produced by dinoflagellate genera such as *Dinophysis* and benthic *Prorocentrum* species [35,36,37,38,39,40,41]. In the UK, the last known human poisoning from PSP was in the late 1960s, whereas DSP outbreaks occur to this day, noting even so that incidents can be difficult to confirm given their purely gastrointestinal symptoms [42]. In addition to the EU-regulated toxins, other classes of shellfish toxins have been reported throughout the world including the dinoflagellate-produced Brevetoxins (BTXs) [43,44,45], Palytoxins (PlTxs) and Cyclic imines (CIs) [46,47,48], as well as Tetrodotoxins (TTXs) which are evidenced to be produced by marine bacteria [49,50,51,52,53]. To date, both CIs and TTXs have been detected in UK shellfish as well as in neighbouring countries [48,51,54,55,56].

As well as being a regulatory requirement, shellfish and water testing is essential for managing the risks from HABs and phycotoxins given that prediction and mitigation strategies are in their infancy [6,57]. Currently official control testing relies upon the application of optical microscopy for phytoplankton analysis and chemical detection methods for shellfish toxins, specifically liquid chromatography with ultraviolet detection (LC-UV) for ASP [58,59], LC with fluorescence detection (LC-FLD) for PSP analysis [19,60,61] and LC with tandem mass spectrometry (LC-MS/MS) for lipophilic toxins (LT), incorporating DSP, AZA and YTX toxins [16,17,18,62,63,64]. Other methods are available, however, for research-based assessment of HABs and their related phycotoxins, including the recent development of molecular biological and next generation sequencing tools for phytoplankton detection [65,66] and more specific and sensitive mass spectrometric methods for PSP toxins [67,68,69].

**Figure 1:**
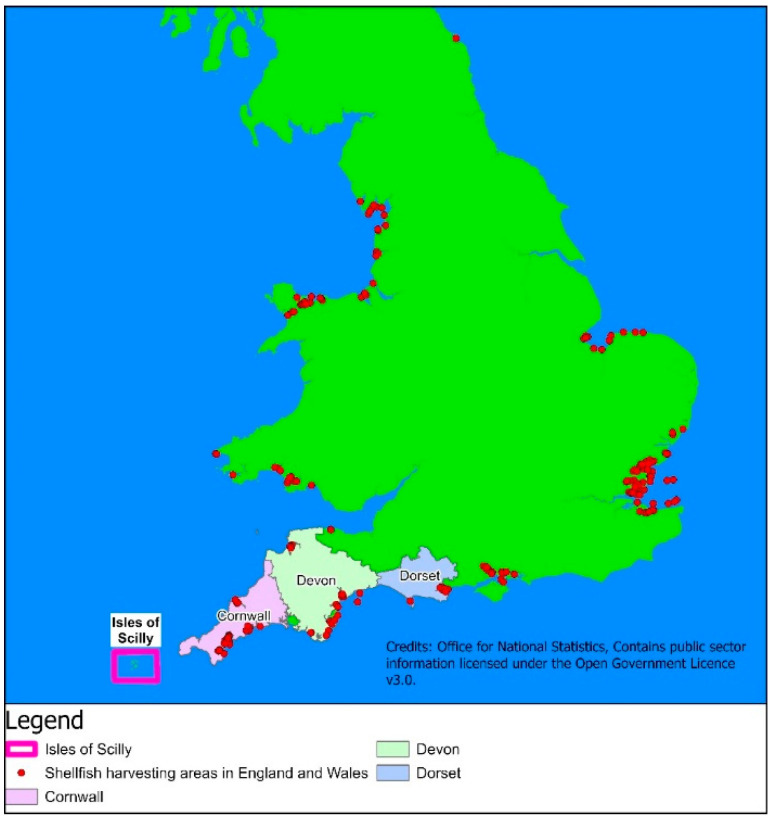
Map showing the classified harvesting areas in England highlighting the location of the Isles of Scilly. Map created in-house using free data from the Office of National Statistics.

As a consequence of the EU legislation dictating the need for official control testing of bivalve molluscs, UK designated shellfish harvesting areas are routinely sampled and monitored for phytoplankton and shellfish toxins. In mainland England, monitoring is conducted across all classified harvesting areas, resulting in the generation of HAB and phycotoxin prevalence data throughout the country (Figure 1). In terms of historical occurrences of regulated marine toxins in England, the majority of HAB and phycotoxin outbreaks have occurred in the southwest. This area has been associated with previous repeated occurrences of both PSP toxins [70] and DSP toxin [63]. In addition, the south coast region as a whole has also been associated with detection of TTXs [50,51], prompting the discovery of TTX in shellfish throughout European waters [52,53]. Similarly, in neighbouring counties, DSP, ASP and PSP toxins have all been routinely detected, with toxin accumulation particularly notable along the south/southwest coast of Ireland [71,72] and the northwest coast of France [73,74].

**Figure 2:**
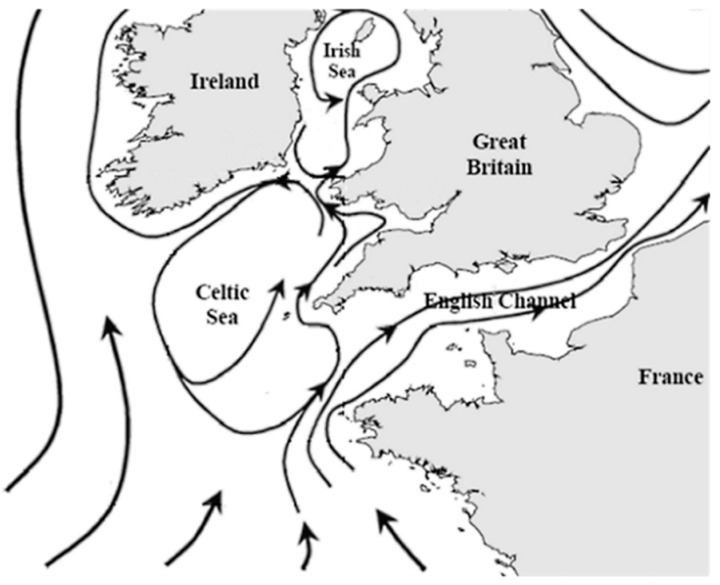
Map displaying prevailing currents around Great Britain, Northern France and Ireland, modified from [79].

The Isles of Scilly (IoS) are the most westerly land mass of GB, being situated approximately 40 km off the southwest peninsula of England in the Celtic Sea within the Atlantic Ocean at the western end of the English Channel and Southern end of the Irish Sea (Figure 1). They comprise an archipelago of approximately 200 granite islands and rocks, separated by shallow seas. Five of the islands are permanently inhabited—St Mary’s, Tresco, St Martin’s, Bryher and St Agnes—with a total population of approximately 2200 permanent residents over a 16 km^2^ area. Main occupations are tourism, agriculture and floriculture, with intense agriculture and/or sewage infiltration resulting in elevated concentrations of nitrate, potassium and magnesium in island groundwaters [75]. The islands are affected by a 2–6 m tidal range with 750–1000 mm rainfall per annum [75]. The seawater is known to be exceptionally pure, classified as ‘super-exposed’ along western rocky shores and more protected along the eastern coasts, facilitating the growth of delicate branch sponges, corals and sea fans, with algae being extremely abundant [76]. Classified by the UK government as a Conservation Area and Area of Outstanding Natural Beauty, there is little evidence to date for the significant increase in population size during the tourist season to have affected the marine environment within the archipelago [76]. The seabed between the islands mostly comprises sand and small pebbles, with lower sized pebbles in the eastern regions in comparison to the west. Whilst nutrient sources into the coastal areas are much lower than those along coastal mainland, the waters still support a diverse marine ecosystem, including numerous fish species, a large colony of Grey Seals (*Halichoerus grypus*) [77] with significant populations of up to ten mega vertebrate species known [78]. The IoS also sit within the circulation of the Celtic Sea Gyre where major currents would normally run past its northern edge; whilst most major currents from the north of France enter the English Channel, there is a weaker flow which runs north across the mouth of the Channel which passes from the northern coast of France, south of the IoS, between the IoS and the coast of Cornwall (Figure 2) [79,80]. Both of these current systems could transfer planktonic microorganisms within this region, connecting the northwestern regions of France with the southwestern coast of GB, the IoS and onwards to the south of Wales and the southeast of Ireland. In terms of HAB occurrences there appear to be interconnections already present within this region, for example, the presence of *Alexandrium minutum* with similar toxin profiles in the NW of France, the SW of England and the S of Ireland and with this toxin profile representing a rare, localized cluster when considered globally [81].

Although there have been historic studies surveying the diversity of macroalgae in the IoS [82], there have been no studies to date investigating the presence of HAB species in the islands. However, previous studies have identified levels of biomass and primary productivity by phytoplankton to be elevated by a factor of ~five around the Isles of Scilly, caused by tidal mixing [83]. A high diversity of marine fauna has been linked to the region being a boundary between cold water and warm water zones containing strong horizonal temperature gradients and frontal systems driving high primary production and phytoplankton blooms, although significant inter-annual variability has been observed in marine environmental variables such as sea surface temperatures, salinity and chlorophyll maxima [84]. In terms of the islands acting as a gateway to the rest of mainland GB for emerging species, the English Channel is well recognized as harbouring high numbers of non-native organisms, with many of these originating from the sea around Japan, where it was noted almost half of the introductions were suspected to originate from Japan due to, in part, to accidental introductions via pathways such as ballast water transfer but also the intentional import of the Pacific Oyster, *Magallana gigas* [85]. Whilst the impact of existing non-native macroorganisms on this geographical region has been well documented, observing microorganisms in the water column poses hurdles that hinder the ability to gather data [85,86,87,88]. In terms of potential non-native microalgal threats, one example is the dinoflagellate genus *Ostreopsis* which has been migrating around the Bay of Biscay [88]. Although *Ostreopsis* is an epiphytic algae, binding to macro algae and the benthos for most of its vegetative life, it is noteworthy that natural and anthropogenic factors can facilitate its transport, for instance biofouling on the bottom of vessels and through floating litter rafts. Overall, therefore there is the potential for experimental observations of emerging HABs in the IoS which may later impact upon classified aquaculture sites within mainland Britian.

Given the geographical location of the IoS, the unique environmental conditions present in the coastal waters and the absence of any previous scientific study of microalgae and/or natural biotoxins in the islands, this study was developed to assess the presence and prevalence of both regulated and emerging HAB species together with their related biotoxins throughout the islands. The aim was to employ a multi-disciplinary approach, incorporating the in-field analysis of water samples using a compact commercial portable microscope, the laboratory-based microscopy of water for phytoplankton detection and enumeration, the application of third-generation sequencing for molecular detection of HAB species and both the quantitative chromatographic analysis and lateral flow screening of marine toxins in shellfish tissues. By focusing on current regulated HABs and toxins, the study aimed to assess the distribution of microalgae and shellfish toxins around the islands, in comparison to those regularly encountered along mainland coasts. The addition of emerging HAB species of concern and related phycotoxins was included to assess the potential for presence of non-regulated toxin threats in the far southwest of GB, before they reach the mainland coast.

In addition to the application of a multi-method assessment of HABs and shellfish toxins in an explored remote territory, the overarching objective was to determine both the current level of risk to local and visiting islanders who frequently recreationally harvest and consume shellfish, to determine the potential for emerging threats in the islands as well as the potential for IoS water and shellfish monitoring to be used as an early-warning sentinel for potentially forecasting HAB and shellfish toxin bioaccumulation that may be subsequently approaching classified mainland coastal aquaculture sites.

## 2. Results

### 2.1. Samples and Sampling Programme

During the first sampling visit to the IoS in March 2020, the five inhabited islands were surveyed extensively and assessed for the presence of bivalve mollusc populations, as well as taking seawater samples in bottles for phytoplankton analysis from the majority of accessible areas throughout the islands. Many samples were taken at this point for baseline indications of phytoplankton and marine toxins outside of the normal expected season for HAB growth and shellfish toxin accumulation.

For the assessment of molluscs and shellfish toxins, the entire length of all beaches, sand flats and rocky shores were visually inspected across all states of the tide during the first sampling visit. During this time, high spring tides were occurring, so low tides were the lowest for the year, enabling sampling to take place far out in the low intertidal zone, including the area between the islands of Bryher and Tresco, which are exposed for only a few hours just twice a year. The inspection revealed a near-total absence of mussels (*Mytilus* sp.), usually the most common bivalve found in wild populations along the English mainland coast. Bivalves found were razor clams (*Ensis* sp.), common cockles (*Cerastoderma edule*) and a variety of clam species, notably surf clams (*spisula solida*) and grooved carpet clams (*Venerupis decussatus*), fitting with previous reports of rayed artemis clams (*Dosinia exolete), Tellina (Moerella) pygmaea* and *Gari depressa* clams, otter shell clams (*Lutaria lutaria*) and razors (*Ensis arcuatus*), in order of prevalence. With such small populations of clams, cockles and razors existing in the islands, no commercial shellfisheries exist and there are no officially designated harvesting areas. The IoS fishery consists primarily of seasonal small-scale potting for primarily European lobsters and brown crabs. The absence of mussels or indeed any large populations of other molluscs may be linked to the excessive dispersal of planktonic larvae which may lead to their loss from the fauna of small islands [89]. Consequently, shellfish toxin analysis was conducted using primarily intertidal cockles and clams found across all five inhabited islands.

The study plan was to include eight separate visits to the islands over the spring, summer and autumn of 2020 for further collection of water and shellfish samples, enabling the systematic determination of harmful phytoplankton cell densities and natural marine biotoxins over the year. However, five days after completing the initial study visit, the UK entered total lockdown as a result of the COVID-19 pandemic, preventing any travel to the islands for the rest of the year. Given that funding was only available for sampling for the remainder of 2020, a revised plan was implemented. Interested locals were contacted through the local school and agreed to conduct Citizen Science, collecting water samples on a regular basis throughout the remaining season. Given that inter-island travel was not permitted, one person was utilized on each of the five islands. Consumables required for collecting and preserving water samples were sent to each of the field support people, and advice provided in relation to collecting and storing samples. Sample numbers had to be kept relatively low given that samples had to be stored in residential refrigerators until shipping was possible later in the year. No other form of seawater sampling was possible, such as phytoplankton net collection, given the inability of the team to source such consumables and to ensure such items were used appropriately by non-trained citizens. In addition, the citizen scientists living on the largest inhabited island, St. Mary’s, also agreed to perform two other tasks. Firstly, they were provided with an ioLight in-field portable microscope, to enable the screening of preserved water samples. The operative was supplied with technical and pictorial guidance on HAB identification. Whilst they were not formally trained as per laboratory microscopists, these identification sheets enabled the rapid assessment of the presence of phytoplankton cells and could alert Cefas staff to the potential need for additional sample collection. Furthermore, with the microscopes being powered and controlled by mobile phones, it enabled photographs to be taken in the field and sent to Cefas for qualitative assessment of cell content. Secondly, shellfish samples were collected when possible, storing these whole in shell in a freezer, until shipment to Cefas. Each of the citizen scientists were also provided with sampling sheets to enable the accurate logging of sample details, including geographical location, date and time. At the end of the sampling programme, following the ability of inter-island travel, the water and shellfish samples were collated and stored in a large temperature-controlled ice box, before being transported to Cefas on the mainland for laboratory assessment.

**Figure 3:**
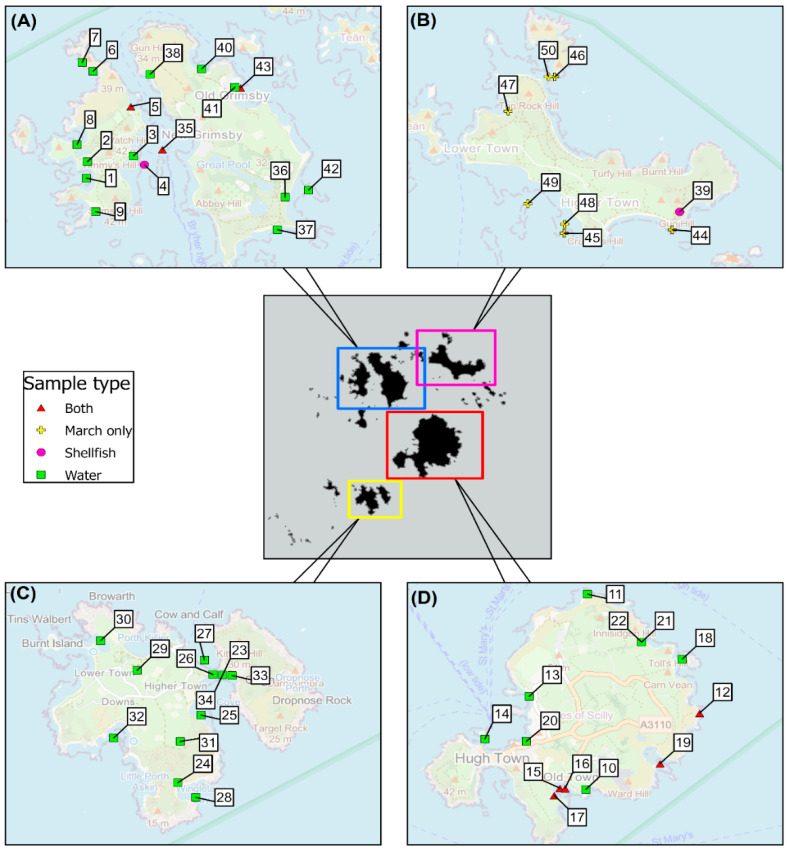
Maps showing the Isles of Scilly (centre) with individual inhabited islands and associated sampling locations in (**A**) Bryher and Tresco (**B**) St. Martin’s (**C**) St. Agnes (**D**) St. Mary’s. Sites are annotated to show whether water samples, shellfish samples or both water and shellfish samples were taken through the study. Numbers on maps refer to sampling locations. Map data © OpenStreetMap contributors, Microsoft, Facebook, Inc. and its affiliates, Esri Community Maps Contributors, Map layer by Esri, Esri UK, Esri, TomTom, Garmin, Foursquare, METI/NASA, USGS.

In total, water samples were collected between the initial sampling visit (March 2020) and October 2020, with samples shipped to Cefas from all except one inhabited island, providing samples from St. Mary’s, Tresco, Bryher and St. Agnes (Figure 3). Bivalve mollusc samples were collected over the same time period from St. Mary’s, as well as bivalves from all five islands taken in March 2020. Additionally, two echinoderms were sampled in March, a seven-armed starfish (*Luidia cilaris*) and a spiny starfish (*Marthasterias glacialis*), given the recent reports of PSP toxins in a variety of benthic marine organisms from the North Sea [90,91]. A follow-up collection of cockles was conducted in St. Mary’s between November 2020 and July 2021 on approximately a fortnightly basis. This second set of samples was kept frozen until shipped to Cefas in autumn 2021. Figure 3 shows the maps of the islands and each of the 50 sampling points utilized through the study for collection of water and shellfish samples. A full list of the site information is given in the Supplementary Materials—Table S1.

### 2.2. Seawater—Microscopy

**Figure 4:**
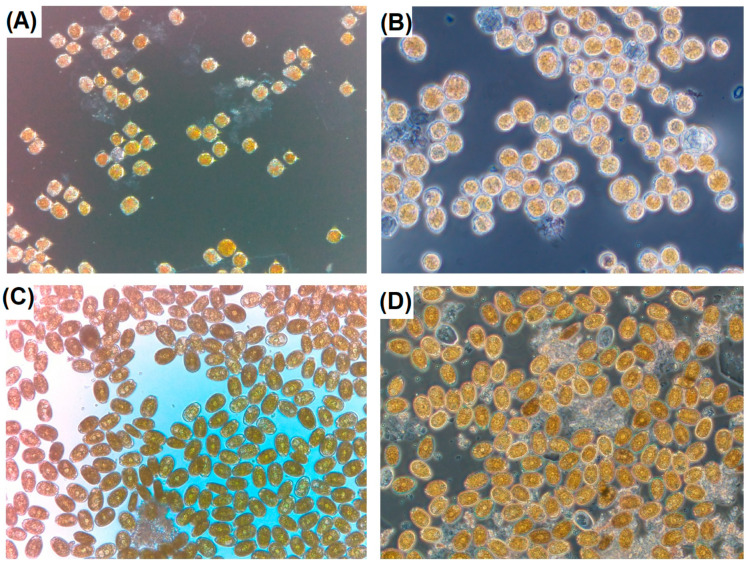
Micrographs of algal cultures demonstrating the images obtained using both the ioLight portable microscope and laboratory microscope (Olympus IX83 inverted fluorescence microscope): (**A**) *Alexandrium ostenfeldii* ioLight (**B**) *Alexandrium ostenfeldii* Olympus *(***C**) *Prorocentrum lima* ioLight (**D**) *Prorocentrum lima* Olympus.

Two types of microscopes were utilized for the examination of seawater samples for the presence of harmful algal phytoplankton genera. The ioLight portable microscope was used in the field and found to provide an easy-to-use and fit for purpose assessment of algae present in Lugol-fixed water samples as well as algal cultures. Figure 4 illustrates the images obtained from the portable instrument in comparison with a laboratory-based fluorescence instrument utilized for routine microscopy. The images obtained enabled HAB genera to be identified in the field when present.

Laboratory light microscopy was used for the enumeration of HAB genera or species which were linked to human health risks. Table 1 summarises the data obtained throughout the year, with mean cell densities across all sampling sites for each determinand. Table S2 in Supplementary Materials displays all cell density data for all samples across the study. The highest cell densities were determined for the *Heterocapsa minima/Azadinium/Amphidoma* group, with cells detected between July and September and reaching a maximum concentration exceeding 7.2 million cells/L during September. In relation to HABs associated with production of regulated shellfish toxins, there was the notable presence of *Alexandrium* sp., *Dinophysis* sp. including *D. acuminata* and *Pseudo-nitzschia* sp., associated with PSP, DSP and ASP toxins, respectively. Mean monthly cell densities were low, ranging from 100 to 400 cells/L for *Alexandrium* sp. and 100 to 750 cells/L for *D. acuminata* with maximum enumerated densities of 400 and 1700 cells/L, respectively (Table S2). Three groups of *Pseudo-nitzschia* species were detected: *P. delicatissima* group (≤4.9 µm), *P. multistriata* and *P. seriata* group (≥5 µm). Cell densities were highest for *P. delicatissima,* reaching close to 5000 cells/L. Higher cell densities were also found for seawater samples taken between June and September, although both *Alexandrium* sp. and *Dinophysis* sp. were detected earlier in the year. *Vulcanodinium* sp. was also tentatively detected in July and September at two different sampling points from St. Mary’s.

### 2.3. Molecular Analysis of Water Samples

#### 2.3.1. Alignment with PR2 Database

In total 57 samples were analyzed using nanopore sequencing, resulting in the generation of greater than ten million reads that passed the Q8 filter threshold. Of those, 1,163,859 reads aligned with entries in the PR2 database and passed the alignment accuracy filtration process. Due to limitations in the specificity for many organisms, it was not appropriate to provide species-level identification using just the SSU region. For this reason, taxonomic identification of raw reads using alignment to PR2 were limited to genus level of specificity. The alignments indicated the presence of over 600 genera, 72 of which being dinoflagellates, several of which were associated with HABs. Alignment results, grouped by month as well as combined, were visualized using Krona, an interactive metagenomic viewer (Supplementary Materials Figure S1) [92]. These data were screened for species associated with HAB events and compared with observations of toxicity and cells in water samples.

#### 2.3.2. Consensus Sequences

**Figure 5:**
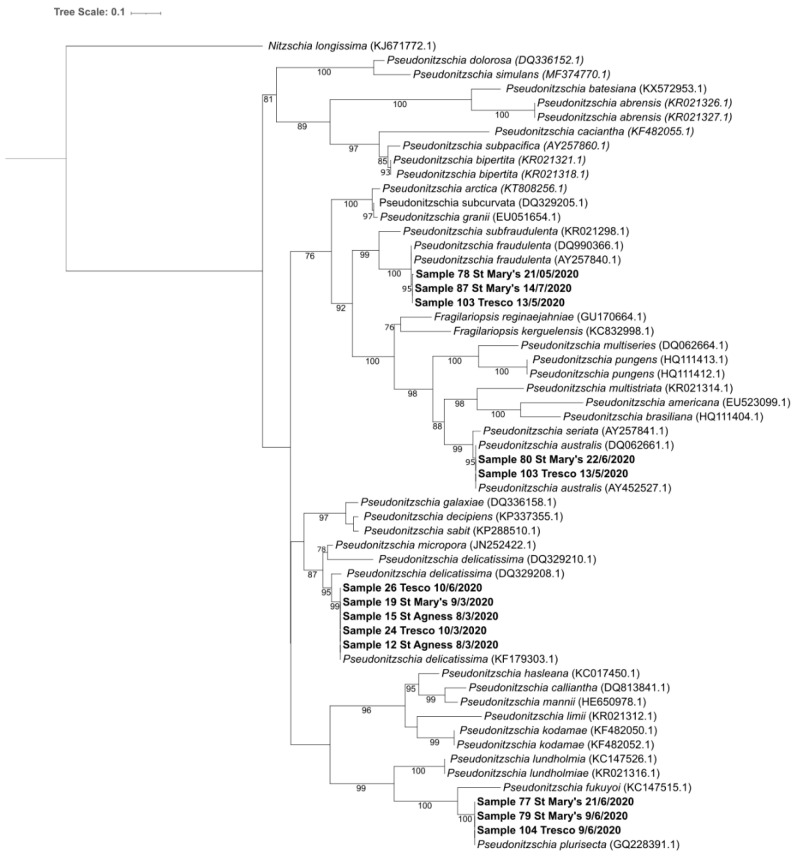
Maximum likelihood (ML) inferred tree of *Pseudo-nitzchia* based on sequences of ITS2 using 10,000 Bootstraps and BS scores of <70 not shown. Branch lengths are shown in scale. Different species colour highlighted, showing sample details and geographical/temporal origins.

Consensus sequences were generated for the microalgae species associated with HAB events using raw reads that had been attributed to genera of interest when aligned against PR2 database. Although primarily dinoflagellates, sequences were also generated for the diatom *Pseudo-nitzschia*, the Stramenopile, *Aureococcus* and the silicoflagellate, *Dictyocha*. Due to the limited number of reads associated with each genera, it was not possible to provide sampling-site resolution, except for *Pseudo-nitzschia*, for which four species were identified, specifically *P. plurisecta*, *P. delicatissima*, *P. fraudulenta* and *P. australis*. The data generated made it possible to provide some indication of spatiotemporal variation. The maximum likelihood phylogenetic tree for this genus is illustrated in Figure 5, showing the distribution of each of the four species throughout the major inhabited islands.

For the potential PST-producing *Alexandrium* sp., only the non-toxin-producing *A. tamarense* was detected, utilizing LSU and SSU-LSU regions with >99.5% identity success. Similarly, from the AZA-producing *Azadinium* genus, the Non toxic variant *A. caudatum var. margalefii* and *A. caudatum* were generated from sequence reads. For the DSP-toxin-producing microalgae, both *Dinophysis acuminata* and two species of *Prorocentrum* (*P. Lima* and *P. spinulentum*) were detected with high confidence. For other HAB species associated with fish health impacts, *Coolia monotis, Karlodinium decipiens* and *K. antarcticum,* together with *Karenia mikimotoi* and *Noctiluca scintillans* were all determined with high detection identity successes. As *Karlodinium* and *Karenia* are both part of the *Kareniacae* group, they were both placed into the same phylogenetic trees, using both ITS1-ITS2 (*K. decipiens*) with LSU (*K. antarcticum*) regions and SSU-LSU (*K. mikimotoi*), respectively. The BLAST results for each of the consensus sequences are summarized in Table 2 and provide alignment metrics for the whole sequence of sections, which was necessary in certain instances due to limitations of the NCBI database. Notably, The ITS2 region was used to speciate *Pseudo-nitzschia* due to this region being notably effective to elucidate. Phylogenetic trees were also generated where possible for dinoflagellate genera, specifically *Alexandrium*, *Azadinium*, *Prorocentrum*, *Coolia*, *Kareniacea*, and Dictyochyceans (Supplementary Materials, Figures S2–S8, respectively).

### 2.4. Shellfish Toxins

#### 2.4.1. Bivalve Molluscs

##### Chemical Analysis

A total of 57 bivalve samples were collected between March 2020 and July 2021, comprising 46 cockle (81%), five carpet clam (9%), three surf clam (5%), two mussel (4%) and one razor clam (2%) samples. Each sample consisted of a minimum of ten animals and were frozen upon collection. After shipment of the frozen shellfish to Cefas, samples were extracted and analysed using validated and ISO17025-accredited instrumental methods for the three classes of EU-regulated marine toxins: ASP, PSP and LTs, incorporating OA group toxins including PTXs, AZAs and YTXs. In addition, a selection of non-regulated toxin classes was measured; TTX and the cyclic imines Gymnodimine (Gym), spirolides (SPXs) and pinnatoxins (PnTxs). Table 3 summarises the quantitative results determined.

Ten out of the 57 samples contained detectable levels of domoic acid (DA) following analysis by liquid chromatography with ultraviolet detection (LC-UV) but with only two of these above the limit of quantitation (LOQ) of 1.0 mg/kg, both being mussel samples. The remaining samples were all cockles containing DA between 0.32 and 0.62 mg/kg. Consequently, the maximum concentration determined equated to just 6.5% of the maximum permitted limit (MPL) for DA in bivalve molluscs (20 mg/kg). No clear pattern was seen in terms of temporal variability of DA presence, with positive samples collected in May, August and November/December in 2020, and low levels continuing intermittently into 2021.

**Figure 6:**
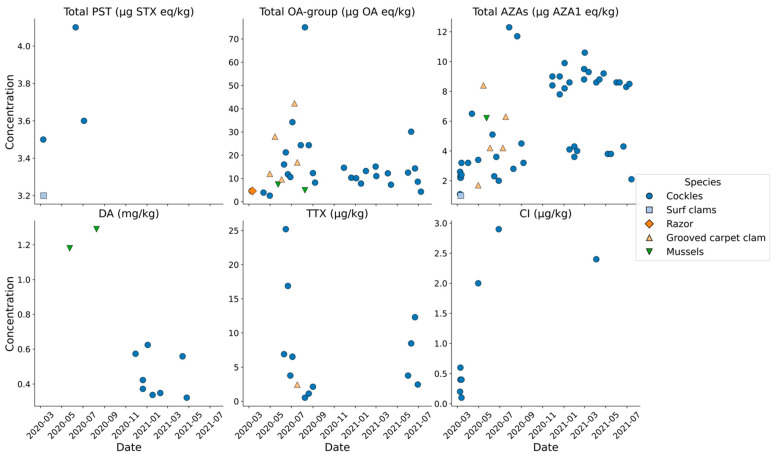
Plots of toxin concentrations quantified in bivalve mollusc samples collected from IoS between March 2020 and July 2021, showing temporal variability = for PSTs, OAs, AZAs, DA, TTX and CI (Cyclic imines).

Analysis for PST was performed initially using the pre-column oxidation LC-FLD method utilized routinely for bivalve mollusc testing throughout the European Union (EU) as well as the UK [60,61]. Following the application of a preliminary semi-quantitative screen after periodate oxidation, no chromatographic peaks were observed in any of the samples so analysis was repeated with the use of a more sensitive method based on hydrophilic interaction liquid chromatography with tandem mass spectrometry (HILIC-MS/MS) [67,68,69]. Only four samples, three cockles and one surf clam, contained detectable levels of toxins, consisting entirely of saxitoxin (STX). STX concentrations were low, ranging from 3.2 to 4.1 µg STX eq/kg, thereby reaching a maximum of approximately 0.5% of the MPL for PSTs (800 µg STX eq/kg). Two of the positive samples were sampled during the first sampling visit in March 2020, with the other two collected in June and July 2020. With no other samples containing PSTs, there was no evidence for any temporal pattern relating to STX presence in molluscs from the islands (Figure 6).

**Figure 7:**
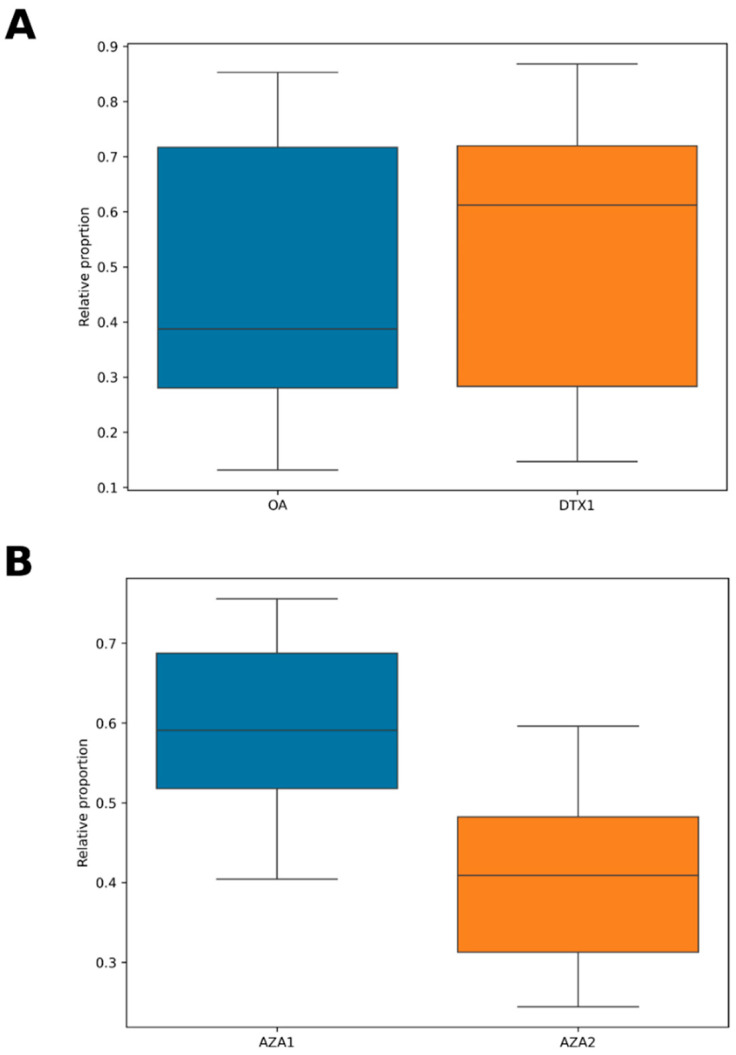
(**A**) Box and whisker plots illustrating medians, interquartile ranges and associated standard deviations for relative proportions of OA group toxin profiles(µg/kg). (**B**) Box and whisker plots illustrating median, interquartile ranges and associated standard deviations for relative proportions of AZA group toxin profiles (µg/kg).

LC-MS/MS analysis of methanolic extracts of the bivalve tissues determined the presence and concentrations of selected regulated lipophilic toxins, including OA group (OA, DTX1, DTX2 and related acyl esters), PTXs, AZAs and YTXs. 35 out of the 57 bivalve samples (61%) contained detectable levels of OA group (DSP) toxins. Concentrations ranged from 2.6 to 76 µg OA eq/kg (mean = 15.6 ± 13.8), therefore reaching a maximum of approximately half of the MPL for OA group toxins (160 µg OA eq/kg). Out of the 35 positive samples, only ten of these contained quantifiable concentrations above the method LOQ of 16 µg/kg, representing 18% of the total samples collected. Figure 6 graphs the concentrations determined and shows the two maxima falling in the warmer summer months of August 2020 (75.0 µg/kg) and June 2021 (30.1 µg/kg). Outside of the June to August summer period, total OA group toxins remained either undetected or at concentrations below method LOQ. OA group toxins detected were OA (48% ± 29%) and DTX1 (52% ± 29%) only (Figure 7), with no DTX2 detected. On average approximately 70% of the OA/DTX1 toxin burden was present in acyl ester form, liberated following alkaline hydrolysis of the methanolic extracts. No PTXs were detected in any of the samples, so PTXs were not incorporated into the OA group toxicity values.

The majority of bivalve samples (91%) contained AZAs, specifically AZA1 (69% ± 19%) and AZA2 (32% ± 21%), with no AZA3 detected (Figure 7). None of the samples, however, contained total AZA concentrations above the method LOQ of 16 µg AZA1 eq/kg, with levels ranging from 1.0 to 12.3 µg/kg (mean = 5.7 ± 3.1 µg/kg). AZAs were therefore detected throughout the entire timeframe of the study with no apparent changes in time as a consequence of seasonal-related environmental factors (Figure 6). No YTXs were detected in any of the bivalve samples analysed in this study. Profile breakdowns of AZA and OA group toxins are illustrated for individual samples in Supplementary Materials.

Cyclic imines incorporated into the LC-MS/MS method included the spirolides (SPX1, 13,19-didesmethyl SPX C, 20-methyl SPX C), pinnatoxins (PnTx A, D, E, F, G and H) and gymnodimine (Gym). The method also included a selection of brevetoxins, specifically BTX B2, BTX B4, BTX B5, PbTx 2, PbTx 3 and S-desoxy BTX B2. Analysis revealed no detection of any of these compounds, with the exception of 20-Me SPX C ranging from 0.1 to 2.9 µg/kg in a total of eight samples (14% of total). TTX was detected in 13 bivalve samples (23%), with concentrations ranging from 0.5 to 25 µg/kg. TTX was the only tetrodotoxin analogue detected in all positive samples analysed. Consequently, the maximum concentration quantified in this study exceeded half of the EFSA-recommended guidance limit for TTX in bivalve molluscs (44 µg/kg [52]) and the regulatory limit for TTXs adopted in the Netherlands [93]. TTX was detected in both 2020 and 2021, the highest levels found in June 2020 (25.2 µg/kg) and June 2021 (12.3 µg/kg) (Figure 6). Only trace levels of TTX were detected at periods outside of June each year (Table 3).

##### Lateral Flow Assays

Given the expense of chemical testing methods and the lack of such resources in the islands, a selection of 20 samples was also analysed using a set of commercial End Product Tests (EPT) based on immunochromatographic Lateral Flow Assays (LFA). The Sensoreal LFAs were used to test shellfish extracts for PSP, ASP and DSP toxin groups with results summarized in Table 4. Qualitative results were all ‘negative’, showing toxin levels below regulatory action limits. Out of these, the majority returned ‘Low’ test results, with the exception of three DSP tests which were run on samples with total DSP toxicity of 34.2, 42.3 and 75 µg/kg and returned LFA results as ‘Medium’.

#### 2.4.2. Echinoderms

**Figure 8:**
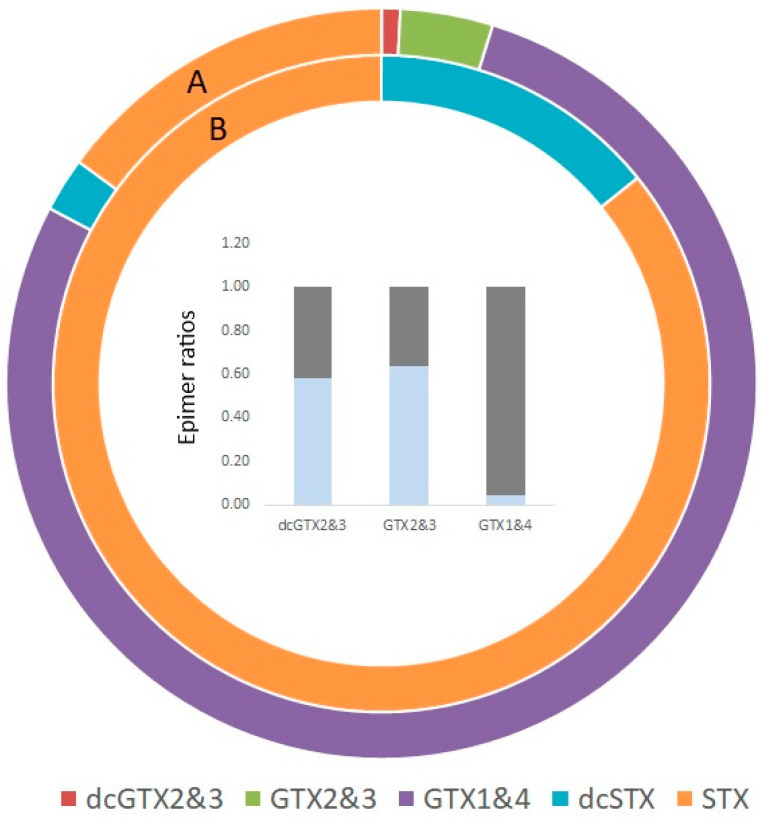
Chart illustrating percentage proportion of each PST analogue (in terms of µg STX eq) for (**A**) seven-armed starfish (**B**) spiny starfish, together with ratio of epimeric pairs (dcGTX2&3, GTX2&3 and GTX1&4) in the seven-armed starfish.

In addition to the 57 bivalve mollusc samples, two echinoderms were obtained from rockpools on Old Town Beach, St. Mary’s, during March 2020. These consisted of a seven-armed starfish (*Luidia ciliaris*) and a spiny starfish (*Marthasterias glacialis*). Both organisms were also subjected to testing for each the regulated and emerging marine biotoxins. No domoic acid was found in either and no LTs apart from a low concentration of OA in the seven-armed starfish (3.1 µg/kg) and 1.2 µg/kg AZA1 in the spiny starfish. Notably, PSTs were quantified in both samples, with total PST concentrations of 273 µg STX eq/kg and 49 µg STX eq/kg in the seven-armed and spiny starfish, respectively. Figure 8 illustrates the profiles of individual PST analogues quantified by HILIC-MS/MS in both samples. The spiny starfish exhibited the simplest profile, with toxins consisting of 86% STX and 14% dcSTX. The profile in the seven-armed starfish was notably different, containing 75% of the total saxitoxin equivalents originating from GTX4, with 15% STX and low relative proportions of remaining GTX analogues. C1&2 was detected in only trace levels (<0.1% of total profile), so is not included in Figure 8. Figure 8 also shows the relative proportions of epimers for each of the pairs detected. Ratios between pairs were found to be approximately 60:40 (epimer a to epimer b) for dcGTX2&3 and GTX2&3, whereas for GTX1&4, 96% of the toxin equivalents was from epimer b; GTX4. No emerging toxins were detected in either sample, including TTX, brevetoxins and cyclic imines.

### 2.5. Other Regions

**Figure 9:**
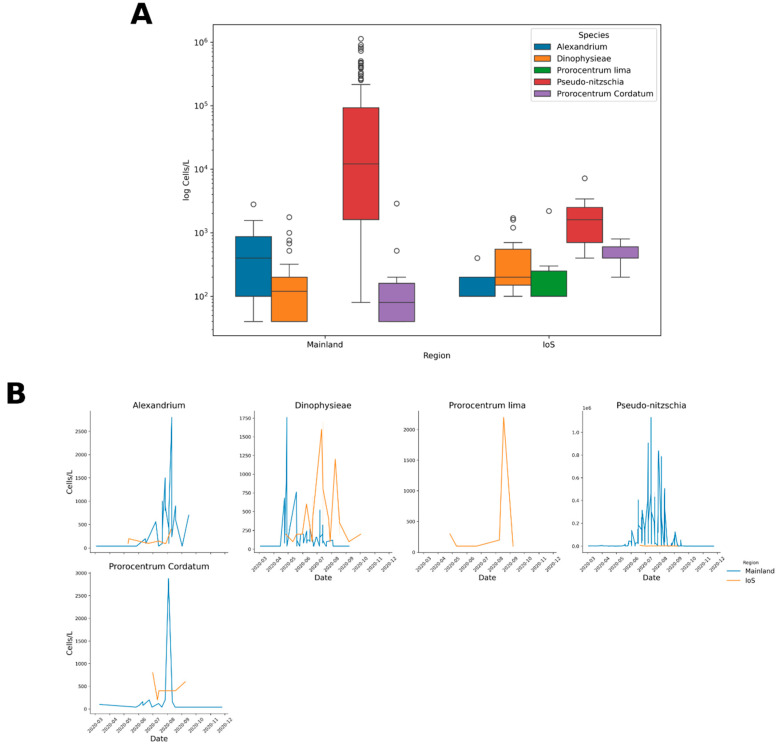
(**A**) Boxplot highlighting the distribution of cell counts for each species in each region. Circles highlight statistical outliers, boxes represent the interquartile ranges and vertical lines represent the median cell counts. The Y axis is log transformed. (**B**) Line graphs represent time series cell counts for each species at each region throughout 2020.

Cell densities and toxin concentrations were compared between the closest mainland shellfish harvesting sites (Figure 1) and those collected and analysed from the IoS. Cell densities were compared using data from 2020 only. Cell densities were not normally distributed (even after log transformation), so non-parametric alternatives were used. To compare the two regions a Mann–Whitney U test was performed; analysis showed a statistical difference in cell counts overall between the two regions (*p* ≤ 0.0001). A separate Mann–Whitney U test was performed to analyse the different species between each region. Analysis showed a statistical difference between *Dinophyceae* sp. (*p* = 0.001), *Pseudo-nitzschia* (*p* = 0.013) and *Prorocentrum cordatum* (*p* = 0.05) cell densities between the two regions. *Alexandrium* sp. were not statistically different between regions (*p* = 0.126). No analysis was conducted for *P. lima* given that no mainland water samples were found to contain this species during 2020. To confirm these tests, a generalized linear model (GLM) was optimized. Data was over-dispersed so a negative binomial model was utilized, which used region, date and species effects on cell counts (*Pseudo-nitzschia* and the mainland region were set as references); this confirmed Mann–Whitney results that cell counts were overall different for regions (*p* = 0.048 and approximately 1.4 times higher in the mainland). The model highlighted *Pseudo nitzschia* had statistically different cell counts to all other species (*Alexandrium* sp., *p* ≤ 0.001 and *Dinophyceae* sp., *p* ≤ 0.001). The model also highlighted that *Pseudo-nitzschia* and *Alexandrium* sp. counts were statistically different between regions (*p* ≤ 0.001 for both). Model output is represented in Supplementary Materials Figure S9. No temporal patterns between regions could be identified. Phytoplankton data is visually represented in Figure 9.

**Figure 10:**
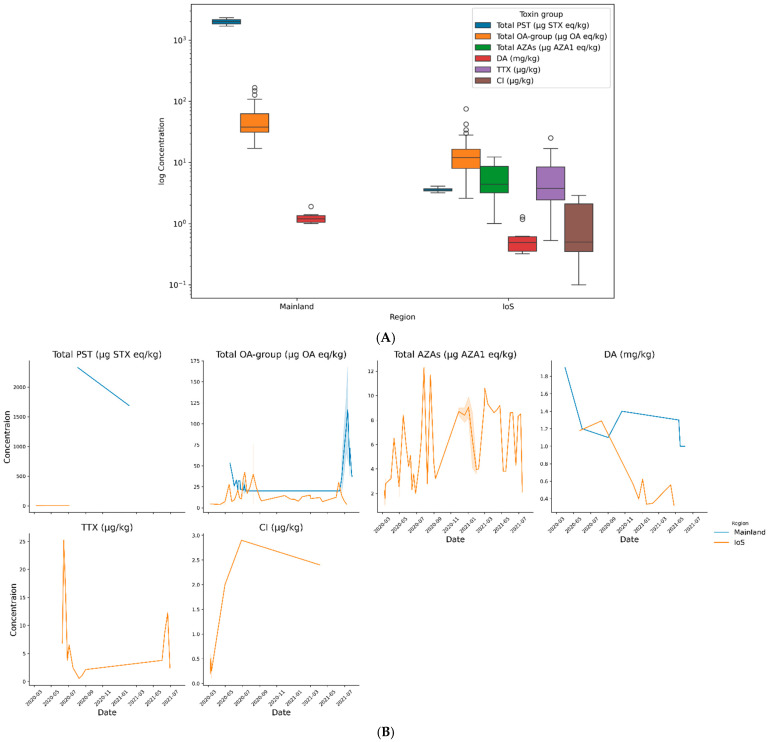
(**A**) Boxplot highlighting distribution of toxin concentrations for each toxin group at each region. Circles highlight statistical outliers, boxes represent the interquartile ranges and vertical lines represent the toxin concentrations. The Y axis is log transformed. (**B**) Line graphs representing time series concentrations for each toxin group at each region throughout 2020 and 2021.

Toxin concentrations between the two regions were compared using data from 2020 and 2021. Toxin concentrations were not normally distributed (even after log transformation), so non-parametric alternatives were used. To compare the two regions a Mann–Whitney U test was performed; analysis showed a statistical difference in toxin concentrations between regions (*p* => 0.001). Separate Mann–Whitney U tests were performed to analyse the different toxin groups between each region. Analysis showed statistical differences between Total OA group and DA concentrations between regions (*p* => 0.001 and *p* = 0.0063, respectively). Analysis showed no statistical difference between regions in PST concentrations, although this did not align with visual interpretation (Figure 10). As positive sample numbers were low (*n* = 6), Mann–Whitney U analyzing the ranks not spread and Mann–Whitney U’s susceptibility to skewed data this result is questionable. To check this analysis a GLM utilizing a gamma distribution and a log link function was performed to test the effect of region on PST concentrations. This highlighted that concentrations differed significantly between regions (*p* ≤ 0.001). It should be noted that although GLM is a better fit, the n = 6 gives the test limited statistical power and results should be interpreted cautiously. Identical GLMs corroborated the Mann–Whitney U results for DA and total OA group concentrations (*p* = 0.0019 and *p* ≤ 0.0001 respectively). All model outputs can be found in Supplementary Materials Figure S10. These results show that toxin concentrations of OA group, DA and PSTs are all statistically different between regions. As TTX, total AZA group and CIs were only detected in IoS, no comparison between regions could be made.

All Mann–Whitney U tests and generalized linear models were performed using the scipy.stats package in Python (Statistical functions (scipy.stats)—SciPy v1.16.2 Manual).

## 3. Discussion

### 3.1. Citizen Science

Although the coincidence of the COVID-19 pandemic and consequent lockdown of the islands preventing further study trips was a significant hinderance to the project, it provided the opportunity to utilize Citizen Science inputs to maintain project integrity. Liaison developed quickly through the one school in the islands, with school alumni and present students all participating actively. Whilst travel to the islands and even between islands was banned, consumables could be shipped and this facilitated the collection of water samples and shellfish and enabled portable microscopy to be conducted in-field, well away from the Cefas laboratory.

The ioLight inverted microscope was found to provide acceptable performance for the detection and identification of HAB genera in-field. Limitations regarding use of this portable microscope related more to the skills and training level of the citizen scientists involved in the study, noting especially that this aspect of the study was forced due to the sudden lockdown and isolation of the islands, with no chance of training in person in advance of the project start. Nevertheless, the microscope was able to be used to highlight seawater samples of concern, and with further training in HAB identification and the access to a portable microscope with XY stage and counting chamber facilities, rudimentary cell enumeration could be conducted successfully. Furthermore, recent advances in artificial intelligence approaches linked to cell identification and enumeration of cyanobacteria in freshwater samples using the ioLight system have resulted in the rapid field-assessment capabilities for harmful algal species by citizen scientists in the US [94]. With further efforts directed towards Machine Learning identification of marine HABs, these portable systems can become powerful rapid detection tools usable by the general public with minimal training, thus opening up great potential for cost-effective, field-deployable HAB assessments.

For toxins testing, laboratory methods that employ HPLC and LC-MS/MS methodologies are out of scope for any localized testing. Consequently, there would be the requirement for EPT to be conducted using commercial LFAs, which can be easily run by non-scientists with a minimum of basic laboratory apparatus and consumables. In this study, the Sensoreal Alert LFAs successfully reported the levels of elevated risk for increased concentrations of DSP toxins in three of the shellfish samples analysed, whilst providing the useful confirmation of low toxin risks from all other shellfish samples assessed. Given further information being generated on LFA method performance, such assays would provide at least a valuable screening tool for rapid toxin testing of bivalve molluscs in the islands.

### 3.2. Seawater Analysis

The molecular data generated from the seawater samples presented here builds on the previous endeavours to develop the technology for application to HAB analysis [66]. Since these data were generated, the chemistry and flow cell used in the methodologies have been improved to generate better accuracy raw reads. However, developments of the bioinformatic pipeline remain relevant to both current and future development. By first aligning the SSU region against a refined version of the PR2 database it was possible to provide genus-level identification [95]. Sequences attributed to genera of interest could be extracted and submitted for further alignment against reference lists of barcode regions of that genus or used directly to generate consensus sequences using the de novo approach provided by NGSpeciesID [96]. Once generated, the consensus sequences had to be aligned and trimmed as the process sometimes included chimeric sequencing artefacts. This was highlighted by interrogation of the BAM files using Integrative Genomic Viewer (IGV) [97]. Once trimmed, the full length (~3.2 kb) consensus sequences did at times prove challenging regarding the assignment to specific organisms. BLAST (https://blast.ncbi.nlm.nih.gov/Blast.cgi, accessed on 2 August 2025) alignment of this relatively long sequence against the NCBI database often returned misleading results. This could be due to the high proportion of short entries on the NCBI database compared to the consensus sequence being generated from the long-range amplicon. Full-length references may provide low variation over the whole length but significant differences within specific barcode regions. Because of these factors, in many instances it was more appropriate to use specific barcode regions of the rDNA rather than the full amplicon length. The results of these alignments against NCBI database are shown in Table 2. It is important to acknowledge the limitations of the current process, in that consensus sequences generated that did not align with any reference sequences were discarded. It is likely that many of these sequences represent examples of previously uncharacterized species or subspecies. It is postulated that these will include cryptic, picoplankton and symbiotic species that are hard or not currently possible to identify, isolate and/or culture. This therefore means that data sets like this can and should be revisited periodically to retrospectively review distribution of newly discovered species.

### 3.3. Pseudo-nitzschia sp.

The potentially toxic diatom genus *Pseudo-nitzschia* was observed more than any other HAB organism, in terms of total reads aligned, the number of samples found to have the genera and their diversity. This abundance of data permitted higher resolution of data analysis shown in Table 2 and Figure 5, allowing comparison of which species were at each location. This is not entirely surprising as this genus is known to bloom in high densities and consists of multiples species [98]. Of the four species of *Pseudo-nitzschia* detected in the study, *P. australis* is the organism most likely to be associated with DA detected in the shellfish from this study, given previous published reports of showing it produces orders more DA than other *Pseudo-nitzschia* species in specific conditions including nutrient and the presence of compounds associated with predatory organisms [99,100]. Furthermore, studies in Scotland have highlighted that *P. australis* is the primary causative organism of DA bioaccumulation and shellfish harvesting area closures and it may be that these are the primary source within the islands, although it was not possible to confirm this [101].

### 3.4. Alexandrium sp.

The reads attributed to *Alexandrium* generated a consensus sequence found to align nearly perfectly with references of *A. tamarense*, (Table 2 and Supplementary Materials Figure S2); this was not unexpected, given that the species was first identified in and derived its name from the River Tamar, located approximately 160 km east northeast of the islands [102]. Since it was first identified there have been multiple amendments to the taxonomy relating to the genus *Alexandrium*, with the current taxonomic status being resolved from within a clade of the *Alexandrium tamarense* species complex to being considered as a non-toxic species therein. This species complex also contained the toxigenic species *Alexandrium catenella* with the taxonomic redescription adding clarity to the genus [103,104] accepted in [105], highlighting that this was not a potential source of toxins detected in the shellfish. Interestingly, there was no detection of *A. minutum*, *A. catenella* or *A. ostenfeldii,* which are linked directly with PST production in UK regions [106,107,108]. The toxin profile seen in the samples is not consistent with the profile of *Alexandrium minutum*, the species associated with toxin events in the southwest of the UK [70,109]. However, it should be noted that the very low levels detected in the samples from this study could result in an atypical toxin profile, as the method varies in the limit of detection for different analogues. It is therefore not possible to draw substantive conclusions on source from the limited toxin profile data available.

### 3.5. Azadinium or Amphidinium

The consistent yet low levels of AZAs in shellfish highlighted the likely presence of known toxigenic Amphidomataceae, either *Azadinium* or *Amphidinium* genera [110]. However, the consensus sequence generated indicated the presence of *Azadinium caudatum,* a genus currently not thought to be toxic [111]. This conclusion was evidenced by BLAST analysis of both ITS regions and LSU separately both identifying variants *Azadinium caudatum* (Table 2)*;* in addition, a phylogenetic tree was generated using the ITS1–ITS2 region (Supplementary Materials Figure S3). The ITS regions were selected over the LSU as although both regions have been shown to be effective for species elucidation, the LSU requires D1-D3 regions and only D1 to D2 is available in the amplicon. It is noted that the study also generated numerous sequences that aligned well with *Azadinium* and *Amphidinium* references during the PR2 alignment that could not be identified as being any of the known species. It could be that these are from an as yet unidentified species of *Amphidomataceae* and the source of the Azaspiracids detected in the shellfish. Further work with dinoflagellate cells involving culturing, sequencing and toxin determination would be required to further examine this hypothesis.

### 3.6. Dinophysis sp.

Although it was possible to provide species-level identification for *Dinophysis*, rDNA is not phylogenetically informative [112,113]. This is due to the presence of paralogues which can have different evolutionary histories. For this reason, no phylogenetic tree was generated for the consensus sequence elucidated from the reads aligning with the genera. However, the BLAST analysis against the NCBI database identified the sequence as all but identical to a *D. acuminata* isolate from the north Atlantic (Table 2), accession: MK860896.1 [114]. This correlates with the speciation via light microscopy and provides strong evidence that this identity is accurate, and that this species is likely to be the source of OA detected throughout the study period within the samples from Scilly. The presence and impact of this species in the islands is well supported by the common occurrence of the species and its associated toxins in nearby UK waters [63].

### 3.7. Prorocentrum sp.

Initial analysis of data highlighted that the epiphytic-benthic dinoflagellate genus *Prorocentrum* were present. The resulting consensus sequences generated were positioned in a phylogenetic tree based on the example published by [115]. This analysis highlighted the presence of *P. lima* and *P. spinulentum* (Supplementary Materials Figure S4)*. P. lima* is known to occur in various habitats of the UK and a study of organisms isolated from SW England indicated its production of OA and DTX1 but not DTX2 [116]. The presence of *P. lima* in water samples is likely a result of the near shore and shallow nature of the sampling locations accessible within this study; given its benthic nature it would not be expected to be seen in water column samples taken further offshore. This aligns with the DSP toxin profiles quantified in the shellfish samples from this study, indicating *P. lima* could be the source along with *Dinophysis,* which is implicated in the vast majority of shellfish contaminations with DSP toxins. The identification of *Prorocentrum spinulentum* in the Isles of Scilly is interesting given its recent identification [117] and its North Atlantic origin [117]. At time of publication, no data was available on toxin production for *P. spinulentum*, so it is unknown if this species is contributing to toxin bioaccumulation in the region. It is also noted that cells identified using light microscopy as *P. cordatum/balticum* were visually very similar to published examples of *P. spinulentum* and undertaken before discovery of the species had been published; as such, it was not considered at the time. It is therefore likely these cell counts are in fact *P. spinulentum*.

### 3.8. Coolia sp.

The high BLAST scores for all sections of the consensus sequence highlight that *Coolia monitis* is likely present in the environment. However, the start of LSU is known to be the best region to provide species-level identification [118]. Therefore, a phylogenetic tree was generated using this region which confirmed the identification and can be found in (Supplementary Materials Figure S5). The presence of the *Coolia monotis* in the waters surrounding the Isles of Scilly represents a significant and novel finding, as the only two previous records are from 1976 and 1981 [119,120,121]. This species, belonging to the family *Ostreopsidaceae*, is known for its potential to produce Cooliatoxin, which has been found to be toxic in mice albeit with the high estimated LD50 of 1 mg/KG [122].

### 3.9. Kareniaceae

There is no clear optimal region of the rDNA for identification of species within the *Kareniacae* family, which includes genera of *Karlodinium* and *Karenia,* that were both observered in this study. Researchers use either ITS1-IT2 or LSU D1-D2 regions [123,124]. For this reason, these sections were submitted separately for both Phylogenetic analysis and BLAST alignment against the NCBI database with results shown in Table 2 and Supplementary Materials Figures S6 and S7).

### 3.10. Karlodinium sp.

The consensus generated from reads attributed *Karlodinium* was found to align perfectly with the LSU of a reference sequence for *Karlodinium antarcticum* (EF469234.1), a species discovered relatively recently in the southern hemisphere [125] (Table 2 and Supplementary Materials Figures S6 and S7). It is noted that in De Salas’ study, the species is described as a “sister species to *Karlodinium decipiens*”, which is what the ITS regions aligned best with. It is also noted that only the LSU for *K antarcticum* is available on NCBI database, which would account for the ITS regions aligning best with a different species, and why the ITS tree did not include this species (Supplementary Materials S7). Due to the recent discovery of the species, no data relating to toxin production could be found; however, the sister species, *K. decipiens*, has been found to be able to produce the ichthyotoxic Karlotoxin [124].

### 3.11. Karenia sp.

Reads attributed to the genus Karenia generated a consensus sequence that ITS and LSU regions that aligned well with reference sequences of *K. mikimotoi* (Table 2 and Supplementary Materials Figures S6 and S7). *Karenia mikimotoi* is globally distributed and creates blooms so large they can be visualized using satellite imaging [126,127]. This species is known to reach high concentrations in blooms in the western English Channel [128,129]. Although not toxic to humans, dense blooms of this and other species within the genus have been associated with mass mortalities of marine organisms [126,130]. Mortalities can occur due to the production of ichthyotoxic and hemolytic compounds (Gymnocins), as well as via oxygen depletion, disruption of food webs, disruption of water chemistry and prevention of light penetration [128].

### 3.12. Dictyochyceans

Two consensus sequences were generated from the class of Dictyochophyceae: *Dictyocha speculum* and *Aureococcus anophagefferens.* This finding was supported by both the multi- region BLAST alignments shown in Table 2 and the phylogenetic analysis found in Supplementary Materials S8. These heterokonts are commonly referred to as silicoflagellates (order *Dictyochales*). The SSU (18 S) gene can be used to identify them within the order [131]. Both *Dictyocha* and *Aureococcus* are known for their potential harmful effects on marine life. However, there has been no evidence to prove that either species are toxic. High concentrations of *Dictyocha* can damage fish gills due to their sharp spines [132]. *Aureococcus anophagefferens,* colloquially referred to as brown algae because of the brown blooms they cause, can also harm aquaculture as well as the wider environment [131]. Although *Dictyocha speculum* has previously been identified in UK waters, its presence can be considered unusual due to there being only a single published account [133]. Although it is commonly observed on the west coast of Canada and Denmark, its occurrence at the Isles of Scilly is noteworthy and potentially significant for further study and monitoring.

### 3.13. Noctiluca

*Noctiluca scintillans*, a species renowned for its bioluminescence, is the only representative species of the genus *Noctiluca*. Its genetic divergence and large size make visual and molecular identification easy, both of which positively identified its presence in this study. All sections of the amplicon from 18 S to D2 of 28 S can be used for speciation [134] which when submitted for BLAST analysis provided a 99.63% identity with 94% coverage, with the next best alignment being 25% divergent. Although not a toxic organism, *Noctiluca scintillans* can cause dense surface scums, harming fish and other marine organisms. The concentrations detected in this study are unlikely to cause issues in the environment, noting also that it was only observed rarely in samples assessed by microscopy. Noctiluca occurrence is thought to be associated with anthropogenic increases in nutrient inputs. The species exist in one of two forms, red and green, with red being its natural heterotrophic state and the green variant containing the photosynthetic symbiont *Pedinomonas noctilucae* in its vacuole. When in this red state the increase in nutrients stops being the driver for growth and is replaced with abundance of prey. It was noted that *Pedinomonas noctilucae* was not detected with the sequencing tools applied, so it is suspected that the organism was living in a heterotrophic state.

### 3.14. Indeterminate Dinoflagellates

Within one water sample from St. Mary’s, collected in July 2020, there were several dinoflagellate cells present when observed by light microscopy which shared gross morphological characteristics with the toxic dinoflagellate *Vulcanodinium rugosum.* Unfortunately, it was not possible to obtain a plate press of the cells observed, and consequently we were unable to confirm identity. The cells observed may therefore have been *V. rugosum,* or one of the morphologically similar members of the Peridiniales family. Since its classification in 2011 [135], *V. rugosum* has been found to be a producer of pinnatoxins, a potent neurotoxin group, with rapid effects in injected mice but currently no known human poisonings [136]. The analysis undertaken in this study did not provide evidence of high levels of pinnatoxins, indicating this may have been a morphologically similar species.

Overall, this study has highlighted that the strategic use of nanopore technology is a powerful research tool but is affected by the presence of off-target organisms. This in turn caused poor sensitivity in some instances and accounts for some limitations in correlation with light microscopy data. This means that many samples need to be analyzed to maximize inclusivity. The “one primer set for all” approach, adopted in this and another earlier study [66] can be overcome by the use of semi-targeted primers that target a single genus or family of interest, an approach that has proven effective for the accurate identification of *Alexandrium* [65,66]. The use of classic techniques, such as the Utermöhl method, alongside the nanopore sequencing allowed for sensitive screening of water samples, enumeration of species observed via microscopy and accurate identification of many genera and/or species present in the waters around the IoS via sequencing.

### 3.15. Shellfish and Biotoxins

#### 3.15.1. Bivalve Molluscs

Following application of the validated instrumental detection methods for a wide range of regulated and non-regulated shellfish toxins, results demonstrated the general low level or non-detection of toxins in the majority of samples. Only low levels of the hydrophilic toxins, DA and PSTs, were detected, with maximum concentrations measured equating to a very small percentage of the MPL safety limits for bivalve mollusc consumption as defined in EU law [16,17]. This very low detection therefore appears to fit well with the sequencing results showing only the presence of the non-toxin-producing *A. tamarense,* and consistently low levels of the potentially toxic *Pseudo-nitzschia* genus. Out of the LTs monitored, AZAs were seemingly present in almost all the samples analysed, but again at concentrations below reporting limits and well below MPL thresholds. This was also unsurprising given the infrequent detection of *Azadinium* sp. following nanopore sequencing analysis, although it has been demonstrated that mussels are able to absorb dissolved AZAs [137] and they may be persistent in shellfish once contaminated [64]. The notable presence was of the DSP-causing OA group toxins, with OA and DTX1 quantified in approximately 60% of the samples analysed. Whilst the majority of these contained low levels of toxin, one sample reached 76 µg OA eq/kg, therefore close to half the MPL health threshold for DSP toxins of 160 µg OA eq/kg. Toxin determination of many of the shellfish samples is understandable given the confirmation of the presence of *Dinophysis acuminata* and *P. lima* by both microscopy and molecular methods. In terms of human health limits, whilst shellfish containing such DSP levels are deemed safe to eat, there were indications that there is the potential for DSP toxin accumulation in shellfish from the islands. Ongoing monitoring would be required to determine whether these levels are a realistic indication of toxicity from year to year. The profile of OA group toxins quantified consisted entirely of just OA and DTX1. This contrasted notably with the OA group profiles encountered in mainland GB, including SW England, where profiles are normally dominated by OA due to the toxin profile within the causative *Dinophysis acuminata*, with increasing concentrations of DTX2 usually found later in the year when *Dinophysis acuta* is more likely to proliferate [63,138]. DTX1 is rarely encountered in the UK mainland inshore shellfish harvesting areas, and when it is detected accounts for very low proportions of total OA group toxin proportions. Therefore, the mean DTX1 proportion of 52% ± 29% represents a notably different profile to that determined in mainland shellfish since the LC-MS/MS method was implemented for regulatory testing in 2011. Such differences could relate either to the presence of different dinoflagellate species that were undetected by the sequencing methods, different strains of *Dinophysis acuminata* in the water column that produce DTX1 such as those reported from other parts of Europe [38], impacts of the life cycle stage of the *Dinophysis* cells which may affect the proportional contribution to the overall toxin profile for different congeners [139] and/or the effects of toxin metabolism in the cockle samples analysed predominantly in this study. Further work would be required to assess the toxin profiles present in cultures of *Dinophysis* and/or *Prorocentrum* species isolated from the islands as well as shellfish feeding studies to assess any changes to profiles once mass cultured phytoplankton is fed to local bivalve species.

Whilst no lipophilic emerging/non-regulated marine toxins were detected at quantifiable levels in any of the samples, there was the notable detection of TTX, which was found in bivalves during the summer of each year, particularly in June. Maximum concentrations reached half of the EFSA-recommended health threshold level, so would not constitute a health risk to local consumers of cockles and clams based on the results from these samples alone, but the data do indicate that under different conditions there is the potential for these harmful neurotoxins to be present in wild-harvested food products. Previous work has demonstrated the occurrence of TTX in multiple shellfish harvesting areas throughout SW England [49,50,51,140,141]. Maximum concentrations of TTXs to date in England have reached 253 µg/kg, over five times higher than the EFSA 44 µg/kg guidance limit, but to date these were only detected in Pacific oysters (*Crassostrea gigas*), native oysters (*Ostrea edulis*), mussels (*M. edulis*) and hard clams (*Mercenaria mercenaria*). As such, this represents the first report in the UK or even globally of the detection of TTX in the common cockle (*Cerastoderma edule*) and the grooved carpet clam (*Venerupis decussatus*), expanding the number of species for which TTX accumulation is now recognized [53]. Given the variability in TTX concentrations from year to year in SW England, the quantitation of TTX in the IoS provides a warning that in future years, there is the potential for high concentrations of TTX to be present. In contrast to the other marine toxins discussed in this study, TTXs are thought to be produced by and/or associated with the presence of certain marine bacterial species, including *Vibrio* sp. and *Bacillus* sp. [51,142,143], although to date no reliable production of TTXs has been demonstrated and the full biosynthetic pathway has not been proven. To date, though, no screening has been conducted in the IoS for the presence of *Vibrio* sp., and/or other potential TTX-producers, and the methodologies employed in this study were not used to target bacterial presence. Further work would be required to determine populations of harmful marine bacteria, through a combination of both microbiological and molecular assessment. In addition, TTX may also be present through the presence of TTX-bearing organisms which are known to be invasive to the UK, such as the marine nemertean *Cephalothothrix Simula*. Specimens of this species, originating from the seas around Japan, have been found in SW England, including the far west of Cornwall, so are highly likely to be also present in the rock pools around the IoS. Such species have been shown to contain extremely high concentrations of TTX which could feasibly transfer to scavenging marine organisms or may be present within the shells of filter-feeding molluscs [141,144].

In addition to the chemical analysis methodologies employed for toxin testing, a set of commercially available LFAs were also utilized for sample testing, given their applicability to use by non-trained citizen scientists in the field. The Sensoreal Alert LFAs are portable assays that were found to provide rapid analysis capabilities, with the LFA strips being read on an electronic scanner which returned either a ‘Low’, ‘Medium’ or ‘High’ test result, depending on the intensity of the test lines on the LFA strips used. According to the manufacturer, Low and Medium results equate to samples below the regulatory action limits, with ‘High’ results indicating shellfish which contain total toxin levels close to or above these limits. Furthermore, ‘Low’ results are interpreted as containing no or very low toxin levels, whilst ‘Medium’ results indicate higher levels but below safety limits. In this assessment, none of the 20 samples analysed for ASP, PSP and DSP returned positive results (‘High’), with the vast majority showing ‘Low’ results. The three DSP samples returning ‘Medium’ LFA results corresponded to the three samples containing the highest DSP toxicities, with actual results of 34, 42 and 75 µg OA eq/kg. Medium LFA results are interpreted as containing total toxicity below regulatory limits but should be considered by operators as containing levels of toxins which may potentially increase in a relatively short space of time. Here, the LFAs effectively demonstrated the usefulness of rapid portable testing for monitoring shellfish toxins in bivalves, but further work would be required to thoroughly assess the performance of these assays through robust validation studies, including their cross-reactivity to other OA group toxins, notably the DTX1 which dominates the shellfish toxin profiles measured during this study.

#### 3.15.2. Echinoderms

Recent studies have reported the occurrence of PSTs in a wide range of non-bivalve benthic organisms, most notably with the quantitation of high concentrations in both sunstars (*Crossaster papposus*) and the bryazoan Sea Chervil (*Alcyonidium diaphanum*), as well as reports of toxins in up to twelve new species associated with the saxitoxins [90,91]. Whilst the majority of these organisms are not seafood products destined for human consumption, the presence of toxicity in these organisms does provide other risks including the trophic transfer of toxins to higher-level organisms that are eaten and the potential for significant risk to the health of animals [145]. In this study, both starfish species tested, the seven-armed starfish (*Luidia ciliaris*) and a spiny starfish (*Marthasterias glacialis*), contained PSTs, with the highest concentrations present in *L. ciliaris*. This provides further evidence therefore for PSTs in *L. ciliaris* as reported previously in the North Sea [90] as well as the first known report for PSTs in *Marthasterias glacialis*. Both creatures are predators and scavengers of other echinoderms and smaller marine organisms, as well as dead organic matter. As such, the presence of PSTs in these organisms, in contrast to the general absence of toxins in bivalve molluscs, may indicate that the toxins originate from a non-phytoplankton source, as postulated previously [90,91]. Further evidence for this can be seen by the dcSTX/STX profile in one of the starfish, also seen in many of the benthic organisms previously studied in the North Sea in areas where *Alexandrium* species were not observed or at times of the year when blooms were not expected. The dominance of GTX4 in the profile of the seven-armed starfish is interesting, as this has not been found before in any bivalve or non-bivalve organism around the UK. The profile may therefore either represent a novel source of toxins and/or be related to species-specific toxin transformation.

### 3.16. Links to Mainland

Where harmful microalgal species were found to occur in the waters of both Scilly and the mainland, there was a similar pattern of seasonality. For *Alexandrium,* concentrations were lower in the islands when compared with the mainland, but the most consistent occurrences coincided with the most consistent observations being in mid–late summer. The presence of *Dinophysis* is the most noteworthy comparison between the two locations, with the Isles of Scilly having similar or higher cell concentrations present, and with those occurring later in the year. Whereas in the classified mainland shellfish harvesting areas, cell concentrations were at their highest in May, persisting at lower levels into the summer. For the Isles of Scilly, *Dinophysis* became more common in the summer, with peak abundances recorded in the mid–late summer. This may indicate that this genus is carried south, away from the south coast of England before reaching the archipelago. However, the water circulation and tidal streams around the islands are highly complex, given the position of the islands at the edge of the continental shelf and being subjected to powerful tidal flows throughout its highly varied bathymetry. Consequently, it was difficult to understand whether water circulation would likely result in HAB species from the islands reaching mainland Cornwall at any given time, or vice versa. However, with the tidal streams typically flowing west to east, there would be the hypothetical likelihood of HABs reaching Cornwall, rather than flowing back to the islands from the mainland [146,147].

One notable tidal influence is the presence of seasonal “jet-like” circulation feeding water from an area approximately 700 km west from the Scilly isles into the Celtic Sea and around the west coast of Ireland; this can be seen in Figure 2 [79,80,82]. This may mean the Scilly Isles could function as a steppingstone habitat, facilitating transport of invasive algae to the west coast of Ireland, and could explain localized issues associated with *Azadinium* in the region [148]. This speculation is not supported by the study identifying any toxic variant of the genera. Although there is not documented evidence of this occurring, increases in anthropogenic effects and rising sea temperatures may make this route potentially more viable in the future. The prevailing currents affecting the IoS also link these islands to the north of France, although typical circulation leads to a northward flow of waters and consequently planktonic species, so it could be expected that the transfer of organisms from the Celtic Sea to the French coast is less likely. The region is, however, well connected in other aspects, with it having been noted that recreational boat traffic is high in the region with a strong interconnectedness between the southwest of England and the northern French coast, as well as a lesser connection from recreational boating between the southwest of England and southern Ireland [149], with a large number of trips between England, France and the Channel Islands [85].

### 3.17. Future Human Health Protection in the Isles of Scilly

The data generated in this study has demonstrated the presence of both a wide range of potentially toxin-producing microalgae and the detection of multiple shellfish toxin groups responsible for human shellfish poisoning syndromes. Whilst no classified shellfish harvesting areas exist in the islands, there is anecdotal evidence for recreational harvesting of bivalve molluscs from the intertidal areas of all five inhabited islands, so there is consequently a potential risk to human health of local shellfish consumers if toxins were ever to reach levels above the concentrations stipulated in EU law. No laboratory services capable of HAB microscopy, sequencing and/or chemical testing for phycotoxins exist in the islands, so any future monitoring of bivalve mollusc food safety risks would need to be conducted either remotely in mainland laboratories, or through the actions of local scientists, citizens or other interested stakeholders. With transportation times of samples from the islands taking at least several days and frequently delayed further by bad weather, the use of local testing facilities would be the only practical option for rapid monitoring. Local services focused on HAB and toxin risk management would therefore need to utilize approaches that are suitable for use by people with no access to designated laboratory environments. As such, the combination of the portable microscopy, plankton net sampling and LFA tools when properly validated would provide interested stakeholders in the islands with a set of valuable tools for rapid and responsive monitoring of seawater and shellfish tissues for potential HABs and the bioaccumulation of shellfish toxins.

## 4. Conclusions

This study reports the first comprehensive, multi-method assessment of harmful algae and shellfish toxins in the Isles of Scilly (IoS), combining light microscopy, nanopore sequencing, chemical analysis and immunoassays. The study area was chosen given the absence of any previous attempts to characterize the presence of HABs and their toxin metabolites in this most southwesterly region of the UK. Given the unexpected impacts of the COVID-19 lockdown of the islands, a Citizen Science network was successfully utilized for sample collection purposes as well as trialing the use of the ioLight portable microscope for microalgal identification. Furthermore, the integration of microscopy and molecular data was found to provide a robust framework for understanding the diversity, distribution, and potential toxicity of phytoplankton in this previously unstudied region. This approach demonstrated the presence of many of the known shellfish-toxin-producing HAB genera including *Alexandrium*, *Dinophysis*, *Prorocentrum* and *Pseudo-nitzchia* sp., as well as potential toxin producers such as *Gymnodinium*, *Coolia*, *Karlodinium* and *Karenia* sp. along with other non-toxin-producing HABs such as *Noctiluca* sp. Whilst further work is required to optimize the nanopore sequencing approaches to facilitate routine testing of seawater samples for HABs, the study showed the great potential for use of semi-targeted sequencing approaches to supplement classical microscopy methods. A wide range of regulated and non-regulated shellfish toxins were detected in the cockles, clams and mussels collected between March 2020 and July 2021, but with no regulated toxins quantified above maximum permitted levels established in EU regulations. DSP toxins were found at the highest concentrations, with a maximum of 75 µg OA eq/kg quantified in August 2020 equating to nearly half the regulatory action limit. The DSP profile dominated by OA and DTX1 with the absence of DTX2 indicated either regio-specific toxin transformation or the presence of DSP-toxin-producing microalgae with a previously unreported toxin profile. TTX was also detected in multiple samples, with a maximum concentration of 25 µg/kg exceeding half the regulatory limit imposed in the Netherlands. This also provides the first report of TTX in grooved carpet clams and cockles in the UK. Consequently, whilst all shellfish tested in this study would be classified as safe to eat, these data do provide indications that there is the potential for diarrhetic and neurotoxic compounds to accumulate in shellfish tissues in the islands and that some form of monitoring would be recommended during the summer months of the year. Results also evidenced the presence of PSTs with unusual toxin profiles in two echinoderm samples, with this being the first report of saxitoxins in the spiny starfish (*Marthasterias glacialis*), highlighting the potential for trophic transfer of neurotoxins into the trophic web. Overall, this multi-disciplinary study highlighted the usefulness of Citizen Science inputs to a future systematic monitoring programme in the islands, utilizing both portable microscopy and rapid toxin test kits. Such an approach could not only benefit local shellfish consumers, but also potentially provide early warning indications of future shellfish toxicity outbreaks along the SW coasts of the UK. More monitoring work would be required over a longer time period to establish a more robust baseline of HAB and toxin occurrence, as well as designing a more specific set of molecular methods for HAB speciation and enumeration, together with a digital tool set which incorporates AI-driven assessment of portable microscope images to aid the citizen-led monitoring of shellfish waters for future increasing HAB occurrences.

## 5. Materials and Methods

### 5.1. Reagents and Standards

Lugol’s iodine was purchased from Merck (Gillingham, UK). For toxin analysis, chemicals were LC-MS-reagent grade where possible, with sample preparation and solid-phase extraction reagents of HPLC grade (Rathburns, Walkerburn, UK). Mobile phases were prepared from LC-MS-grade solvents (Fisher Optima, ThermoFisher, Loughborough, UK). Certified reference materials for purified toxin standards were obtained from the Institute of Biotoxin Metrology, National Research Council Canada (NRCC, Halifax, NS, Canada).

### 5.2. Samples

Seawater sampling was conducted with a 2 m pole sampler, collecting 250 mL water in pre-cleaned and rinsed polypropylene tubes. Given the absence of piers and publicly accessible jetties at the time of the study, samples were collected either by standing on rocks above the sea, or on foot by wading into the sea. Once retrieved, neutral Lugol’s iodine solution was used to fix the samples, which were then kept chilled until analysis could be undertaken. Initial seawater samples were collected during the first sampling visit to all five inhabited islands by Cefas staff during March 2020. Whilst the project plan was to involve multiple follow-up sampling visits, COVID-19 lockdown prevented further sampling. Consequently, local citizen volunteers were utilized to continue the collection of seawater samples. These were collected, preserved and stored in the same way, with samples retrieved from each of the inhabited islands with the exception of St. Martin’s. Each sample was subsequently separated into triplicate 50 mL sub-samples and utilized for onsite ioLight microscopy, laboratory microscopy and molecular testing, respectively.

**Figure 11:**
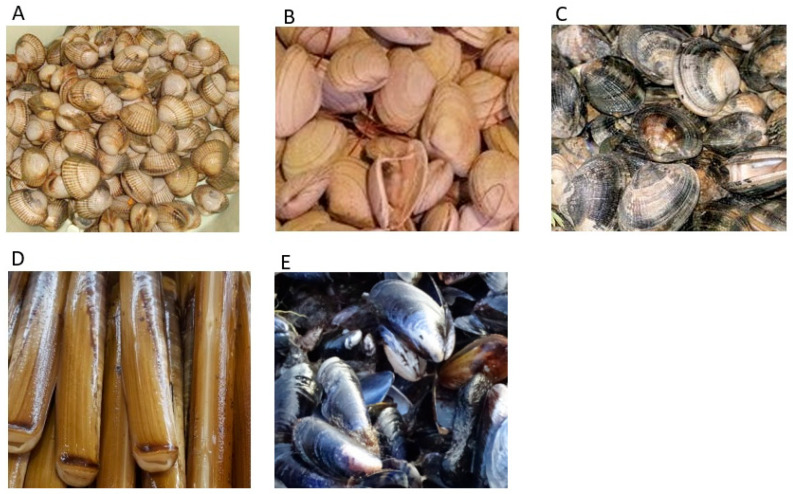
Images showing bivalve mollusc species collected during the study, (**A**)—cockles, (**B**)—surf clams, (**C**)—grooved carpet clams, (**D**)—razor clams, (**E**)—blue mussels.

The initial sampling visit also enabled collection of shellfish samples. These were collected by hand, taking a minimum of 10 animals per sampling point wherever possible. Shellfish species collected were common cockles (*Cerastoderma edule*), surf clams (*spisula solida*), grooved carpet clams (*Venerupis decussatus*) and razor clams (*Ensis* sp.). Blue mussels (*Mytilus* sp.) were collected later in the year (Figure 11). Once retrieved these were bagged and placed into a cool box, containing frozen ice packs. After a sampling day, these were stored frozen, until they were transported back to Cefas. During COVID-19 lockdown, shellfish sampling continued only on the most populated island of St. Mary’s. Collection was conducted in the same way, with shellfish frozen until transportation occurred at the end of the year under temperature-controlled conditions. Due to the relatively low number of shellfish samples collected during 2020, a follow-up shellfish collection was made intermittently during 2021 by a second citizen volunteer. These were shipped to Cefas once all samples had been collected at the end of the summer.

### 5.3. Seawater Analysis

For onsite portable microscopy, the 50 mL fixed seawater samples were inverted several times and allowed to settle for a minimum of 30 min. An aliquot from the settled material of the sample was subsequently pipetted onto a microscope slide and placed into the viewing position of the portable microscope. Qualitative analysis was conducted, focusing on the potential presence of harmful algal species, specifically dinoflagellates of the genera *Dinophysis*, *Alexandrium* and *Prorocentrum*, together with the diatom *Pseudo-nitzschia*.

For laboratory microscopy, Lugol’s fixed samples were placed into either 5 mL, 10 mL or 25 mL Utermöhl chambers and allowed to settle on a horizontal bench at ambient temperature for a minimum of 12 h. Analysis was conducted using an Olympus X70 inverted research microscope (SciQuip, Rotherham, UK) or a Nikon Eclipse TE300 inverted light microscope (Nikon/GT Vision, Wickambrook, UK). Cells belonging to the genera *Alexandrium*, *Dinophysis*, *Prorocentrum*, *Pseudo-nitzschia*, *Karenia*, *Gymnodinium* were identified and enumerated using inverted light microscopy and the cell densities were calculated to express phytoplankton concentrations in cells/L. Other cells from potentially toxic or harmful genera were identified and enumerated where possible, information on harmful genera for this targeted analysis was taken from the IOC_UNESCO Taxonomic Reference List of Harmful Microalgae [150]. Due to the focus of the study, only potentially harmful algal taxa were included in this analysis, and it does not reflect full community abundance and diversity.

### 5.4. Molecular Analysis of Water Samples

**Figure 12:**
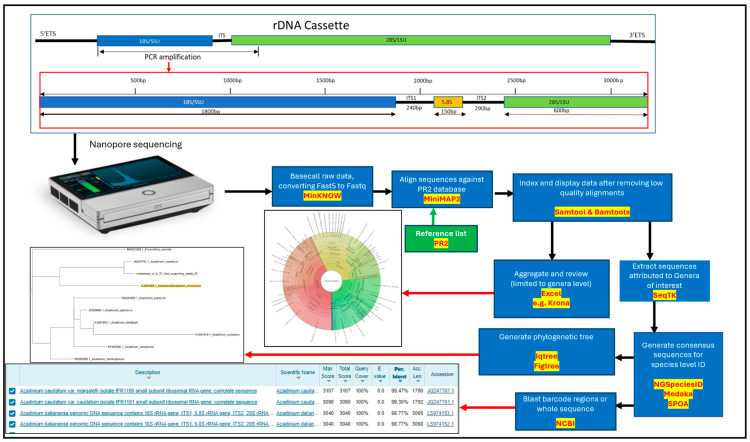
An overview of the molecular techniques used on water samples; blue boxes highlight bioinformatic processes, red text highlights software of resources used.

An overview of the process employed for molecular analysis can be seen in Figure 12.

#### 5.4.1. Sample Preparation and DNA Sequencing

DNA extraction, sample preparation and nanopore sequencing protocol used was based on the previously published manuscript, outlining the application of nanopore sequencing to the study of harmful algal blooms [66]. DNA was extracted from 50 mL water samples using the Qiagen power biofilm kit. Primary long-range PCR reactions were performed on Eppendorf Master Cycler Nexus, (Eppendorf, Hamburg, Germany) with the following thermal regime: 98 °C for 60 seconds, followed by 30 cycles of 98 °C for 10 seconds, 63 °C for 20 seconds, 72 °C for 90 s and a final extension of 72 °C for 10 min using tailed primers (underline is tail for barcoding PCR):

FWD: 5′ TTTCTGTTGGTGCTGATATTGCGCTTGTCTCAAA GATTAAGCCATGC 3′
REV: 5′ ACTTGCCTGTCGCTCTATCTTCCCTTGGTCCGTG TTTCAAGA 3′

Amplicons were cleaned and quantified using a Qbit Fluorometer (Thermo Fisher Scientific, Waltham, MA, USA) before barcoding and library preparation which was undertaken in accordance with ONT protocol: Ligation sequencing amplicons—PCR barcoding (SQK-LSK110 with EXP-PBC096); in brief, the following was undertaken.

A secondary 50 µL barcoding PCR was performed on 100–200 fmol of the cleaned primary PCR product using EXP-PBC096 barcoding primers (noting which barcode is used for each sample) and 15 cycles of the following thermal regime: 95 °C for 15 s, 62 °C for 15 s, 65 °C for 90 s and a final extension of 65 °C for 10 min. The resulting amplicons were cleaned using mag beads and quantified using a Qbit fluorometer. These barcoded and cleaned amplicons were pooled to create a mixture containing a total of 1 µg of DNA with each constituent sample diluted so equally represented. The pooled amplicons had DNA control standards attached, had ends repaired and underwent formalin-fixed paraffin-embedded (FFPE) repair using the relevant reagents from the NEBNext companion module for ligation sequencing, at 20 °C for 5 min followed by 65 °C for 5 min. The repaired and end prepped library then had adapter sequences added using the NEBNext T4 ligase from the companion module, this required a 10 min incubation at room temperature prior to one last mag bead clean, eluting into 15 µL of molecular grade water. An amount of 1 µL of the preparation was quantified and 5–10 fmol of this sample was suspended in sequencing buffer and loading beads before being loaded on an R9.4.1 flow cell (FLO-MIN106) mounted on a MinION MK1c (ONT, Oxford, UK). Sequencing was allowed to continue till all pores had been exhausted; this required reloading of the library when necessary.

In total, 59 water samples were sequenced over three libraries, each run on different flow cells so as to generate adequate volumes of data.

#### 5.4.2. Bioinformatic Analysis of Molecular Data

##### Base Calling

Preliminary base calling during sequencing was performed on the MinION MK1c, using Guppy v5.0.11 using the “fast” sequencing model to facilitate the tracking of sequencing performance. For onward analysis a desktop computer running Linux Ubuntu (Kernel Linux 5.4.0126-generic x86_64), with a 20 core CPU (Intel Xeon W-2255 CPU), NVIDIA Quatro GV100 GPU (5120 cores, 32 gb memory) was used for both super high accuracy base calling (Q8 filtered) and data processing. The super high accuracy base calling used Guppy v5.0.11 and the included ‘dna_r9.4_450bps_sup.cfg’ configuration file.

##### Alignment to Reference Sequences

Raw reads in FASTQ format were aligned against 18 S rDNA sequences from version 4.13.0 of the PR2 database using version 2.20 of the minimap2 alignment software [95,151,152,153]. BamTools was used to filter results so that only alignments which had a dynamic programming score less than 2800 and a gap-compressed per-base sequence divergence of less than 0.05 were removed [151]. Following filtering of alignment results, coverage against each reference sequence was summarized using the coverage command in SAMtools version 1.12 [152]. Aligned reads were tallied for each genus on each date, with species associated with HAB events being tabulated for comparison with data generated from light microscopy. To provide an overview of temporal changes, the data was grouped by month and displayed using Krona, to provide an interactive metagenomic viewer (Supplementary Materials S1).

##### Generation of Consensus Sequences

Sequences aligning to genera of interest on the PR2 database were extracted to generate consensus sequences. This was either performed on individual samples or, if insufficient data was available, multiple samples were combined.

To minimize bias from the PR2 reference list, a de novo approach was adopted to generate consensus sequences. NGSpeciesID software (version 0.1.1.1) was used to carry out reference-free clustering and subsequent generation of consensus sequences for each sample in the sequencing run [96]. The average and maximum allowed amplicon length and deviation were set to 3000 and 300, respectively. The “mapped”, “aligned” and “reverse complement” thresholds were set as a default to 0.7, 0.5 and 0.8, respectively, but on occasion required ad hoc adjustment). Following clustering of reads, spoa (version 4.0.7) was used to determine the consensus sequence of reads in each cluster, with medaka (version 1.2.4) and polishing completed using r941_min_high_g360 model [154,155]. Integrative Genomics Viewer (IGV) was used to visually assess the reads used to generate the consensus sequence for anomalies such as hybridized sequences or low coverage areas.

##### Phylogenetic Analysis of Consensus Sequences

Consensus sequences that had aligned with genera of interest were aligned with reference sequences either using MAFFT v7.480, which was run using default parameters, except the direction of sequences in the input file was automatically adjusted, if necessary. Poorly conserved regions were trimmed from the 5′ and 3′ end of the alignment using UGENE v37.0. IQ-tree version 2.1.2 was used to infer phylogeny, using a maximum likelihood general time reversible model with empirical base frequencies as well as a discrete gamma model with four rate categories, with the model picked using the Bayesian Information Criterion. FigTree version 1.4.4 was then used to plot the phylogenetic tree and adjust labels. Trees were visualized using the Interactive Tree of Life online tool [156] and adjustments were made where necessary using Inkscape (version 1.2).

### 5.5. Shellfish Toxin Analysis

#### 5.5.1. Chemical Assays

Lipophilic toxin (LT) analysis was conducted following the methods of [63,64], using a Waters Xevo TQ-S triple quadrupole mass spectrometer equipped with ESI probe and operating in Multiple Reaction Monitoring Mode, coupled with an Acquity UPLC (Waters, Manchester, UK). Chromatography was conducted using a Waters BEH C18 1.7 µm column (50 × 2.1 mm) equipped with matching VanGuard and pre-filter, and held at 30 °C. Chromatographic mobile phases consisted of deionized water for Mobile Phase A and 90% aqueous acetonitrile for MP B, both containing 0.1% ammonium hydroxide solution, with a UPLC gradient from 15 to 100% B performed over three minutes, and a sample injection volume of 5 µL. For emerging lipophilic toxins, a methanolic 1 mM ammonium fluoride mobile phase was used, along with MS source conditions as described in [157]. Individual MRM transitions and parameters are described in Table S3 and were optimized prior to analysis under flow conditions. Data acquisition and analysis was performed using MassLynx and TargetLynx v4.2.

Analysis for hydrophilic marine toxins was conducted using HILIC-MS/MS of desalted aqueous fractions based on the method of [69]. Following carbon SPE clean-up using Supelclean ENVI-Carb 250 mg/3 mL SPE cartridges and dilution of SPE eluants in acetonitrile (1:3 *v*/*v*), samples were analysed for paralytic shellfish toxins (PST) and tetrodotoxin (TTX). Chromatographic separation was performed using an Agilent 1290 Infinity II UHPLC with a 20 µL injection loop. An Agilent 6495 B triple quadrupole LC/MS with the iFunnel and Jet Stream technology was utilized as the detector. The analysis was performed using fast polarity switching mode with electrospray ionization. Data acquisition was performed using Agilent MassHunter Acquisition software (Version B.08.00), while data processing was performed using Agilent MassHunter Quantitative and Qualitative Analysis software (Version 10.0). MS/MS acquisition methods were set up using the source conditions described by [69] and specific MRM transitions summarized in Supplementary Table S3. Positive mode (ESI+) transitions were used exclusively for STX, NEO, dcSTX, dcNEO, doSTX, and TTX. Negative mode (ESI−) transitions were used exclusively for GTX1, GTX2, dcGTX2, dcGTX1, and C1 (α-epimers). For the remaining analogs (GTX3, GTX4, GTX5, GTX6, dcGTX3, dcGTX4, C2, C3, and C4) a mix of positive and negative MRMs were used.

Analysis for ASP was conducted using HPLC with UV detection as described by [59], following the method of [58]. An Agilent LC-UV 1200 system was used comprising a quaternary pump, vacuum degasser, autosampler, column oven and UV-diode detector, monitoring at a wavelength of 242 nm.

Limits of Quantitation (LOQ) for each toxin were determined previously in the laboratory, and as summarized in Table 5. These were typically lower than the Reporting Limits utilized for Official Control testing but are here calculated based on a signal-to-noise ratio of 10:1 for primary target peaks for the main compounds of interest.

#### 5.5.2. Immunoassays

The DSP, ASP and PSP Toxin Alert LFAs produced by Sensoreal (Montreal, QC, Canada) were utilized for rapid screening of shellfish sample extracts for each of the three regulated groups of shellfish toxins. Protocols supplied by the manufacturer were followed as closely as possible for the testing, with modifications made to the buffer dilutions to account for differences in extraction conditions between regulatory extraction protocols and test kit extraction protocols, given that only shellfish tissue solvent extracts were available in storage for LFA testing. Each test involved the dilution of the shellfish solvent extract with a diluted buffer provided in the kits, prior to aliquoting 70 µL of the diluted sample into a vial containing conjugate material. The same approach was followed for DSP, except that methanolic extracts of shellfish were hydrolyzed to liberate acyl esters, prior to buffer dilution. After incubation for a set period of time (typically 10 min), the solution was pipetted onto the LFA strip and allowed to elute, prior to reading the intensities of test (T) and control (C) lines on the Sensoreader scanner provided with the kits. The output from the scanners were recorded as calculated from the T/C ratios, giving final results in terms of ‘Low’, ‘Medium’ and ‘High’.

## Figures and Tables

**Table 1:** Mean monthly cell densities (cells/L) ± standard deviations across all sampling sites for each HAB genus/species determined by laboratory light microscopy of seawater samples. * Molecular data indicates that cells identified as *P. cordatum/balticum* are potentially the newly discovered species *Prorocentrum spinulentum*.

Genus/Species	March	April	May	June	July	August	September	October
*Vulcanodinium* sp.	nd	nd	nd	nd	1800	nd	400	nd
*Alexandrium* sp.	nd	nd	150 ± 70	100 ± 0	125 ± 50	400	nd	nd
*Amphidinium cartarae*	nd	nd	nd	nd	100	nd	nd	nd
*Dinophysis* sp.	nd	100	100 ± 0	150 ± 70	nd	250 ± 212	nd	nd
*Dinophysis acuminata*	nd	100	133 ± 58	300 ± 245	750 ± 712	467 ± 305	100	200
*Gymnodinium* sp.	nd	nd	nd	nd	100	nd	nd	nd
*Heterocapsa minima*/*Azadinium/Amphidoma* group	nd	nd	nd	nd	21,000	48,600	7,201,000	nd
Indet. dinoflagellate	nd	nd	nd	nd	100	nd	nd	nd
*Karenia* sp.	nd	nd	nd	100 ± 0	200	400	nd	nd
Prorocentrum cordatum/balticum *	nd	nd	nd	nd	466 ± 306	400	600	nd
*Prorocentrum lima*	nd	300	100	100 ± 0	nd	1200 ± 1414	100	nd
Prymnesiophytes	nd	nd	nd	nd	nd	100	nd	nd
*Pseudo-Nitzschia delicatissima* group (≤4.9 µm)	nd	nd	nd	4800	1133 ± 702	800	nd	nd
*Pseudo-nitzschia multistriata*	nd	nd	nd	1200	267 ± 115	600	nd	nd
*Pseudo-Nitzschia seriata* group (≥5 µm)	nd	nd	nd	1200	600 ± 673	2000	400	nd

**Table 2:** List of consensus sequences generated from reads aligning with HAB genera throughout the study.

Genus	Classification	Sequence Number	Region Analysed	Taxonomic Name	% Ident	Accession	Harmful Effect/Toxin Hazard
*Alexandrium*	*Dinoflagellata*	1	SSU—LSU	*Alexandrium tamarense*	99.70%	DQ785891.1	Non-toxic species
			LSU	*Alexandrium tamarense*	99.82%	KX599342.1	
*Azadinium*	*Dinoflagellata*		SSU	*Azadinium caudatum var. margalefii*	99.60%	JQ247707.1	Non-toxic species
		2	ITS1-ITS2	*Azadinium caudatum var. margalefii*	99.43%	JQ247705.1	
			LSU	*Azadinium caudatum*	99.34%	JQ247709.1	
*Coolia*	*Dinoflagellata*		SSU—LSU	*Coolia monotis*	99.08%	AJ415509.1	Neurotoxic/Cooliatoxin
		3	ITS1-ITS2	*Coolia monotis*	98.94%	KJ781411.2	
			SSU	*Coolia monotis*	99.25%	AJ415509.1	
*Dictyochophyceae*	*Dictyocha*	4a	SSU	*Dictyocha speculum*	99.83%	U14385.1	Gill damage/not toxic
			ITS1-LSU	*Dictyocha speculum*	99.80%	AF289046.1	
	*Aureococcus*	4b	SSU	*Aureococcus anophagefferens*	99.50%	AF117776.1	Blocks light penetration/not toxic
			LSU	*Aureococcus anophagefferens*	98.34%	AF289042.1	
*Dinophysis*	*Dinoflagellata*	5	SSU—LSU	*Dinophysis acuminata*	99.77%	MK860896.1	Diahretic/Okadaic acid, Dinophysistoxins and pectenotoxins
*Karlodinium*	*Dinoflagellata*	6	ITS1-ITS2	*Karlodinium decipiens*	97.48%	LC521287.1	Non-toxic species
			LSU	*Karlodinium antarcticum*	100.00%	EF469234.1	No data available
*Karenia*	*Dinoflagellata*	7	SSU—LSU	*Karenia mikimotoi*	99.11%	KU314866.1	Ichthyotoxic/Gymnocins and Bevatoxins
*Noctiluca*	*Dinoflagellata*	8	SSU—LSU	*Noctiluca scintillans*	99.63%	OQ132786.1	Oxygen depletion/not toxic
			ITS1-ITS2	*Noctiluca scintillans*	99.36%	KR607082.1	
*Prorocentrum*	*Dinoflagellata*		SSU	*Prorocentrum lima*	99.82%	MK541784.1	Diahretic/Okadaic acid and Dinophysis toxin-1
		10a	ITS1-ITS2	*Prorocentrum lima*	99.68%	AB189765.1	
			LSU	*Prorocentrum lima*	99.85%	MW177927.1	
			SSU	*Prorocentrum spinulentum*	100.00%	OQ220501.1	No data
		10b	ITS1-ITS2	*Prorocentrum spinulentum*	100.00%	OQ220500.1	
			LSU	*Prorocentrum spinulentum*	99.85%	OP231463.1	
*Pseudo-nitzschia*		11a	ITS2	*Pseudo-nitzschia plurisecta*	100.00%	MK729547.1	Amnesic/Domoic acid
	*Stramenopiles*	11b	ITS2	*Pseudo-nitzschia delicatissima*	100.00%	KM245508.1	
	*Ochrophytina*	11c	ITS2	*Pseudo-nitzschia fraudulenta*	100.00%	MK106639.1	
		11d	ITS2	*Pseudo-nitzschia australis*	100.00%	JN599166.1	

Sequences available in Supplementary Materials Data S1 ; SSU = Small Subunit Ribosomal RNA; ITS = Internal Transcribed Spacer Ribosomal RNA; LSU = Large Subunit Ribosomal RNA.

**Table 3:** Marine toxin concentrations quantified in bivalve shellfish collected from IoS between March 2020 and July 2021.

Date Collected	Sample Number	Species	Island	Location	DA	PST	TTX	OA/DTXs	AZAs	CIs
9 March 2020	20	Co	St. Mary’s	Old Town Bay	nd	3.5	nd	nd	1.1	nd
9 March 2020	21a	Co	St. Mary’s	Porth Hellick	nd	nd	nd	4.6	2.6	0.2
9 March 2020	21b	SC	St. Mary’s	Porth Hellick	nd	nd	nd	nd	nd	nd
10 March 2020	33	Co	Tresco	Tresco/Bryher	nd	nd	nd	nd	2.2	0.4
10 March 2020	49	Co	Tresco	Raven’s Porth	nd	nd	nd	nd	2.3	0.6
10 March 2020	50	SC	Tresco	Raven’s Porth	nd	3.2	nd	nd	nd	nd
11 March 2020	47	SC	Tresco	English Island Point	nd	nd	nd	nd	1	nd
11 March 2020	48	Rz	St. Martin’s	St. Martin’s Flats	nd	nd	nd	4.6	nd	nd
11 March 2020	51	Co	St. Martin’s	St. Martin’s Flats	nd	nd	nd	nd	2.2	nd
13 March 2020	45	Co	Bryher	Hangman’s Point	nd	nd	nd	nd	3.2	0.1
13 March 2020	46	Co	Bryher	Green Bay Quay	nd	nd	nd	nd	2.4	0.4
1 April 2020	52	Co	St. Mary’s	Porth Hellick	nd	nd	nd	nd	3.2	nd
12 April 2020	55	Co	St. Mary’s	Porth Hellick	nd	nd	nd	3.9	6.5	nd
30 April 2020	56	Co	St. Mary’s	Porth Hellick	nd	nd	nd	2.6	3.4	2
30 April 2020	61	CC	St. Mary’s	Porth Hellick	nd	nd	nd	12	1.7	nd
15 May 2020	62	CC	St. Mary’s	Deep Point	nd	nd	nd	28	8.4	nd
24 May 2020	59	M	St. Mary’s	Deep Point	1.18	nd	nd	7.4	6.2	nd
3 June 2020	63	CC	St. Mary’s	Old Town	nd	nd	nd	9.6	4.2	nd
10 June 2020	64	Co	St. Mary’s	Porth Hellick	nd	4.1	6.88	16.0	5.1	nd
15 June 2020	65	Co	St. Mary’s	Bar Point	nd	nd	25.2	21.2	2.3	nd
21 June 2020	53	Co	St. Mary’s	Porth Hellick	nd	nd	16.88	11.8	3.6	nd
28 June 2020	54	Co	St. Mary’s	Porth Hellick	nd	nd	3.77	10.6	2	2.9
4 July 2020	66	Co	St. Mary’s	Porthmellon	nd	3.6	6.52	34.2	nd	nd
10 July 2020	67	CC	St. Mary’s	Little Porth	nd	nd	nd	42.3	4.2	nd
18 July 2020	68	CC	St. Mary’s	Porth Hellick	nd	nd	2.45	16.9	6.3	nd
28 July 2020	69	Co	St. Mary’s	Deep Point	nd	nd	nd	24.3	12.3	nd
9 August 2020	57	Co	St. Mary’s	Porth Hellick	nd	nd	0.53	75	2.8	nd
9 August 2020	58	M	St. Mary’s	Deep Point	1.29	nd	nd	4.9	nd	nd
20 August 2020	70	Co	St. Mary’s	Deep Point	nd	nd	1.13	24.3	11.7	nd
1 September 2020	71	Co	St. Mary’s	Porth Hellick	nd	nd	2.13	12.3	4.5	nd
7 September 2020	72	Co	St. Mary’s	Deep Point	nd	nd	nd	8.2	3.2	nd
29 November 2020	128	Co	St Mary’s	Porth Hellick	0.57	nd	nd	14.6	9	nd
29 November 2020	129	Co	St Mary’s	Old Town	nd	nd	nd	nd	8.4	nd
20 December 2020	130	Co	St Mary’s	Porth Hellick	0.42	nd	nd	10.3	9	nd
20 December 2020	131	Co	St Mary’s	Old Town	0.37	nd	nd	nd	7.8	nd
3 January 2021	132	Co	St Mary’s	Porth Hellick	0.62	nd	nd	10.1	8.2	nd
3 January 2021	133	Co	St Mary’s	Old Town	nd	nd	nd	nd	9.9	nd
17 January 2021	134	Co	St Mary’s	Porth Hellick	nd	nd	nd	7.8	8.6	nd
17 January 2021	135	Co	St Mary’s	Old Town	0.34	nd	nd	nd	4.1	nd
31 January 2021	136	Co	St Mary’s	Porth Hellick	nd	nd	nd	13.2	3.6	nd
31 January 2021	137	Co	St Mary’s	Old Town	nd	nd	nd	nd	4.3	nd
8 February 2021	138	Co	St Mary’s	Porth Hellick	0.35	nd	nd	nd	4	nd
28 February 2021	139	Co	St Mary’s	Old Town	nd	nd	nd	15.1	9.5	nd
28 February 2021	140	Co	St Mary’s	Porth Hellick	nd	nd	nd	nd	8.8	nd
2 March 2021	141	Co	St Mary’s	Old Town	nd	nd	nd	11	10.6	nd
13 March 2021	142	Co	St Mary’s	Porth Hellick	nd	nd	nd	nd	9.3	nd
4 April 2021	143	Co	St Mary’s	Old Town	nd	nd	nd	12.2	8.6	2.4
13 April 2021	144	Co	St Mary’s	Porth Hellick	0.56	nd	nd	7.3	8.8	nd
25 April 2021	145	Co	St Mary’s	Old Town	0.32	nd	nd	nd	9.2	nd
7 May 2021	146	Co	St Mary’s	Porth Hellick	nd	nd	nd	nd	3.8	nd
16 May 2021	147	Co	St Mary’s	Porth Hellick	nd	nd	nd	nd	3.8	nd
1 June 2021	148	Co	St Mary’s	Old Town	nd	nd	3.77	12.5	8.6	nd
10 June 2021	149	Co	St Mary’s	Porth Hellick	nd	nd	8.46	30.1	8.6	nd
21 June 2021	150	Co	St Mary’s	Porth Hellick	nd	nd	12.3	14.3	4.3	nd
29 June 2021	151	Co	St Mary’s	Old Town	nd	nd	2.46	8.6	8.3	nd
8 July 2021	152	Co	St Mary’s	Porth Hellick	nd	nd	nd	4.3	8.5	nd
14 July 2021	153	Co	St Mary’s	Old Town	nd	nd	nd	nd	2.1	nd

Species listed as Co = cockles; CC = grooved carpet clams; M = mussels; Rz = razor clams; SC = surf clams; DA = domoic acid (mg/kg); PST = sum of paralytic shellfish toxins (µg STX di-HCl eq/kg); TTX = Tetrodotoxins (µg/kg); OA/DTXs = sum of free and esterified okadaic acid group toxins (µg/kg), including PTXs, but none detected in any study samples; AZAs = sum of azaspiracids (µg/kg); CIs = sum of cyclic imines, including spirolides and pinnatoxins (µg/kg); nd = not detected.

**Table 4:** Summary of Sensoreal Alert LFA results obtained on selected bivalve samples (*n* = 20) when compared against DA concentrations (mg/kg), total PST concentrations (µg STX eq/kg) and total OA group toxicity (µg OA eq/kg).

Sample	Species	DA		PST		OA/DTXs	
		Lab	LFA	Lab	LFA	Lab	LFA
20	Co	nd	Low	3.5	Low	nd	Low
50	SC	nd	Low	3.2	Low	nd	Low
48	Rz	nd	Low	nd	Low	4.6	Low
61	CC	nd	Low	nd	Low	12	Low
62	CC	nd	Low	nd	Low	28	Low
64	Co	nd	Low	4.1	Low	16.0	Low
65	Co	nd	Low	nd	Low	21.2	Low
66	Co	nd	Low	3.6	Low	34.2	Medium
67	CC	nd	Low	nd	Low	42.3	Medium
68	CC	nd	Low	nd	Low	16.9	Low
69	Co	nd	Low	nd	Low	24.3	Low
57	Co	nd	Low	nd	Low	75	Medium
58	M	1.29	Low	nd	Low	4.9	Low
70	Co	nd	Low	nd	Low	24.3	Low
132	Co	0.62	Low	nd	Low	10.1	Low
139	Co	nd	Low	nd	Low	15.1	Low
141	Co	nd	Low	nd	Low	11	Low
143	Co	nd	Low	nd	Low	12.2	Low
149	Co	nd	Low	nd	Low	30.1	Low
150	Co	nd	Low	nd	Low	14.3	Low

nd = not detected.

**Table 5:** Summary of LOQs for toxins quantified in this study.

Analyte	LOD	Method	Reference
DA	0.2 mg/kg	LC-UV	[59]
LTs (OA, DTXs, AZAs)	0.5 µg/kg	UPLC-MS/MS	[63,64]
YTXs	4.2 µg/kg	UPLC-MS/MS	[64]
Cyclic imines	0.1 µg/kg	UPLC-MS/MS	[157]
STX, GTX5, C1&2, GTX6	0.5 µg STX di-HCl eq/kg	HILIC-MS/MS	[69]
dcNEO, dcSTX	1.0 µg STX di-HCl eq/kg	HILIC-MS/MS	[69]
dcGTX2&3, C3&4	2.4 µg STX di-HCl eq/kg	HILIC-MS/MS	[69]
GTX1-4	2.7 µg STX di-HCl eq/kg	HILIC-MS/MS	[69]

## Data Availability

The original contributions presented in this study are included in the article/Supplementary Materials. Further inquiries can be directed to the corresponding author.

## Supplementary Materials

The following supporting information can be downloaded at: https://www.mdpi.com/article/10.3390/md23120478/s1, Figure S1. Krona Interactive metagenomic viewer, displaying data grouped by month and combined; Figure S2. Maximum likelihood inferred tree of *Alexandrium* based on sequences of LSU using 10,000 Bootstraps and BS scores of <70 not shown, the branch lengths are shown in scale; Figure: S3. Maximum likelihood inferred tree of *Azadinium* and *Amphidoma* based on sequences of internal transcribe spacer (ITS1-ITS2) using 10,000 Bootstraps and BS scores of <70 not shown and branch lengths are in scale; Figure S4. Maximum likelihood inferred tree of *Prorocentrum*, using internal transcribed spacer regions, using 10,000 Bootstraps and score <70 not shown and the branch lengths are in scale; Figure S5. Maximum likelihood inferred tree of Coolia based on sequences of internal transcribe spacers using 10,000 Bootstraps, with scores of <70 not shown and branch lengths are in scale; Figure S6. Maximum likelihood inferred tree of *Kareniacea*, including sequences aligning with *Karenia* and *Karlodinium* based on sequences of LSU using 10,000 Bootstraps, with scores of <70 not shown and branch lengths shown in scale; Figure S7. Maximum likelihood inferred tree of *Kareniacea*, including sequences aligning with *Karenia* and *Karlodinium* based on sequences of ITS using 10,000 Bootstraps scores of <70 not shown, and branch lengths shown in scale; Figure S8. Maximum likelihood inferred tree of Dictyochyceans, including sequences aligning with Dictyocha and Aureococcus based on sequences of small subunit (SSU/18 S) using 10,000 Bootstraps with scores of <70 not shown, and branch lengths shown in scale; Figure S9. Negative Binomial Generalized Linear model output for the effect of Region, Time and Species on cell counts. *Pseudo nitzschia* and Mainland are set to the references; Figure S10. Binomial Generalized Linear model outputs for the effect of region on concentrations of PSTs, DA and Total OA group; Figure S11. Figures showing toxin profiles across all shellfish samples in terms of (a) µg/L for OA, DTX1, AZA1 and AZA2 (b) proportions of OA and DTX1 in terms of OA eq/kg and (c) proportions of AZA1 eq/kg for AZA1 and AZA2; Data S1 Consensus sequences generated in the study in fasta format; Table S1: Summary of sampling sites utilized during study; Table S2. Summary of HABs detected and quantified (cell densities in cells/L) by laboratory light microscopy across all sampling sites from the Isles of Scilly, March to October 2020; Table S3: List of MRM transitions for targeted analysis of each method highlighting primary (1°) and secondary transitions (2°) and associated collision energies (CE) and cone voltages (CV).
