# Supplementary material for: ScillyHAB: A Multi-Disciplinary Survey of Harmful Marine Phytoplankton and Shellfish Toxins in the Isles of Scilly: Combining Citizen Science with State-of-the-Art Monitoring in an Isolated UK Island Territory
Source: Mar Drugs. 2025 Dec 15;23(12):478. doi: 10.3390/md23120478 (PMC12734806; doi:10.3390/md23120478)
Supplement: Supplementary file 1 [file marinedrugs-23-00478-s001.zip › marinedrugs-4017030-supplementary/Figure_S1_Interative_metagenomic_viewer.html]

Javascript must be enabled to view this page.

magnitude

March
April
May
June
July
August
September
Total

 237808
 26222
 107263
 64108
 271959
 193433
 262573
 1163859

 11115
 1624
 713
 3756
 7023
 5308
 2205
 32237

 238
 679
 0
 73
 6723
 5210
 457
 13380

 235
 679
 0
 73
 6723
 5210
 457
 13377

 223
 679
 0
 73
 6723
 5210
 439
 13347

 44
 0
 0
 0
 3
 0
 405
 452

 44
 0
 0
 0
 3
 0
 405
 452

 121
 558
 0
 72
 6697
 13
 20
 7481

 10
 0
 0
 0
 0
 0
 0
 10

 52
 558
 0
 72
 6697
 13
 20
 7412

 59
 0
 0
 0
 0
 0
 0
 59

 58
 121
 0
 1
 23
 5197
 14
 5414

 58
 121
 0
 1
 23
 5197
 14
 5414

 12
 0
 0
 0
 0
 0
 18
 30

 12
 0
 0
 0
 0
 0
 18
 30

 12
 0
 0
 0
 0
 0
 18
 30

 3
 0
 0
 0
 0
 0
 0
 3

 3
 0
 0
 0
 0
 0
 0
 3

 3
 0
 0
 0
 0
 0
 0
 3

 3
 0
 0
 0
 0
 0
 0
 3

 5267
 793
 699
 3466
 290
 2
 879
 11889

 3726
 264
 306
 413
 0
 0
 0
 5202

 3726
 264
 306
 413
 0
 0
 0
 5202

 3669
 264
 42
 413
 0
 0
 0
 4881

 5
 0
 5
 8
 0
 0
 0
 18

 475
 0
 1
 23
 0
 0
 0
 499

 6
 0
 0
 3
 0
 0
 0
 9

 163
 0
 0
 0
 0
 0
 0
 163

 34
 0
 0
 0
 0
 0
 0
 34

 55
 151
 0
 0
 0
 0
 0
 206

 52
 0
 0
 0
 0
 0
 0
 52

 9
 0
 28
 64
 0
 0
 0
 101

 2
 0
 0
 0
 0
 0
 0
 2

 13
 0
 0
 43
 0
 0
 0
 56

 1
 0
 0
 0
 0
 0
 0
 1

 1
 0
 0
 9
 0
 0
 0
 10

 2
 0
 0
 0
 0
 0
 0
 2

 410
 103
 0
 1
 0
 0
 0
 514

 39
 0
 6
 137
 0
 0
 0
 182

 27
 0
 0
 0
 0
 0
 0
 27

 1410
 0
 0
 0
 0
 0
 0
 1410

 11
 10
 0
 0
 0
 0
 0
 21

 457
 0
 0
 0
 0
 0
 0
 457

 4
 0
 0
 13
 0
 0
 0
 17

 493
 0
 0
 0
 0
 0
 0
 986

 0
 0
 1
 3
 0
 0
 0
 4

 0
 0
 1
 0
 0
 0
 0
 1

 0
 0
 0
 88
 0
 0
 0
 88

 0
 0
 0
 20
 0
 0
 0
 20

 0
 0
 0
 1
 0
 0
 0
 1

 49
 0
 264
 0
 0
 0
 0
 313

 6
 0
 0
 0
 0
 0
 0
 6

 1
 0
 2
 0
 0
 0
 0
 3

 1
 0
 0
 0
 0
 0
 0
 1

 1
 0
 0
 0
 0
 0
 0
 1

 3
 0
 0
 0
 0
 0
 0
 3

 5
 0
 0
 0
 0
 0
 0
 5

 1
 0
 0
 0
 0
 0
 0
 1

 2
 0
 0
 0
 0
 0
 0
 2

 7
 0
 137
 0
 0
 0
 0
 144

 15
 0
 0
 0
 0
 0
 0
 15

 6
 0
 124
 0
 0
 0
 0
 130

 1
 0
 0
 0
 0
 0
 0
 1

 0
 0
 1
 0
 0
 0
 0
 1

 8
 0
 0
 0
 0
 0
 0
 8

 1
 0
 0
 0
 0
 0
 0
 1

 7
 0
 0
 0
 0
 0
 0
 7

 593
 0
 59
 0
 0
 1
 189
 842

 593
 0
 59
 0
 0
 1
 189
 842

 593
 0
 59
 0
 0
 1
 189
 842

 2
 0
 0
 0
 0
 0
 0
 2

 383
 0
 59
 0
 0
 0
 0
 442

 37
 0
 0
 0
 0
 0
 0
 37

 2
 0
 0
 0
 0
 0
 0
 2

 18
 0
 0
 0
 0
 0
 0
 18

 33
 0
 0
 0
 0
 0
 0
 33

 1
 0
 0
 0
 0
 0
 0
 1

 6
 0
 0
 0
 0
 0
 0
 6

 27
 0
 0
 0
 0
 0
 0
 27

 9
 0
 0
 0
 0
 0
 0
 9

 1
 0
 0
 0
 0
 0
 0
 1

 7
 0
 0
 0
 0
 0
 0
 7

 22
 0
 0
 0
 0
 0
 0
 22

 1
 0
 0
 0
 0
 0
 0
 1

 3
 0
 0
 0
 0
 0
 0
 3

 1
 0
 0
 0
 0
 0
 0
 1

 3
 0
 0
 0
 0
 0
 0
 3

 1
 0
 0
 0
 0
 0
 0
 1

 27
 0
 0
 0
 0
 0
 0
 27

 7
 0
 0
 0
 0
 0
 7
 14

 2
 0
 0
 0
 0
 0
 0
 2

 0
 0
 0
 0
 0
 1
 159
 160

 0
 0
 0
 0
 0
 0
 9
 9

 0
 0
 0
 0
 0
 0
 14
 14

 18
 0
 0
 6
 0
 0
 0
 24

 18
 0
 0
 6
 0
 0
 0
 24

 15
 0
 0
 0
 0
 0
 0
 15

 2
 0
 0
 0
 0
 0
 0
 2

 1
 0
 0
 0
 0
 0
 0
 1

 2
 0
 0
 0
 0
 0
 0
 2

 2
 0
 0
 0
 0
 0
 0
 2

 2
 0
 0
 0
 0
 0
 0
 2

 2
 0
 0
 0
 0
 0
 0
 2

 4
 0
 0
 0
 0
 0
 0
 4

 3
 0
 0
 6
 0
 0
 0
 9

 1
 0
 0
 0
 0
 0
 0
 1

 1
 0
 0
 0
 0
 0
 0
 1

 1
 0
 0
 0
 0
 0
 0
 1

 0
 0
 0
 6
 0
 0
 0
 6

 312
 3
 305
 146
 23
 0
 3
 792

 312
 3
 305
 146
 23
 0
 3
 792

 312
 3
 305
 146
 23
 0
 3
 792

 312
 3
 305
 146
 23
 0
 3
 792

 17
 0
 0
 0
 0
 0
 0
 17

 17
 0
 0
 0
 0
 0
 0
 17

 17
 0
 0
 0
 0
 0
 0
 17

 16
 0
 0
 0
 0
 0
 0
 16

 1
 0
 0
 0
 0
 0
 0
 1

 15
 0
 1
 0
 1
 0
 23
 40

 12
 0
 1
 0
 1
 0
 23
 37

 7
 0
 1
 0
 1
 0
 23
 32

 4
 0
 1
 0
 0
 0
 21
 26

 2
 0
 0
 0
 0
 0
 0
 2

 1
 0
 0
 0
 1
 0
 2
 4

 4
 0
 0
 0
 0
 0
 0
 4

 4
 0
 0
 0
 0
 0
 0
 4

 1
 0
 0
 0
 0
 0
 0
 1

 1
 0
 0
 0
 0
 0
 0
 1

 3
 0
 0
 0
 0
 0
 0
 3

 3
 0
 0
 0
 0
 0
 0
 3

 1
 0
 0
 0
 0
 0
 0
 1

 2
 0
 0
 0
 0
 0
 0
 2

 428
 0
 0
 0
 0
 0
 0
 428

 428
 0
 0
 0
 0
 0
 0
 428

 428
 0
 0
 0
 0
 0
 0
 428

 1
 0
 0
 0
 0
 0
 0
 1

 427
 0
 0
 0
 0
 0
 0
 427

 1
 0
 0
 0
 0
 0
 0
 1

 1
 0
 0
 0
 0
 0
 0
 1

 1
 0
 0
 0
 0
 0
 0
 1

 1
 0
 0
 0
 0
 0
 0
 1

 93
 525
 27
 306
 265
 1
 664
 1881

 86
 0
 0
 0
 49
 0
 1
 136

 86
 0
 0
 0
 49
 0
 1
 136

 86
 0
 0
 0
 0
 0
 0
 86

 0
 0
 0
 0
 49
 0
 1
 50

 7
 525
 27
 306
 216
 1
 663
 1745

 2
 0
 0
 0
 0
 0
 0
 2

 1
 0
 0
 0
 0
 0
 0
 1

 1
 0
 0
 0
 0
 0
 0
 1

 5
 525
 27
 306
 216
 1
 663
 1743

 2
 0
 0
 0
 0
 0
 0
 2

 2
 0
 0
 1
 214
 0
 0
 217

 1
 524
 7
 22
 0
 0
 0
 554

 0
 1
 20
 0
 0
 0
 0
 21

 0
 0
 0
 283
 0
 0
 0
 283

 0
 0
 0
 0
 2
 1
 614
 617

 0
 0
 0
 0
 0
 0
 2
 2

 0
 0
 0
 0
 0
 0
 29
 29

 0
 0
 0
 0
 0
 0
 1
 1

 0
 0
 0
 0
 0
 0
 17
 17

 57
 1
 1
 2595
 1
 0
 0
 2655

 57
 1
 1
 2595
 1
 0
 0
 2655

 57
 1
 1
 2595
 1
 0
 0
 2655

 56
 0
 0
 0
 0
 0
 0
 56

 1
 0
 0
 0
 0
 0
 0
 1

 0
 1
 1
 2574
 1
 0
 0
 2577

 0
 0
 0
 8
 0
 0
 0
 8

 0
 0
 0
 13
 0
 0
 0
 13

 2
 0
 0
 0
 0
 0
 0
 2

 2
 0
 0
 0
 0
 0
 0
 2

 2
 0
 0
 0
 0
 0
 0
 2

 1
 0
 0
 0
 0
 0
 0
 1

 1
 0
 0
 0
 0
 0
 0
 1

 4
 0
 0
 0
 0
 0
 0
 4

 4
 0
 0
 0
 0
 0
 0
 4

 4
 0
 0
 0
 0
 0
 0
 4

 3
 0
 0
 0
 0
 0
 0
 3

 1
 0
 0
 0
 0
 0
 0
 1

 1
 0
 0
 0
 0
 0
 0
 1

 1
 0
 0
 0
 0
 0
 0
 1

 1
 0
 0
 0
 0
 0
 0
 1

 1
 0
 0
 0
 0
 0
 0
 1

 5187
 151
 14
 217
 6
 1
 867
 6443

 2663
 98
 2
 206
 0
 0
 0
 2969

 2554
 0
 0
 0
 0
 0
 0
 2554

 1745
 0
 0
 0
 0
 0
 0
 1745

 16
 0
 0
 0
 0
 0
 0
 16

 1681
 0
 0
 0
 0
 0
 0
 1681

 1
 0
 0
 0
 0
 0
 0
 1

 5
 0
 0
 0
 0
 0
 0
 5

 42
 0
 0
 0
 0
 0
 0
 42

 809
 0
 0
 0
 0
 0
 0
 809

 56
 0
 0
 0
 0
 0
 0
 56

 3
 0
 0
 0
 0
 0
 0
 3

 746
 0
 0
 0
 0
 0
 0
 746

 1
 0
 0
 0
 0
 0
 0
 1

 1
 0
 0
 0
 0
 0
 0
 1

 2
 0
 0
 0
 0
 0
 0
 2

 103
 0
 0
 0
 0
 0
 0
 103

 8
 0
 0
 0
 0
 0
 0
 8

 1
 0
 0
 0
 0
 0
 0
 1

 7
 0
 0
 0
 0
 0
 0
 7

 88
 0
 0
 0
 0
 0
 0
 88

 88
 0
 0
 0
 0
 0
 0
 88

 5
 0
 0
 0
 0
 0
 0
 5

 3
 0
 0
 0
 0
 0
 0
 3

 2
 0
 0
 0
 0
 0
 0
 2

 2
 0
 0
 0
 0
 0
 0
 2

 2
 0
 0
 0
 0
 0
 0
 2

 6
 98
 2
 206
 0
 0
 0
 312

 6
 98
 2
 206
 0
 0
 0
 312

 6
 83
 2
 180
 0
 0
 0
 271

 0
 15
 0
 26
 0
 0
 0
 41

 2393
 53
 12
 11
 6
 1
 867
 3343

 366
 53
 12
 11
 6
 1
 829
 1278

 310
 0
 12
 11
 6
 1
 829
 1169

 138
 0
 0
 0
 0
 0
 0
 138

 70
 0
 0
 0
 0
 0
 0
 70

 1
 0
 0
 11
 0
 0
 0
 12

 2
 0
 0
 0
 0
 0
 42
 44

 4
 0
 0
 0
 0
 0
 46
 50

 1
 0
 0
 0
 4
 0
 161
 166

 1
 0
 0
 0
 0
 0
 0
 1

 75
 0
 0
 0
 0
 0
 0
 75

 2
 0
 0
 0
 0
 0
 0
 2

 1
 0
 0
 0
 0
 0
 0
 1

 12
 0
 0
 0
 0
 0
 3
 15

 1
 0
 0
 0
 1
 0
 219
 221

 2
 0
 10
 0
 0
 1
 204
 217

 0
 0
 2
 0
 0
 0
 133
 135

 0
 0
 0
 0
 1
 0
 10
 11

 0
 0
 0
 0
 0
 0
 2
 2

 0
 0
 0
 0
 0
 0
 1
 1

 0
 0
 0
 0
 0
 0
 4
 4

 0
 0
 0
 0
 0
 0
 4
 4

 14
 0
 0
 0
 0
 0
 0
 14

 1
 0
 0
 0
 0
 0
 0
 1

 11
 0
 0
 0
 0
 0
 0
 11

 2
 0
 0
 0
 0
 0
 0
 2

 40
 53
 0
 0
 0
 0
 0
 93

 4
 0
 0
 0
 0
 0
 0
 4

 12
 0
 0
 0
 0
 0
 0
 12

 13
 0
 0
 0
 0
 0
 0
 13

 2
 53
 0
 0
 0
 0
 0
 55

 6
 0
 0
 0
 0
 0
 0
 6

 3
 0
 0
 0
 0
 0
 0
 3

 1
 0
 0
 0
 0
 0
 0
 1

 1
 0
 0
 0
 0
 0
 0
 1

 1
 0
 0
 0
 0
 0
 0
 1

 1
 0
 0
 0
 0
 0
 0
 1

 2027
 0
 0
 0
 0
 0
 38
 2065

 2027
 0
 0
 0
 0
 0
 38
 2065

 226
 0
 0
 0
 0
 0
 38
 264

 128
 0
 0
 0
 0
 0
 0
 128

 1671
 0
 0
 0
 0
 0
 0
 1671

 2
 0
 0
 0
 0
 0
 0
 2

 35
 0
 0
 0
 0
 0
 0
 35

 35
 0
 0
 0
 0
 0
 0
 35

 34
 0
 0
 0
 0
 0
 0
 34

 13
 0
 0
 0
 0
 0
 0
 13

 1
 0
 0
 0
 0
 0
 0
 1

 5
 0
 0
 0
 0
 0
 0
 5

 15
 0
 0
 0
 0
 0
 0
 15

 1
 0
 0
 0
 0
 0
 0
 1

 1
 0
 0
 0
 0
 0
 0
 1

 96
 0
 0
 0
 0
 0
 0
 96

 96
 0
 0
 0
 0
 0
 0
 96

 96
 0
 0
 0
 0
 0
 0
 96

 96
 0
 0
 0
 0
 0
 0
 96

 423
 1
 0
 0
 4
 95
 2
 525

 10
 0
 0
 0
 0
 0
 0
 10

 10
 0
 0
 0
 0
 0
 0
 10

 10
 0
 0
 0
 0
 0
 0
 10

 10
 0
 0
 0
 0
 0
 0
 10

 413
 1
 0
 0
 4
 95
 2
 515

 2
 0
 0
 0
 0
 0
 2
 4

 2
 0
 0
 0
 0
 0
 0
 2

 2
 0
 0
 0
 0
 0
 0
 2

 0
 0
 0
 0
 0
 0
 2
 2

 0
 0
 0
 0
 0
 0
 2
 2

 411
 1
 0
 0
 4
 95
 0
 511

 378
 1
 0
 0
 0
 0
 0
 379

 378
 1
 0
 0
 0
 0
 0
 379

 17
 0
 0
 0
 0
 5
 0
 22

 17
 0
 0
 0
 0
 5
 0
 22

 16
 0
 0
 0
 4
 90
 0
 110

 16
 0
 0
 0
 0
 0
 0
 16

 0
 0
 0
 0
 4
 90
 0
 94

 1999
 0
 0
 53
 9
 2161
 26
 4248

 17
 0
 0
 0
 0
 0
 0
 17

 17
 0
 0
 0
 0
 0
 0
 17

 8
 0
 0
 0
 0
 0
 0
 8

 8
 0
 0
 0
 0
 0
 0
 8

 8
 0
 0
 0
 0
 0
 0
 8

 9
 0
 0
 0
 0
 0
 0
 9

 9
 0
 0
 0
 0
 0
 0
 9

 9
 0
 0
 0
 0
 0
 0
 9

 1982
 0
 0
 53
 9
 2161
 26
 4231

 1
 0
 0
 0
 0
 0
 0
 1

 1
 0
 0
 0
 0
 0
 0
 1

 1
 0
 0
 0
 0
 0
 0
 1

 1
 0
 0
 0
 0
 0
 0
 1

 1251
 0
 0
 0
 1
 76
 0
 1328

 557
 0
 0
 0
 0
 0
 0
 557

 554
 0
 0
 0
 0
 0
 0
 554

 554
 0
 0
 0
 0
 0
 0
 554

 3
 0
 0
 0
 0
 0
 0
 3

 3
 0
 0
 0
 0
 0
 0
 3

 681
 0
 0
 0
 0
 0
 0
 681

 681
 0
 0
 0
 0
 0
 0
 681

 681
 0
 0
 0
 0
 0
 0
 681

 10
 0
 0
 0
 1
 76
 0
 87

 10
 0
 0
 0
 0
 0
 0
 10

 10
 0
 0
 0
 0
 0
 0
 10

 0
 0
 0
 0
 1
 76
 0
 77

 0
 0
 0
 0
 1
 76
 0
 77

 3
 0
 0
 0
 0
 0
 0
 3

 3
 0
 0
 0
 0
 0
 0
 3

 1
 0
 0
 0
 0
 0
 0
 1

 2
 0
 0
 0
 0
 0
 0
 2

 702
 0
 0
 53
 0
 0
 17
 772

 665
 0
 0
 53
 0
 0
 16
 734

 424
 0
 0
 53
 0
 0
 16
 493

 393
 0
 0
 53
 0
 0
 2
 448

 31
 0
 0
 0
 0
 0
 14
 45

 51
 0
 0
 0
 0
 0
 0
 51

 1
 0
 0
 0
 0
 0
 0
 1

 50
 0
 0
 0
 0
 0
 0
 50

 190
 0
 0
 0
 0
 0
 0
 190

 190
 0
 0
 0
 0
 0
 0
 190

 35
 0
 0
 0
 0
 0
 1
 36

 35
 0
 0
 0
 0
 0
 0
 35

 35
 0
 0
 0
 0
 0
 0
 35

 0
 0
 0
 0
 0
 0
 1
 1

 0
 0
 0
 0
 0
 0
 1
 1

 2
 0
 0
 0
 0
 0
 0
 2

 2
 0
 0
 0
 0
 0
 0
 2

 2
 0
 0
 0
 0
 0
 0
 2

 3
 0
 0
 0
 0
 0
 0
 3

 3
 0
 0
 0
 0
 0
 0
 3

 3
 0
 0
 0
 0
 0
 0
 3

 3
 0
 0
 0
 0
 0
 0
 3

 17
 0
 0
 0
 2
 4
 3
 26

 17
 0
 0
 0
 2
 0
 3
 22

 17
 0
 0
 0
 2
 0
 3
 22

 17
 0
 0
 0
 2
 0
 3
 22

 0
 0
 0
 0
 0
 4
 0
 4

 0
 0
 0
 0
 0
 4
 0
 4

 0
 0
 0
 0
 0
 4
 0
 4

 3
 0
 0
 0
 0
 0
 0
 3

 3
 0
 0
 0
 0
 0
 0
 3

 3
 0
 0
 0
 0
 0
 0
 3

 3
 0
 0
 0
 0
 0
 0
 3

 5
 0
 0
 0
 0
 0
 0
 5

 5
 0
 0
 0
 0
 0
 0
 5

 4
 0
 0
 0
 0
 0
 0
 4

 4
 0
 0
 0
 0
 0
 0
 4

 1
 0
 0
 0
 0
 0
 0
 1

 1
 0
 0
 0
 0
 0
 0
 1

 0
 0
 0
 0
 6
 2081
 6
 2093

 0
 0
 0
 0
 6
 2081
 6
 2093

 0
 0
 0
 0
 6
 2081
 6
 2093

 0
 0
 0
 0
 6
 2081
 6
 2093

 167222
 9316
 94108
 40974
 138348
 1644
 31907
 483519

 157199
 9072
 94020
 40474
 128621
 1583
 31841
 462810

 139816
 8071
 93709
 36249
 102374
 1521
 30755
 412495

 139816
 8071
 93709
 36249
 102374
 1521
 30755
 412495

 105553
 5286
 26114
 8872
 6883
 1157
 27585
 181450

 2
 0
 0
 1
 0
 0
 0
 3

 4
 0
 0
 1
 0
 0
 0
 5

 216
 16
 5
 0
 0
 0
 6
 243

 1032
 4
 4
 4
 0
 0
 0
 1044

 1
 0
 0
 0
 0
 0
 0
 1

 291
 0
 2
 133
 1
 13
 23
 463

 1794
 1486
 19066
 696
 23
 5
 2868
 25938

 10025
 51
 333
 463
 337
 118
 23362
 34689

 41
 0
 1
 4
 0
 0
 2
 48

 2
 0
 1
 0
 0
 0
 0
 3

 10
 14
 13
 15
 0
 0
 2
 54

 54
 0
 18
 4
 0
 0
 0
 76

 62910
 1638
 4354
 4120
 107
 968
 648
 74745

 809
 29
 135
 76
 5
 0
 123
 1177

 2
 1
 0
 0
 0
 0
 0
 3

 7751
 798
 14
 206
 1
 0
 0
 8770

 5558
 72
 7
 0
 0
 0
 34
 5671

 5
 1
 1
 0
 1
 0
 0
 8

 1544
 0
 852
 393
 38
 0
 0
 2827

 13499
 1176
 1308
 2738
 6370
 53
 506
 25650

 1
 0
 0
 0
 0
 0
 0
 1

 2
 0
 0
 4
 0
 0
 0
 6

 0
 0
 0
 3
 0
 0
 0
 3

 0
 0
 0
 10
 0
 0
 0
 10

 0
 0
 0
 1
 0
 0
 0
 1

 0
 0
 0
 0
 0
 0
 11
 11

 666
 1
 147
 48
 53029
 88
 907
 54886

 7
 0
 0
 0
 3261
 5
 9
 3282

 161
 0
 0
 8
 32902
 54
 77
 33202

 4
 0
 0
 0
 0
 0
 0
 4

 453
 0
 0
 4
 16561
 29
 35
 17082

 12
 0
 0
 4
 0
 0
 1
 17

 29
 0
 101
 14
 0
 0
 0
 144

 0
 1
 45
 11
 60
 0
 785
 902

 0
 0
 1
 0
 0
 0
 0
 1

 0
 0
 0
 7
 245
 0
 0
 252

 2701
 1548
 1840
 18409
 2
 172
 64
 24736

 1815
 406
 53
 0
 0
 0
 0
 2274

 212
 0
 0
 8
 0
 0
 0
 220

 333
 2
 26
 0
 0
 144
 3
 508

 30
 6
 91
 5396
 0
 0
 0
 5523

 8
 61
 0
 0
 0
 0
 0
 69

 184
 0
 0
 0
 0
 0
 0
 184

 51
 2
 2
 0
 0
 28
 0
 83

 4
 0
 0
 0
 0
 0
 0
 4

 18
 0
 0
 10
 0
 0
 0
 28

 3
 4
 1481
 12686
 0
 0
 2
 14176

 38
 1067
 166
 123
 0
 0
 0
 1394

 4
 0
 0
 0
 0
 0
 0
 4

 1
 0
 0
 0
 0
 0
 0
 1

 0
 0
 17
 169
 1
 0
 0
 187

 0
 0
 4
 9
 0
 0
 0
 13

 0
 0
 0
 8
 0
 0
 0
 8

 0
 0
 0
 0
 1
 0
 52
 53

 0
 0
 0
 0
 0
 0
 7
 7

 30896
 1236
 65608
 8920
 42459
 104
 2199
 151422

 277
 5
 384
 442
 1039
 4
 67
 2218

 3875
 234
 23
 1
 0
 0
 0
 4133

 54
 0
 0
 0
 0
 0
 0
 54

 2
 0
 311
 34
 89
 0
 0
 436

 1345
 157
 136
 415
 25781
 82
 325
 28241

 861
 0
 23
 0
 0
 0
 0
 884

 138
 11
 60029
 792
 5196
 4
 20
 66190

 1052
 0
 1
 1
 10183
 14
 779
 12030

 6647
 349
 189
 17
 0
 0
 26
 7228

 52
 36
 3
 3559
 0
 0
 1
 3651

 78
 0
 0
 0
 0
 0
 0
 78

 1544
 1
 1403
 327
 0
 0
 1
 3276

 64
 3
 16
 65
 3
 0
 2
 153

 111
 0
 220
 451
 8
 0
 0
 790

 36
 0
 0
 0
 0
 0
 362
 398

 111
 1
 13
 3
 0
 0
 0
 128

 14649
 439
 2851
 2813
 160
 0
 616
 21528

 0
 0
 6
 0
 0
 0
 0
 6

 0
 0
 0
 0
 1
 0
 0
 1

 0
 0
 0
 0
 1
 0
 0
 1

 15786
 988
 145
 4200
 26234
 62
 880
 48295

 15786
 988
 145
 4200
 26234
 62
 880
 48295

 15786
 988
 145
 4200
 26234
 62
 880
 48295

 2
 0
 0
 0
 0
 0
 1
 3

 6857
 866
 43
 157
 26228
 60
 518
 34729

 384
 5
 20
 3914
 1
 0
 20
 4344

 206
 0
 0
 0
 0
 0
 0
 206

 1
 0
 3
 0
 0
 0
 0
 4

 90
 0
 5
 0
 0
 0
 0
 95

 8
 0
 2
 0
 0
 0
 0
 10

 594
 66
 53
 110
 3
 2
 324
 1152

 15
 0
 7
 0
 0
 0
 0
 22

 19
 2
 0
 2
 0
 0
 15
 38

 1
 0
 0
 0
 0
 0
 0
 1

 2
 0
 0
 1
 0
 0
 0
 3

 6
 49
 10
 5
 0
 0
 0
 70

 60
 0
 0
 0
 0
 0
 0
 60

 37
 0
 2
 2
 0
 0
 0
 41

 2
 0
 0
 4
 0
 0
 0
 6

 7502
 0
 0
 0
 2
 0
 0
 7504

 0
 0
 0
 5
 0
 0
 0
 5

 0
 0
 0
 0
 0
 0
 1
 1

 0
 0
 0
 0
 0
 0
 1
 1

 112
 0
 0
 0
 0
 0
 0
 112

 112
 0
 0
 0
 0
 0
 0
 112

 111
 0
 0
 0
 0
 0
 0
 111

 111
 0
 0
 0
 0
 0
 0
 111

 1
 0
 0
 0
 0
 0
 0
 1

 1
 0
 0
 0
 0
 0
 0
 1

 81
 1
 0
 8
 0
 0
 31
 121

 81
 0
 0
 0
 0
 0
 31
 112

 81
 0
 0
 0
 0
 0
 31
 112

 59
 0
 0
 0
 0
 0
 19
 78

 22
 0
 0
 0
 0
 0
 0
 22

 0
 0
 0
 0
 0
 0
 2
 2

 0
 0
 0
 0
 0
 0
 10
 10

 0
 1
 0
 8
 0
 0
 0
 9

 0
 1
 0
 0
 0
 0
 0
 1

 0
 1
 0
 0
 0
 0
 0
 1

 0
 0
 0
 8
 0
 0
 0
 8

 0
 0
 0
 8
 0
 0
 0
 8

 701
 12
 166
 11
 6
 0
 175
 1071

 701
 12
 166
 11
 6
 0
 175
 1071

 375
 0
 0
 0
 0
 0
 0
 375

 1
 0
 0
 0
 0
 0
 0
 1

 18
 0
 0
 0
 0
 0
 0
 18

 4
 0
 0
 0
 0
 0
 0
 4

 352
 0
 0
 0
 0
 0
 0
 352

 75
 12
 0
 10
 0
 0
 175
 272

 3
 0
 0
 0
 0
 0
 10
 13

 72
 12
 0
 10
 0
 0
 165
 259

 210
 0
 0
 0
 0
 0
 0
 210

 210
 0
 0
 0
 0
 0
 0
 210

 22
 0
 0
 0
 0
 0
 0
 22

 22
 0
 0
 0
 0
 0
 0
 22

 19
 0
 166
 1
 6
 0
 0
 192

 19
 0
 166
 1
 6
 0
 0
 192

 687
 0
 0
 6
 7
 0
 0
 700

 687
 0
 0
 6
 7
 0
 0
 700

 197
 0
 0
 0
 0
 0
 0
 197

 171
 0
 0
 0
 0
 0
 0
 171

 26
 0
 0
 0
 0
 0
 0
 26

 2
 0
 0
 0
 7
 0
 0
 9

 2
 0
 0
 0
 0
 0
 0
 2

 0
 0
 0
 0
 7
 0
 0
 7

 7
 0
 0
 0
 0
 0
 0
 7

 5
 0
 0
 0
 0
 0
 0
 5

 2
 0
 0
 0
 0
 0
 0
 2

 481
 0
 0
 6
 0
 0
 0
 487

 367
 0
 0
 0
 0
 0
 0
 367

 114
 0
 0
 0
 0
 0
 0
 114

 0
 0
 0
 6
 0
 0
 0
 6

 2
 0
 0
 0
 0
 0
 0
 2

 2
 0
 0
 0
 0
 0
 0
 2

 2
 0
 0
 0
 0
 0
 0
 2

 2
 0
 0
 0
 0
 0
 0
 2

 10
 0
 0
 0
 0
 0
 0
 10

 10
 0
 0
 0
 0
 0
 0
 10

 10
 0
 0
 0
 0
 0
 0
 10

 10
 0
 0
 0
 0
 0
 0
 10

 3
 0
 0
 0
 0
 0
 0
 3

 3
 0
 0
 0
 0
 0
 0
 3

 3
 0
 0
 0
 0
 0
 0
 3

 3
 0
 0
 0
 0
 0
 0
 3

 1
 0
 0
 0
 0
 0
 0
 1

 1
 0
 0
 0
 0
 0
 0
 1

 1
 0
 0
 0
 0
 0
 0
 1

 1
 0
 0
 0
 0
 0
 0
 1

 961
 0
 46
 405
 9485
 61
 23
 10981

 425
 0
 46
 405
 9485
 61
 23
 10445

 425
 0
 46
 405
 9485
 61
 23
 10445

 21
 0
 0
 0
 9484
 17
 23
 9545

 21
 0
 0
 0
 9484
 17
 23
 9545

 3
 0
 0
 0
 0
 0
 0
 3

 3
 0
 0
 0
 0
 0
 0
 3

 151
 0
 37
 0
 0
 0
 0
 188

 14
 0
 0
 0
 0
 0
 0
 14

 6
 0
 0
 0
 0
 0
 0
 6

 131
 0
 37
 0
 0
 0
 0
 168

 1
 0
 0
 0
 0
 0
 0
 1

 1
 0
 0
 0
 0
 0
 0
 1

 64
 0
 0
 0
 0
 0
 0
 64

 64
 0
 0
 0
 0
 0
 0
 64

 185
 0
 9
 405
 1
 44
 0
 644

 185
 0
 9
 405
 1
 44
 0
 644

 506
 0
 0
 0
 0
 0
 0
 506

 474
 0
 0
 0
 0
 0
 0
 474

 474
 0
 0
 0
 0
 0
 0
 474

 474
 0
 0
 0
 0
 0
 0
 474

 20
 0
 0
 0
 0
 0
 0
 20

 20
 0
 0
 0
 0
 0
 0
 20

 20
 0
 0
 0
 0
 0
 0
 20

 12
 0
 0
 0
 0
 0
 0
 12

 12
 0
 0
 0
 0
 0
 0
 12

 12
 0
 0
 0
 0
 0
 0
 12

 30
 0
 0
 0
 0
 0
 0
 30

 30
 0
 0
 0
 0
 0
 0
 30

 30
 0
 0
 0
 0
 0
 0
 30

 30
 0
 0
 0
 0
 0
 0
 30

 8750
 244
 42
 95
 14
 0
 18
 9163

 7833
 244
 42
 95
 14
 0
 18
 8246

 7362
 208
 42
 52
 9
 0
 12
 7685

 7362
 208
 42
 52
 9
 0
 12
 7685

 6632
 208
 9
 28
 9
 0
 1
 6887

 4
 0
 25
 0
 0
 0
 0
 29

 197
 0
 4
 3
 0
 0
 11
 215

 529
 0
 4
 21
 0
 0
 0
 554

 471
 36
 0
 43
 5
 0
 6
 561

 471
 36
 0
 43
 5
 0
 6
 561

 412
 0
 0
 9
 0
 0
 0
 421

 11
 0
 0
 0
 0
 0
 0
 11

 48
 36
 0
 33
 5
 0
 6
 128

 0
 0
 0
 1
 0
 0
 0
 1

 156
 0
 0
 0
 0
 0
 0
 156

 94
 0
 0
 0
 0
 0
 0
 94

 94
 0
 0
 0
 0
 0
 0
 94

 94
 0
 0
 0
 0
 0
 0
 94

 3
 0
 0
 0
 0
 0
 0
 3

 3
 0
 0
 0
 0
 0
 0
 3

 3
 0
 0
 0
 0
 0
 0
 3

 59
 0
 0
 0
 0
 0
 0
 59

 59
 0
 0
 0
 0
 0
 0
 59

 59
 0
 0
 0
 0
 0
 0
 59

 754
 0
 0
 0
 0
 0
 0
 754

 526
 0
 0
 0
 0
 0
 0
 526

 526
 0
 0
 0
 0
 0
 0
 526

 526
 0
 0
 0
 0
 0
 0
 526

 209
 0
 0
 0
 0
 0
 0
 209

 209
 0
 0
 0
 0
 0
 0
 209

 209
 0
 0
 0
 0
 0
 0
 209

 19
 0
 0
 0
 0
 0
 0
 19

 19
 0
 0
 0
 0
 0
 0
 19

 19
 0
 0
 0
 0
 0
 0
 19

 4
 0
 0
 0
 0
 0
 0
 4

 4
 0
 0
 0
 0
 0
 0
 4

 4
 0
 0
 0
 0
 0
 0
 4

 4
 0
 0
 0
 0
 0
 0
 4

 3
 0
 0
 0
 0
 0
 0
 3

 3
 0
 0
 0
 0
 0
 0
 3

 3
 0
 0
 0
 0
 0
 0
 3

 3
 0
 0
 0
 0
 0
 0
 3

 210
 0
 0
 0
 0
 0
 25
 235

 62
 0
 0
 0
 0
 0
 11
 73

 62
 0
 0
 0
 0
 0
 11
 73

 62
 0
 0
 0
 0
 0
 11
 73

 62
 0
 0
 0
 0
 0
 11
 73

 143
 0
 0
 0
 0
 0
 1
 144

 77
 0
 0
 0
 0
 0
 0
 77

 77
 0
 0
 0
 0
 0
 0
 77

 77
 0
 0
 0
 0
 0
 0
 77

 15
 0
 0
 0
 0
 0
 0
 15

 15
 0
 0
 0
 0
 0
 0
 15

 15
 0
 0
 0
 0
 0
 0
 15

 9
 0
 0
 0
 0
 0
 0
 9

 9
 0
 0
 0
 0
 0
 0
 9

 9
 0
 0
 0
 0
 0
 0
 9

 41
 0
 0
 0
 0
 0
 0
 41

 41
 0
 0
 0
 0
 0
 0
 41

 41
 0
 0
 0
 0
 0
 0
 41

 1
 0
 0
 0
 0
 0
 1
 2

 1
 0
 0
 0
 0
 0
 1
 2

 1
 0
 0
 0
 0
 0
 1
 2

 5
 0
 0
 0
 0
 0
 13
 18

 5
 0
 0
 0
 0
 0
 13
 18

 5
 0
 0
 0
 0
 0
 4
 9

 5
 0
 0
 0
 0
 0
 4
 9

 0
 0
 0
 0
 0
 0
 9
 9

 0
 0
 0
 0
 0
 0
 9
 9

 102
 0
 0
 0
 228
 0
 0
 330

 102
 0
 0
 0
 0
 0
 0
 102

 102
 0
 0
 0
 0
 0
 0
 102

 102
 0
 0
 0
 0
 0
 0
 102

 102
 0
 0
 0
 0
 0
 0
 102

 0
 0
 0
 0
 228
 0
 0
 228

 0
 0
 0
 0
 228
 0
 0
 228

 0
 0
 0
 0
 228
 0
 0
 228

 0
 0
 0
 0
 228
 0
 0
 228

 9160
 11924
 443
 2670
 2850
 155245
 115994
 298286

 8822
 11845
 397
 1112
 2850
 155245
 115992
 296263

 1989
 11107
 173
 839
 1221
 6
 74
 15409

 426
 2153
 61
 731
 1220
 6
 71
 4668

 426
 2153
 61
 731
 1220
 6
 71
 4668

 218
 1
 0
 11
 465
 3
 2
 700

 1
 0
 0
 0
 0
 0
 0
 1

 5
 107
 0
 3
 2
 0
 67
 184

 1
 0
 0
 0
 0
 0
 0
 1

 201
 2041
 61
 717
 753
 3
 2
 3778

 0
 3
 0
 0
 0
 0
 0
 3

 0
 1
 0
 0
 0
 0
 0
 1

 1437
 8954
 112
 105
 1
 0
 3
 10612

 1437
 8954
 112
 105
 1
 0
 3
 10612

 12
 0
 20
 0
 0
 0
 0
 32

 1
 8900
 13
 14
 1
 0
 3
 8932

 18
 0
 0
 0
 0
 0
 0
 18

 1311
 42
 54
 89
 0
 0
 0
 1496

 1
 0
 0
 0
 0
 0
 0
 1

 9
 1
 0
 1
 0
 0
 0
 11

 2
 0
 0
 0
 0
 0
 0
 2

 4
 0
 25
 1
 0
 0
 0
 30

 14
 0
 0
 0
 0
 0
 0
 14

 2
 0
 0
 0
 0
 0
 0
 2

 28
 0
 0
 0
 0
 0
 0
 28

 35
 11
 0
 0
 0
 0
 0
 46

 124
 0
 0
 3
 0
 0
 0
 127

 124
 0
 0
 3
 0
 0
 0
 127

 1
 0
 0
 0
 0
 0
 0
 1

 123
 0
 0
 3
 0
 0
 0
 126

 2
 0
 0
 0
 0
 0
 0
 2

 2
 0
 0
 0
 0
 0
 0
 2

 2
 0
 0
 0
 0
 0
 0
 2

 1190
 0
 0
 0
 0
 0
 0
 1190

 1190
 0
 0
 0
 0
 0
 0
 1190

 1190
 0
 0
 0
 0
 0
 0
 1190

 1128
 0
 0
 0
 0
 0
 0
 1128

 50
 0
 0
 0
 0
 0
 0
 50

 12
 0
 0
 0
 0
 0
 0
 12

 4809
 0
 0
 15
 0
 0
 41
 4865

 4807
 0
 0
 15
 0
 0
 41
 4863

 1769
 0
 0
 15
 0
 0
 3
 1787

 1769
 0
 0
 15
 0
 0
 3
 1787

 3038
 0
 0
 0
 0
 0
 38
 3076

 3031
 0
 0
 0
 0
 0
 38
 3069

 7
 0
 0
 0
 0
 0
 0
 7

 2
 0
 0
 0
 0
 0
 0
 2

 2
 0
 0
 0
 0
 0
 0
 2

 2
 0
 0
 0
 0
 0
 0
 2

 446
 0
 0
 66
 1
 0
 217
 730

 446
 0
 0
 66
 1
 0
 217
 730

 442
 0
 0
 3
 1
 0
 216
 662

 442
 0
 0
 3
 1
 0
 216
 662

 1
 0
 0
 26
 0
 0
 0
 27

 1
 0
 0
 26
 0
 0
 0
 27

 3
 0
 0
 37
 0
 0
 1
 41

 3
 0
 0
 0
 0
 0
 1
 4

 0
 0
 0
 37
 0
 0
 0
 37

 293
 0
 0
 0
 0
 0
 0
 293

 5
 0
 0
 0
 0
 0
 0
 5

 5
 0
 0
 0
 0
 0
 0
 5

 4
 0
 0
 0
 0
 0
 0
 4

 1
 0
 0
 0
 0
 0
 0
 1

 284
 0
 0
 0
 0
 0
 0
 284

 284
 0
 0
 0
 0
 0
 0
 284

 88
 0
 0
 0
 0
 0
 0
 88

 194
 0
 0
 0
 0
 0
 0
 194

 2
 0
 0
 0
 0
 0
 0
 2

 4
 0
 0
 0
 0
 0
 0
 4

 3
 0
 0
 0
 0
 0
 0
 3

 3
 0
 0
 0
 0
 0
 0
 3

 1
 0
 0
 0
 0
 0
 0
 1

 1
 0
 0
 0
 0
 0
 0
 1

 29
 0
 0
 0
 0
 0
 0
 29

 29
 0
 0
 0
 0
 0
 0
 29

 29
 0
 0
 0
 0
 0
 0
 29

 29
 0
 0
 0
 0
 0
 0
 29

 66
 0
 0
 0
 0
 0
 1
 67

 1
 0
 0
 0
 0
 0
 0
 1

 1
 0
 0
 0
 0
 0
 0
 1

 1
 0
 0
 0
 0
 0
 0
 1

 21
 0
 0
 0
 0
 0
 1
 22

 21
 0
 0
 0
 0
 0
 1
 22

 21
 0
 0
 0
 0
 0
 1
 22

 44
 0
 0
 0
 0
 0
 0
 44

 44
 0
 0
 0
 0
 0
 0
 44

 44
 0
 0
 0
 0
 0
 0
 44

 0
 738
 224
 192
 1628
 155239
 115659
 273680

 0
 738
 224
 192
 1628
 155239
 115659
 273680

 0
 738
 224
 192
 1628
 155239
 115659
 273680

 0
 592
 212
 88
 991
 155186
 115231
 272300

 0
 146
 12
 104
 637
 53
 428
 1380

 299
 13
 46
 1448
 0
 0
 0
 1806

 27
 0
 0
 0
 0
 0
 0
 27

 27
 0
 0
 0
 0
 0
 0
 27

 27
 0
 0
 0
 0
 0
 0
 27

 6
 0
 0
 0
 0
 0
 0
 6

 21
 0
 0
 0
 0
 0
 0
 21

 271
 13
 46
 1448
 0
 0
 0
 1778

 121
 9
 44
 15
 0
 0
 0
 189

 61
 6
 44
 13
 0
 0
 0
 124

 1
 0
 0
 0
 0
 0
 0
 1

 24
 0
 0
 0
 0
 0
 0
 24

 8
 6
 1
 12
 0
 0
 0
 27

 28
 0
 42
 0
 0
 0
 0
 70

 0
 0
 1
 1
 0
 0
 0
 2

 60
 3
 0
 0
 0
 0
 0
 63

 1
 0
 0
 0
 0
 0
 0
 1

 59
 3
 0
 0
 0
 0
 0
 62

 0
 0
 0
 2
 0
 0
 0
 2

 0
 0
 0
 2
 0
 0
 0
 2

 5
 0
 0
 0
 0
 0
 0
 5

 5
 0
 0
 0
 0
 0
 0
 5

 4
 0
 0
 0
 0
 0
 0
 4

 1
 0
 0
 0
 0
 0
 0
 1

 129
 0
 1
 1427
 0
 0
 0
 1557

 129
 0
 1
 1427
 0
 0
 0
 1557

 2
 0
 0
 0
 0
 0
 0
 2

 78
 0
 1
 1299
 0
 0
 0
 1378

 2
 0
 0
 0
 0
 0
 0
 2

 28
 0
 0
 2
 0
 0
 0
 30

 19
 0
 0
 126
 0
 0
 0
 145

 10
 4
 0
 6
 0
 0
 0
 20

 10
 4
 0
 6
 0
 0
 0
 20

 1
 4
 0
 6
 0
 0
 0
 11

 9
 0
 0
 0
 0
 0
 0
 9

 5
 0
 1
 0
 0
 0
 0
 6

 5
 0
 1
 0
 0
 0
 0
 6

 5
 0
 1
 0
 0
 0
 0
 6

 1
 0
 0
 0
 0
 0
 0
 1

 1
 0
 0
 0
 0
 0
 0
 1

 1
 0
 0
 0
 0
 0
 0
 1

 1
 0
 0
 0
 0
 0
 0
 1

 1
 0
 0
 0
 0
 0
 0
 1

 1
 0
 0
 0
 0
 0
 0
 1

 1
 0
 0
 0
 0
 0
 0
 1

 39
 66
 0
 110
 0
 0
 2
 217

 4
 0
 0
 0
 0
 0
 0
 4

 4
 0
 0
 0
 0
 0
 0
 4

 4
 0
 0
 0
 0
 0
 0
 4

 4
 0
 0
 0
 0
 0
 0
 4

 35
 66
 0
 110
 0
 0
 2
 213

 35
 66
 0
 110
 0
 0
 2
 213

 35
 66
 0
 110
 0
 0
 2
 213

 3
 0
 0
 18
 0
 0
 0
 21

 32
 0
 0
 92
 0
 0
 0
 124

 0
 4
 0
 0
 0
 0
 0
 4

 0
 5
 0
 0
 0
 0
 0
 5

 0
 2
 0
 0
 0
 0
 0
 2

 0
 1
 0
 0
 0
 0
 0
 1

 0
 54
 0
 0
 0
 0
 0
 54

 0
 0
 0
 0
 0
 0
 2
 2

 47135
 3115
 11945
 16581
 123726
 29075
 112406
 343983

 6134
 1060
 1156
 957
 38767
 15081
 13116
 76271

 5871
 706
 982
 358
 31149
 12890
 13080
 65036

 251
 14
 68
 31
 23371
 12859
 44
 36638

 95
 0
 0
 0
 0
 0
 0
 95

 95
 0
 0
 0
 0
 0
 0
 95

 124
 0
 0
 0
 66
 1
 0
 191

 124
 0
 0
 0
 66
 1
 0
 191

 31
 14
 68
 31
 23305
 12858
 44
 36351

 14
 14
 68
 31
 23305
 12858
 44
 36334

 17
 0
 0
 0
 0
 0
 0
 17

 1
 0
 0
 0
 0
 0
 0
 1

 1
 0
 0
 0
 0
 0
 0
 1

 120
 569
 902
 274
 3494
 27
 12959
 18345

 44
 9
 830
 14
 444
 0
 1
 1342

 29
 6
 2
 12
 335
 0
 1
 385

 15
 3
 828
 2
 109
 0
 0
 957

 36
 249
 59
 155
 1803
 22
 11315
 13639

 1
 0
 0
 0
 0
 0
 0
 1

 1
 0
 0
 0
 0
 0
 0
 1

 32
 249
 59
 154
 1803
 22
 11315
 13634

 1
 0
 0
 0
 0
 0
 0
 1

 1
 0
 0
 0
 0
 0
 0
 1

 0
 0
 0
 1
 0
 0
 0
 1

 23
 0
 0
 0
 0
 0
 55
 78

 23
 0
 0
 0
 0
 0
 55
 78

 1
 0
 0
 0
 0
 0
 0
 1

 1
 0
 0
 0
 0
 0
 0
 1

 15
 0
 0
 0
 0
 0
 91
 106

 15
 0
 0
 0
 0
 0
 91
 106

 1
 288
 6
 75
 1091
 2
 420
 1883

 1
 288
 6
 75
 1091
 2
 420
 1883

 0
 1
 0
 0
 11
 0
 0
 12

 0
 1
 0
 0
 11
 0
 0
 12

 0
 8
 0
 12
 144
 3
 887
 1054

 0
 8
 0
 12
 144
 3
 887
 1054

 0
 4
 0
 0
 0
 0
 0
 4

 0
 4
 0
 0
 0
 0
 0
 4

 0
 10
 7
 18
 1
 0
 190
 226

 0
 10
 7
 17
 1
 0
 190
 225

 0
 0
 0
 1
 0
 0
 0
 1

 5124
 0
 12
 28
 155
 0
 1
 5320

 4
 0
 0
 0
 0
 0
 0
 4

 4
 0
 0
 0
 0
 0
 0
 4

 9
 0
 0
 1
 148
 0
 1
 159

 9
 0
 0
 1
 148
 0
 1
 159

 107
 0
 0
 0
 7
 0
 0
 114

 104
 0
 0
 0
 7
 0
 0
 111

 3
 0
 0
 0
 0
 0
 0
 3

 5003
 0
 0
 0
 0
 0
 0
 5003

 66
 0
 0
 0
 0
 0
 0
 66

 4937
 0
 0
 0
 0
 0
 0
 4937

 1
 0
 0
 0
 0
 0
 0
 1

 1
 0
 0
 0
 0
 0
 0
 1

 0
 0
 12
 27
 0
 0
 0
 39

 0
 0
 11
 0
 0
 0
 0
 11

 0
 0
 1
 0
 0
 0
 0
 1

 0
 0
 0
 27
 0
 0
 0
 27

 240
 0
 0
 0
 0
 0
 0
 240

 130
 0
 0
 0
 0
 0
 0
 130

 20
 0
 0
 0
 0
 0
 0
 20

 110
 0
 0
 0
 0
 0
 0
 110

 110
 0
 0
 0
 0
 0
 0
 110

 110
 0
 0
 0
 0
 0
 0
 110

 14
 6
 0
 25
 0
 0
 5
 50

 4
 6
 0
 1
 0
 0
 0
 11

 4
 6
 0
 1
 0
 0
 0
 11

 10
 0
 0
 0
 0
 0
 0
 10

 10
 0
 0
 0
 0
 0
 0
 10

 0
 0
 0
 2
 0
 0
 0
 2

 0
 0
 0
 2
 0
 0
 0
 2

 0
 0
 0
 8
 0
 0
 0
 8

 0
 0
 0
 8
 0
 0
 0
 8

 0
 0
 0
 14
 0
 0
 0
 14

 0
 0
 0
 14
 0
 0
 0
 14

 0
 0
 0
 0
 0
 0
 5
 5

 0
 0
 0
 0
 0
 0
 5
 5

 120
 0
 0
 0
 4128
 4
 8
 4260

 120
 0
 0
 0
 4128
 4
 8
 4260

 120
 0
 0
 0
 4128
 4
 8
 4260

 1
 117
 0
 0
 0
 0
 0
 118

 1
 117
 0
 0
 0
 0
 0
 118

 1
 117
 0
 0
 0
 0
 0
 118

 1
 0
 0
 0
 1
 0
 63
 65

 1
 0
 0
 0
 1
 0
 63
 65

 1
 0
 0
 0
 1
 0
 63
 65

 203
 353
 167
 565
 5466
 2188
 30
 8972

 163
 210
 1
 190
 29
 0
 0
 593

 94
 210
 1
 174
 20
 0
 0
 499

 94
 210
 1
 174
 20
 0
 0
 499

 69
 0
 0
 16
 9
 0
 0
 94

 3
 0
 0
 0
 0
 0
 0
 3

 56
 0
 0
 0
 9
 0
 0
 65

 10
 0
 0
 0
 0
 0
 0
 10

 0
 0
 0
 16
 0
 0
 0
 16

 9
 0
 0
 0
 0
 0
 0
 9

 9
 0
 0
 0
 0
 0
 0
 9

 9
 0
 0
 0
 0
 0
 0
 9

 22
 1
 0
 375
 17
 0
 11
 426

 22
 1
 0
 375
 17
 0
 11
 426

 19
 0
 0
 0
 0
 0
 10
 29

 3
 0
 0
 0
 0
 0
 1
 4

 0
 1
 0
 375
 17
 0
 0
 393

 7
 0
 0
 0
 0
 0
 0
 7

 7
 0
 0
 0
 0
 0
 0
 7

 7
 0
 0
 0
 0
 0
 0
 7

 2
 142
 166
 0
 5414
 4
 13
 5741

 2
 142
 166
 0
 5414
 4
 13
 5741

 1
 142
 166
 0
 5414
 4
 13
 5740

 1
 0
 0
 0
 0
 0
 0
 1

 0
 0
 0
 0
 6
 2184
 6
 2196

 0
 0
 0
 0
 6
 2184
 6
 2196

 0
 0
 0
 0
 6
 2184
 6
 2196

 29
 0
 0
 0
 2147
 3
 2
 2181

 29
 0
 0
 0
 2147
 3
 2
 2181

 29
 0
 0
 0
 2147
 3
 2
 2181

 11
 0
 0
 0
 3
 0
 0
 14

 13
 0
 0
 0
 0
 0
 0
 13

 5
 0
 0
 0
 0
 0
 0
 5

 0
 0
 0
 0
 121
 0
 0
 121

 0
 0
 0
 0
 2023
 3
 2
 2028

 2
 0
 0
 0
 0
 0
 0
 2

 2
 0
 0
 0
 0
 0
 0
 2

 2
 0
 0
 0
 0
 0
 0
 2

 2
 0
 0
 0
 0
 0
 0
 2

 6
 0
 0
 0
 0
 0
 0
 6

 1
 0
 0
 0
 0
 0
 0
 1

 1
 0
 0
 0
 0
 0
 0
 1

 1
 0
 0
 0
 0
 0
 0
 1

 5
 0
 0
 0
 0
 0
 0
 5

 5
 0
 0
 0
 0
 0
 0
 5

 5
 0
 0
 0
 0
 0
 0
 5

 12
 0
 0
 0
 0
 0
 0
 12

 12
 0
 0
 0
 0
 0
 0
 12

 12
 0
 0
 0
 0
 0
 0
 12

 12
 0
 0
 0
 0
 0
 0
 12

 11
 1
 7
 34
 5
 0
 4
 62

 11
 1
 7
 34
 5
 0
 4
 62

 11
 1
 7
 34
 2
 0
 4
 59

 11
 1
 7
 34
 2
 0
 4
 59

 0
 0
 0
 0
 3
 0
 0
 3

 0
 0
 0
 0
 3
 0
 0
 3

 39420
 2037
 10789
 15624
 84959
 13994
 99279
 266102

 33948
 309
 133
 121
 14
 0
 325
 34850

 22877
 309
 114
 85
 2
 0
 61
 23448

 1314
 0
 0
 0
 0
 0
 0
 1314

 1314
 0
 0
 0
 0
 0
 0
 1314

 176
 0
 0
 0
 0
 0
 0
 176

 176
 0
 0
 0
 0
 0
 0
 176

 15485
 278
 114
 78
 2
 0
 61
 16018

 15485
 278
 114
 78
 2
 0
 61
 16018

 138
 0
 0
 0
 0
 0
 0
 138

 138
 0
 0
 0
 0
 0
 0
 138

 5764
 31
 0
 7
 0
 0
 0
 5802

 5737
 31
 0
 7
 0
 0
 0
 5775

 27
 0
 0
 0
 0
 0
 0
 27

 10835
 0
 19
 2
 12
 0
 264
 11132

 1493
 0
 0
 0
 0
 0
 0
 1493

 1493
 0
 0
 0
 0
 0
 0
 1493

 758
 0
 0
 0
 0
 0
 12
 770

 758
 0
 0
 0
 0
 0
 12
 770

 243
 0
 0
 0
 0
 0
 0
 243

 243
 0
 0
 0
 0
 0
 0
 243

 668
 0
 19
 0
 0
 0
 0
 687

 668
 0
 0
 0
 0
 0
 0
 668

 0
 0
 19
 0
 0
 0
 0
 19

 501
 0
 0
 0
 0
 0
 0
 501

 501
 0
 0
 0
 0
 0
 0
 501

 3085
 0
 0
 0
 12
 0
 252
 3349

 3085
 0
 0
 0
 12
 0
 252
 3349

 207
 0
 0
 2
 0
 0
 0
 209

 207
 0
 0
 2
 0
 0
 0
 209

 356
 0
 0
 0
 0
 0
 0
 356

 356
 0
 0
 0
 0
 0
 0
 356

 77
 0
 0
 0
 0
 0
 0
 77

 77
 0
 0
 0
 0
 0
 0
 77

 191
 0
 0
 0
 0
 0
 0
 191

 191
 0
 0
 0
 0
 0
 0
 191

 22
 0
 0
 0
 0
 0
 0
 22

 22
 0
 0
 0
 0
 0
 0
 22

 13
 0
 0
 0
 0
 0
 0
 13

 13
 0
 0
 0
 0
 0
 0
 13

 26
 0
 0
 0
 0
 0
 0
 26

 26
 0
 0
 0
 0
 0
 0
 26

 6
 0
 0
 0
 0
 0
 0
 6

 6
 0
 0
 0
 0
 0
 0
 6

 16
 0
 0
 0
 0
 0
 0
 16

 16
 0
 0
 0
 0
 0
 0
 16

 52
 0
 0
 0
 0
 0
 0
 52

 52
 0
 0
 0
 0
 0
 0
 52

 3121
 0
 0
 0
 0
 0
 0
 3121

 3121
 0
 0
 0
 0
 0
 0
 3121

 121
 0
 0
 34
 0
 0
 0
 155

 121
 0
 0
 34
 0
 0
 0
 155

 121
 0
 0
 34
 0
 0
 0
 155

 87
 0
 0
 0
 0
 0
 0
 87

 87
 0
 0
 0
 0
 0
 0
 87

 87
 0
 0
 0
 0
 0
 0
 87

 28
 0
 0
 0
 0
 0
 0
 28

 28
 0
 0
 0
 0
 0
 0
 28

 28
 0
 0
 0
 0
 0
 0
 28

 5472
 1727
 10646
 9795
 84770
 13524
 98938
 224872

 1423
 3
 8590
 103
 2721
 2
 411
 13253

 1333
 3
 8590
 38
 1475
 2
 198
 11639

 4
 0
 0
 0
 0
 0
 0
 4

 1041
 0
 0
 0
 57
 0
 3
 1101

 256
 0
 0
 30
 1411
 2
 192
 1891

 4
 0
 0
 0
 1
 0
 0
 5

 28
 0
 0
 0
 0
 0
 0
 28

 0
 3
 8588
 5
 0
 0
 1
 8597

 0
 0
 2
 3
 0
 0
 0
 5

 0
 0
 0
 0
 6
 0
 0
 6

 0
 0
 0
 0
 0
 0
 2
 2

 90
 0
 0
 30
 1246
 0
 213
 1579

 17
 0
 0
 27
 1
 0
 0
 45

 3
 0
 0
 3
 1245
 0
 213
 1464

 65
 0
 0
 0
 0
 0
 0
 65

 5
 0
 0
 0
 0
 0
 0
 5

 0
 0
 0
 35
 0
 0
 0
 35

 0
 0
 0
 35
 0
 0
 0
 35

 247
 10
 56
 5736
 10324
 13400
 93413
 123186

 238
 8
 51
 28
 256
 187
 1249
 2017

 4
 1
 0
 1
 47
 13
 1166
 1232

 8
 0
 6
 0
 13
 8
 2
 37

 6
 0
 0
 0
 7
 34
 0
 47

 220
 7
 4
 27
 184
 117
 81
 640

 0
 0
 41
 0
 0
 0
 0
 41

 0
 0
 0
 0
 4
 8
 0
 12

 0
 0
 0
 0
 1
 5
 0
 6

 0
 0
 0
 0
 0
 2
 0
 2

 9
 2
 5
 5708
 10068
 13213
 92164
 121169

 9
 2
 5
 5708
 10068
 13213
 92164
 121169

 1562
 1501
 176
 139
 266
 4
 4559
 8207

 39
 0
 2
 1
 13
 0
 9
 64

 39
 0
 2
 1
 13
 0
 7
 62

 0
 0
 0
 0
 0
 0
 2
 2

 6
 0
 0
 0
 0
 0
 0
 6

 6
 0
 0
 0
 0
 0
 0
 6

 5
 0
 0
 0
 0
 0
 0
 5

 5
 0
 0
 0
 0
 0
 0
 5

 287
 2
 6
 136
 176
 0
 3992
 4599

 38
 0
 0
 19
 0
 0
 54
 111

 1
 0
 0
 52
 175
 0
 25
 253

 248
 2
 5
 65
 1
 0
 3913
 4234

 0
 0
 1
 0
 0
 0
 0
 1

 1220
 1499
 29
 0
 77
 4
 556
 3385

 1220
 1499
 29
 0
 77
 4
 556
 3385

 3
 0
 0
 2
 0
 0
 0
 5

 3
 0
 0
 2
 0
 0
 0
 5

 2
 0
 133
 0
 0
 0
 0
 135

 2
 0
 133
 0
 0
 0
 0
 135

 0
 0
 6
 0
 0
 0
 0
 6

 0
 0
 6
 0
 0
 0
 0
 6

 0
 0
 0
 0
 0
 0
 2
 2

 0
 0
 0
 0
 0
 0
 2
 2

 2090
 197
 867
 57
 71444
 118
 325
 75098

 2090
 197
 867
 57
 71444
 118
 325
 75098

 2090
 197
 867
 57
 71444
 118
 325
 75098

 128
 0
 0
 39
 10
 0
 195
 372

 128
 0
 0
 39
 10
 0
 195
 372

 128
 0
 0
 39
 10
 0
 195
 372

 22
 14
 611
 2021
 5
 0
 34
 2707

 22
 1
 561
 1978
 0
 0
 0
 2562

 22
 0
 561
 1960
 0
 0
 0
 2543

 0
 1
 0
 18
 0
 0
 0
 19

 0
 13
 50
 43
 5
 0
 34
 145

 0
 13
 50
 43
 5
 0
 5
 116

 0
 0
 0
 0
 0
 0
 29
 29

 0
 2
 346
 1700
 0
 0
 1
 2049

 0
 2
 346
 1700
 0
 0
 1
 2049

 0
 2
 346
 1700
 0
 0
 1
 2049

 0
 1
 10
 5179
 175
 470
 15
 5850

 0
 1
 10
 5179
 175
 470
 15
 5850

 0
 1
 10
 5179
 175
 470
 15
 5850

 0
 1
 10
 5179
 175
 470
 15
 5850

 0
 0
 0
 529
 0
 0
 1
 530

 0
 0
 0
 529
 0
 0
 1
 530

 0
 0
 0
 529
 0
 0
 1
 530

 0
 0
 0
 529
 0
 0
 1
 530

 1577
 18
 0
 0
 0
 0
 11
 1606

 228
 0
 0
 0
 0
 0
 0
 228

 228
 0
 0
 0
 0
 0
 0
 228

 228
 0
 0
 0
 0
 0
 0
 228

 43
 0
 0
 0
 0
 0
 0
 43

 150
 0
 0
 0
 0
 0
 0
 150

 35
 0
 0
 0
 0
 0
 0
 35

 1349
 18
 0
 0
 0
 0
 11
 1378

 1083
 0
 0
 0
 0
 0
 11
 1094

 1083
 0
 0
 0
 0
 0
 11
 1094

 1083
 0
 0
 0
 0
 0
 11
 1094

 266
 18
 0
 0
 0
 0
 0
 284

 266
 18
 0
 0
 0
 0
 0
 284

 266
 18
 0
 0
 0
 0
 0
 284

 4
 0
 0
 0
 0
 0
 0
 4

 4
 0
 0
 0
 0
 0
 0
 4

 4
 0
 0
 0
 0
 0
 0
 4

 4
 0
 0
 0
 0
 0
 0
 4

 4
 0
 0
 0
 0
 0
 0
 4

 1164
 243
 54
 74
 3
 0
 35
 1573

 4
 0
 0
 0
 0
 0
 0
 4

 4
 0
 0
 0
 0
 0
 0
 4

 4
 0
 0
 0
 0
 0
 0
 4

 4
 0
 0
 0
 0
 0
 0
 4

 4
 0
 0
 0
 0
 0
 0
 4

 887
 243
 53
 69
 0
 0
 35
 1287

 887
 243
 53
 69
 0
 0
 35
 1287

 866
 0
 0
 69
 0
 0
 22
 957

 864
 0
 0
 69
 0
 0
 19
 952

 473
 0
 0
 27
 0
 0
 0
 500

 12
 0
 0
 0
 0
 0
 0
 12

 288
 0
 0
 37
 0
 0
 1
 326

 2
 0
 0
 0
 0
 0
 17
 19

 89
 0
 0
 5
 0
 0
 1
 95

 2
 0
 0
 0
 0
 0
 3
 5

 2
 0
 0
 0
 0
 0
 3
 5

 2
 0
 0
 0
 0
 0
 0
 2

 2
 0
 0
 0
 0
 0
 0
 2

 2
 0
 0
 0
 0
 0
 0
 2

 19
 243
 53
 0
 0
 0
 13
 328

 19
 243
 53
 0
 0
 0
 13
 328

 19
 243
 53
 0
 0
 0
 13
 328

 106
 0
 0
 0
 0
 0
 0
 106

 106
 0
 0
 0
 0
 0
 0
 106

 106
 0
 0
 0
 0
 0
 0
 106

 106
 0
 0
 0
 0
 0
 0
 106

 1
 0
 0
 0
 0
 0
 0
 1

 104
 0
 0
 0
 0
 0
 0
 104

 1
 0
 0
 0
 0
 0
 0
 1

 116
 0
 0
 0
 0
 0
 0
 116

 116
 0
 0
 0
 0
 0
 0
 116

 116
 0
 0
 0
 0
 0
 0
 116

 116
 0
 0
 0
 0
 0
 0
 116

 4
 0
 0
 0
 0
 0
 0
 4

 112
 0
 0
 0
 0
 0
 0
 112

 5
 0
 1
 5
 3
 0
 0
 14

 5
 0
 1
 5
 3
 0
 0
 14

 5
 0
 0
 0
 3
 0
 0
 8

 5
 0
 0
 0
 3
 0
 0
 8

 5
 0
 0
 0
 3
 0
 0
 8

 0
 0
 1
 5
 0
 0
 0
 6

 0
 0
 1
 5
 0
 0
 0
 6

 0
 0
 1
 5
 0
 0
 0
 6

 46
 0
 0
 0
 0
 0
 0
 46

 46
 0
 0
 0
 0
 0
 0
 46

 46
 0
 0
 0
 0
 0
 0
 46

 19
 0
 0
 0
 0
 0
 0
 19

 19
 0
 0
 0
 0
 0
 0
 19

 27
 0
 0
 0
 0
 0
 0
 27

 27
 0
 0
 0
 0
 0
 0
 27

 13
 0
 0
 0
 0
 0
 0
 13

 13
 0
 0
 0
 0
 0
 0
 13

 1
 0
 0
 0
 0
 0
 0
 1

 1
 0
 0
 0
 0
 0
 0
 1

 1
 0
 0
 0
 0
 0
 0
 1

 1
 0
 0
 0
 0
 0
 0
 1

 12
 0
 0
 0
 0
 0
 0
 12

 12
 0
 0
 0
 0
 0
 0
 12

 12
 0
 0
 0
 0
 0
 0
 12

 12
 0
 0
 0
 0
 0
 0
 12
