# Supplementary material for: ScillyHAB: A Multi-Disciplinary Survey of Harmful Marine Phytoplankton and Shellfish Toxins in the Isles of Scilly: Combining Citizen Science with State-of-the-Art Monitoring in an Isolated UK Island Territory
Source: Mar Drugs. 2025 Dec 15;23(12):478. doi: 10.3390/md23120478 (PMC12734806; doi:10.3390/md23120478)
Supplement: Supplementary file 1 [file marinedrugs-23-00478-s001.zip › marinedrugs-4017030-supplementary/ScillyHAB supplementary figures v1.pdf]

Figure S1. Interactive metagenomic viewer showing the proportion of reads that aligned to specific genera for each month of the study and for the entire study (see separate .html file).

Figure S2. Maximum likelihood (ML) inferred tree of *Alexandrium* based on sequences of LSU using 10 000 Bootstraps and BS scores of <70 not shown, the branch lengths are shown in scale.

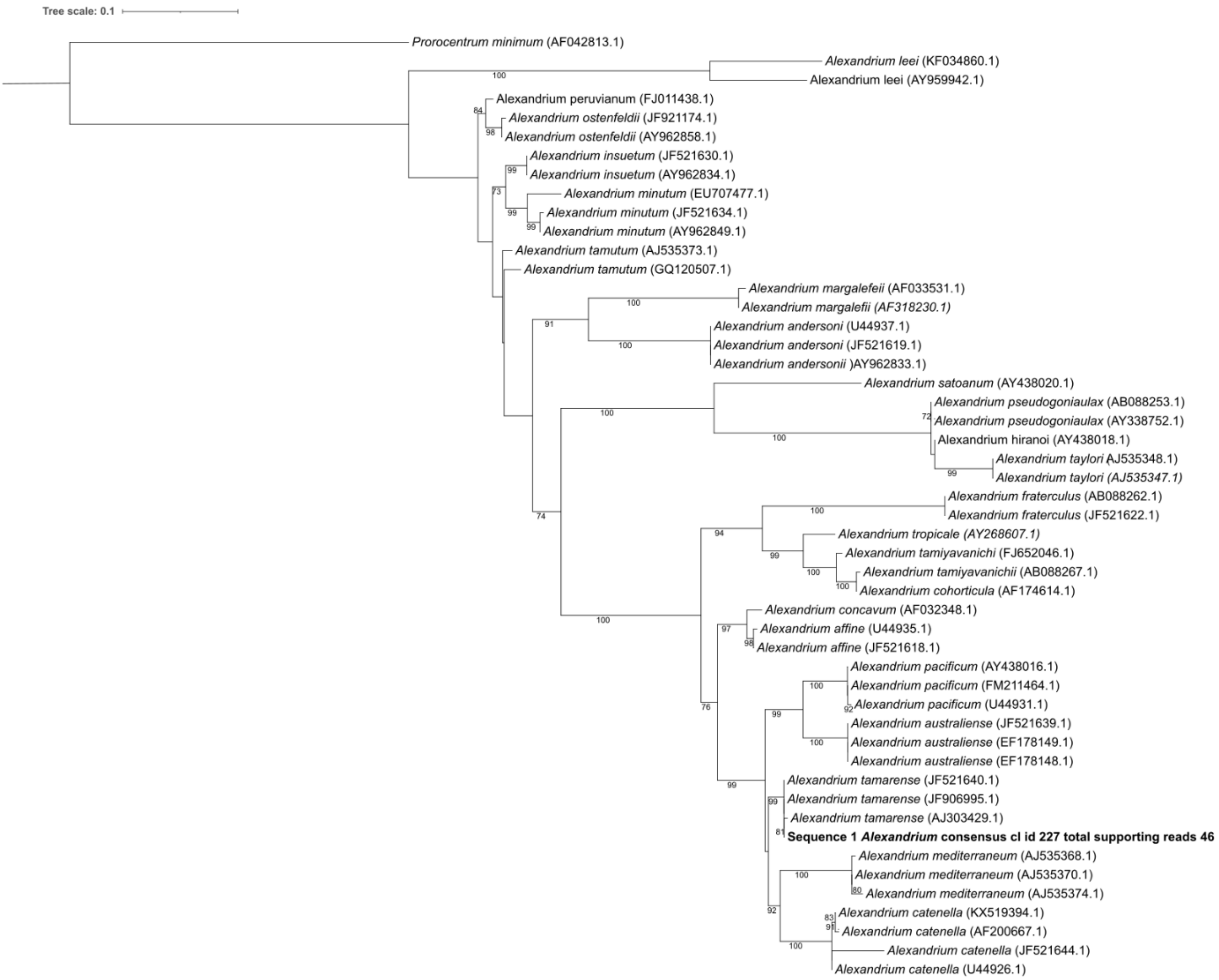

Figure: S3. Maximum likelihood (ML) inferred tree of *Azadinium* and *Amphidoma* based on sequences of internal transcribe spacer (ITS1-ITS2) using 10,000 Bootstraps and BS scores of <70 not shown. Branch lengths are shown in scale.

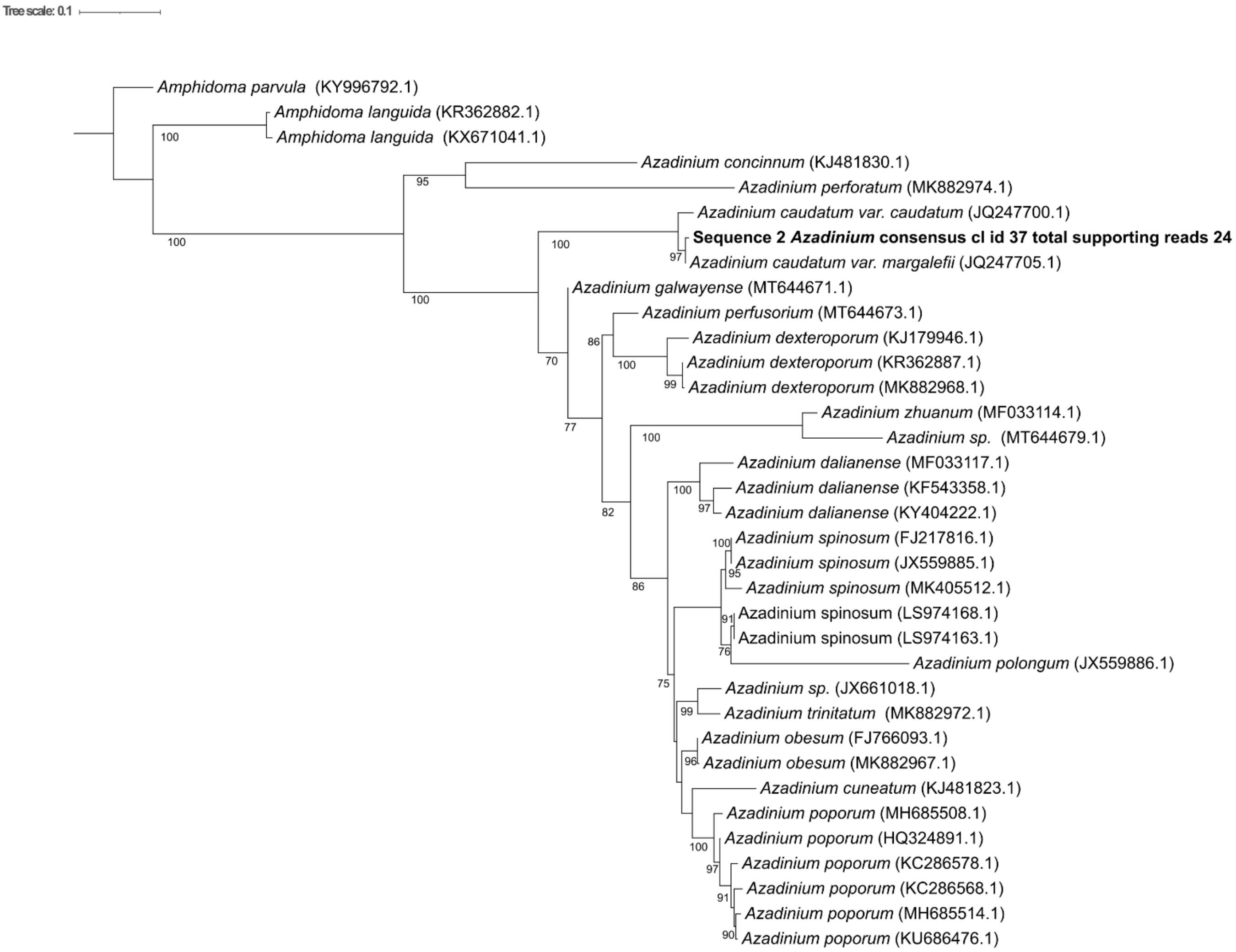

Figure S4. Maximum likelihood (ML) inferred tree of *Prorocentrum* based on sequences of internal transcribe spacer using 10 000 Bootstraps and BS scores of <70 not shown, the branch lengths are shown in scale.

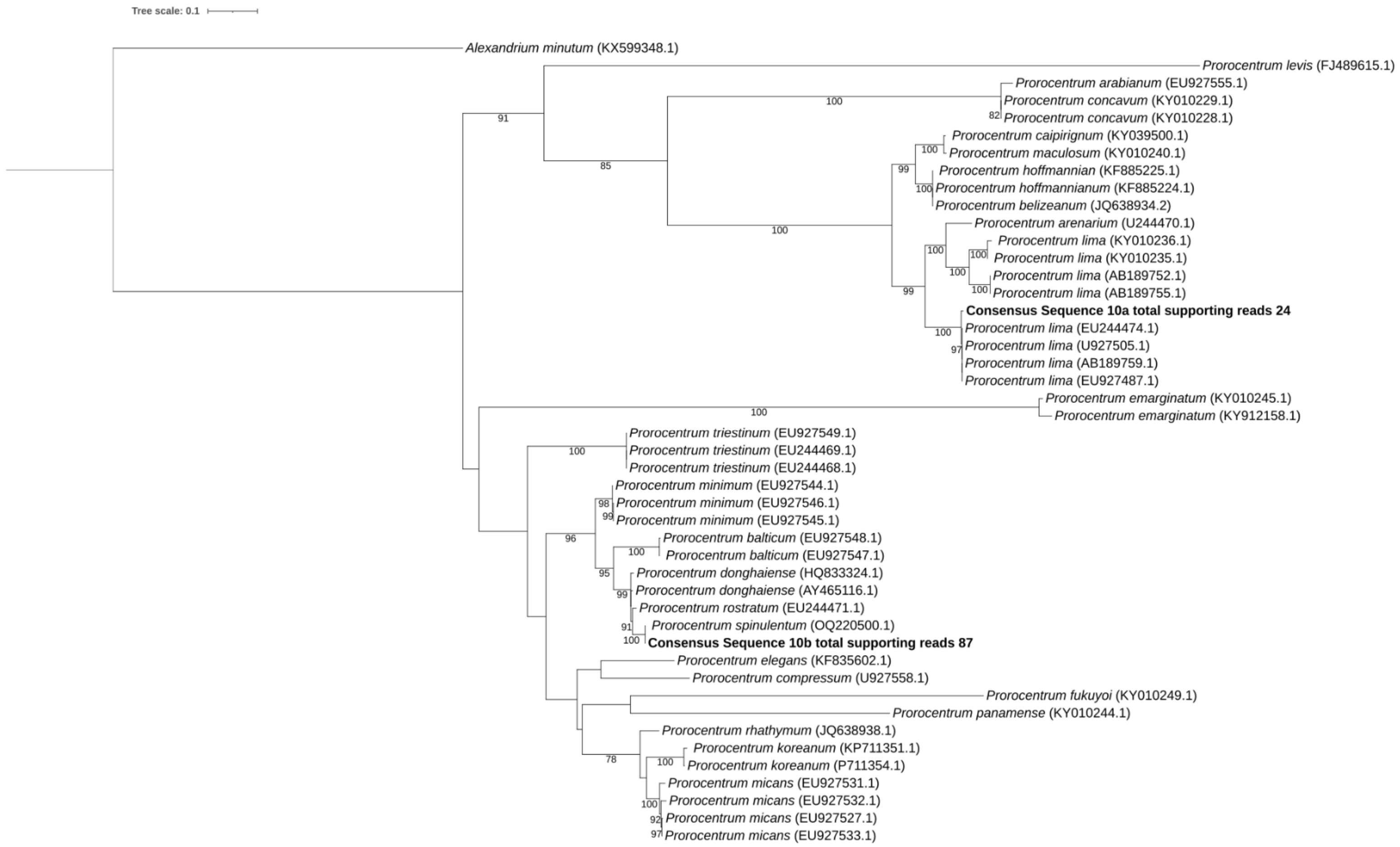

Figure S5. Maximum likelihood (ML) inferred tree of *Coolia*, including sequences aligning with *Karenia* and *Karlodinium* based on sequences of ITS using 10,000 Bootstraps, with scores of <70 not shown and branch lengths in scale.

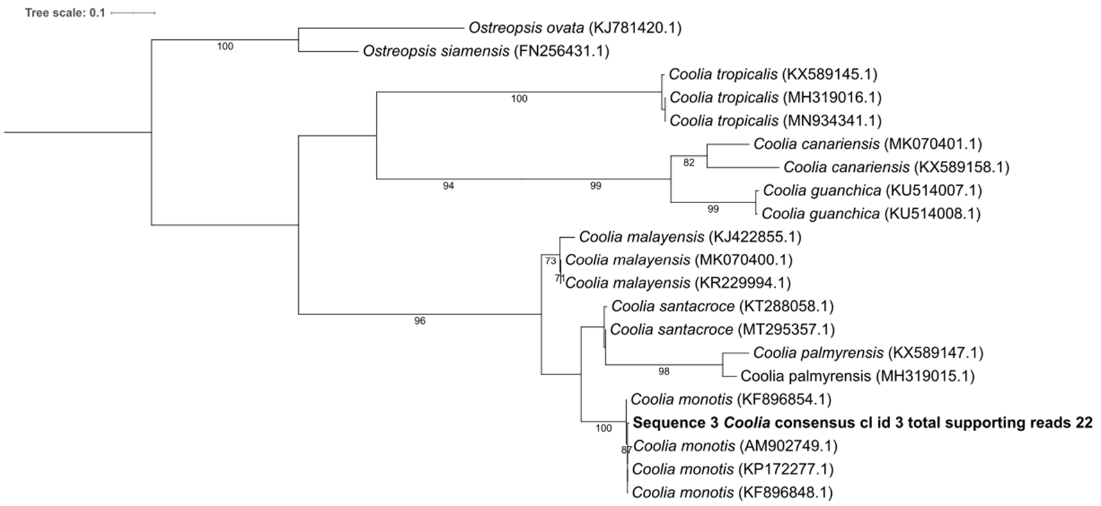

Figure S6. Maximum likelihood (ML) inferred tree of *Kareniacea*, including sequences aligning with *Karenia* and *Karlodinium* based on sequences of LSU using 10,000 Bootstraps and BS scores of <70 not shown, the branch lengths are shown in scale.

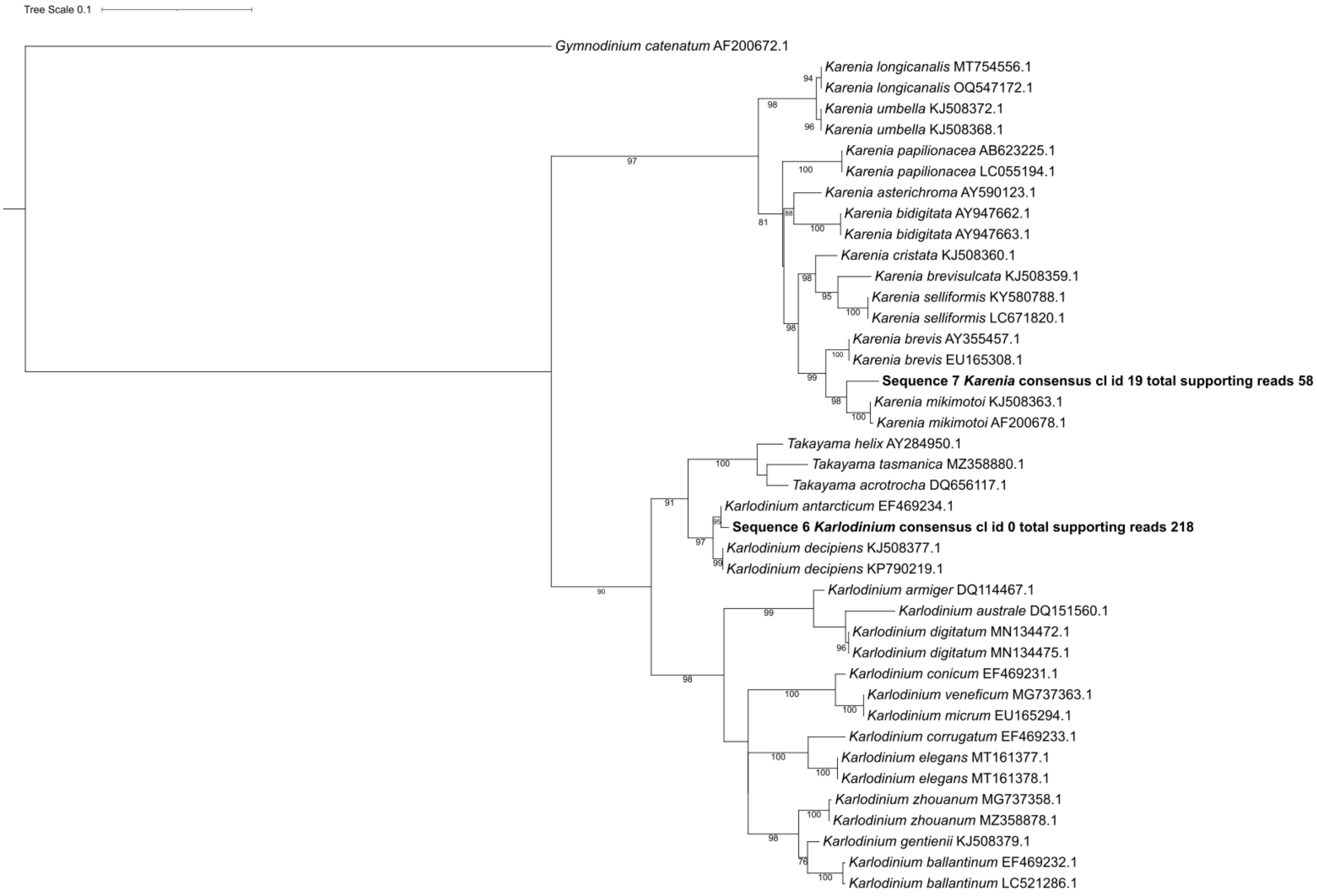

Figure S7. Maximum likelihood (ML) inferred tree of , including sequences aligning with *Kareniacea*, based on sequences of ITS using 10,000 Bootstraps and BS scores of <70 not shown, the branch lengths are shown in scale. Note: no reference sequence is available for ITS region of *K. antarcticum*.

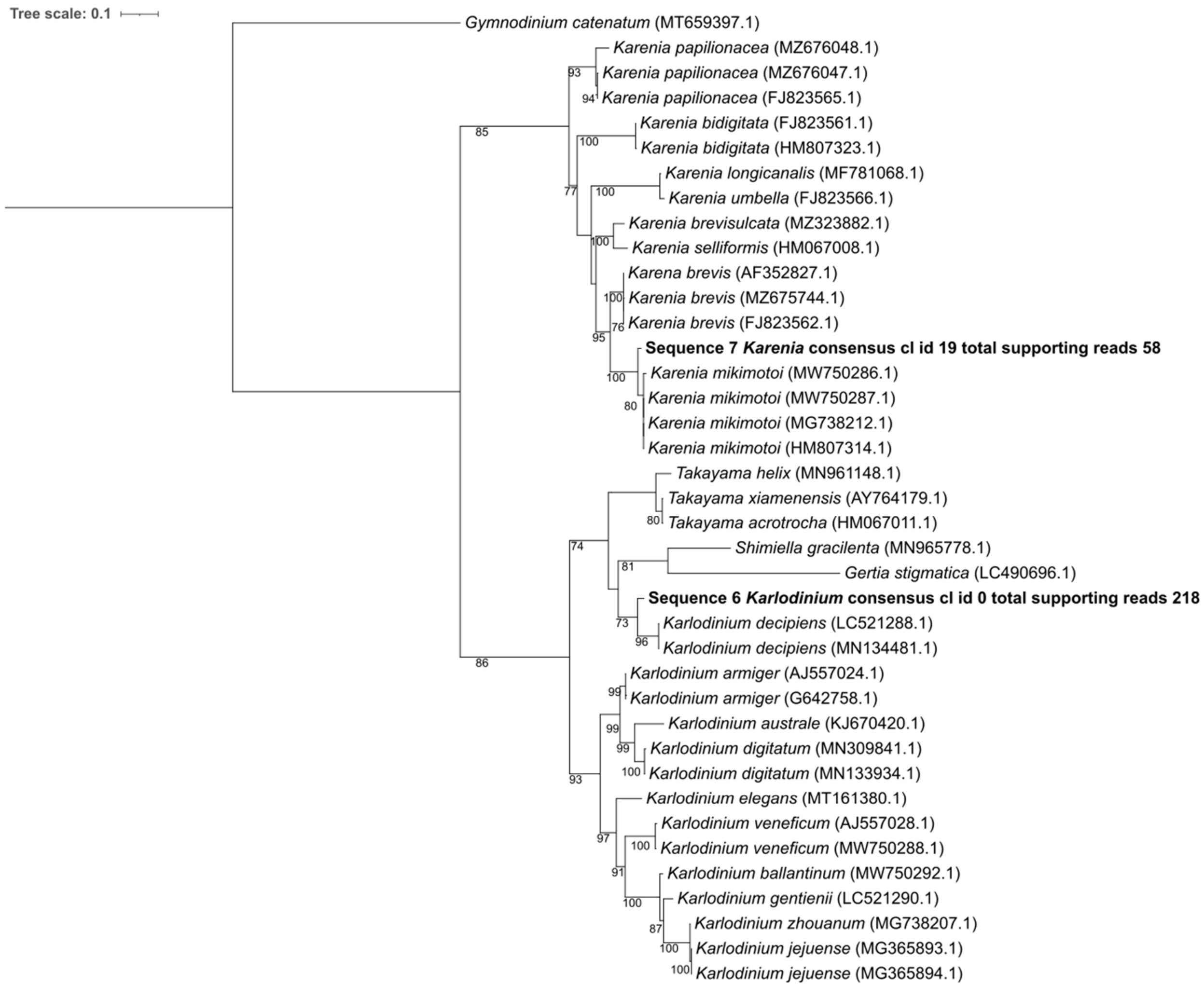

Figure S8. Maximum likelihood (ML) inferred tree of Dictyochyceans, including sequences aligning with Dictyocha and Aureococcus based on sequences of small sub unit (SSU/18S) using 10,000 Bootstraps with scores of <70 not shown and the branch lengths are shown in scale.

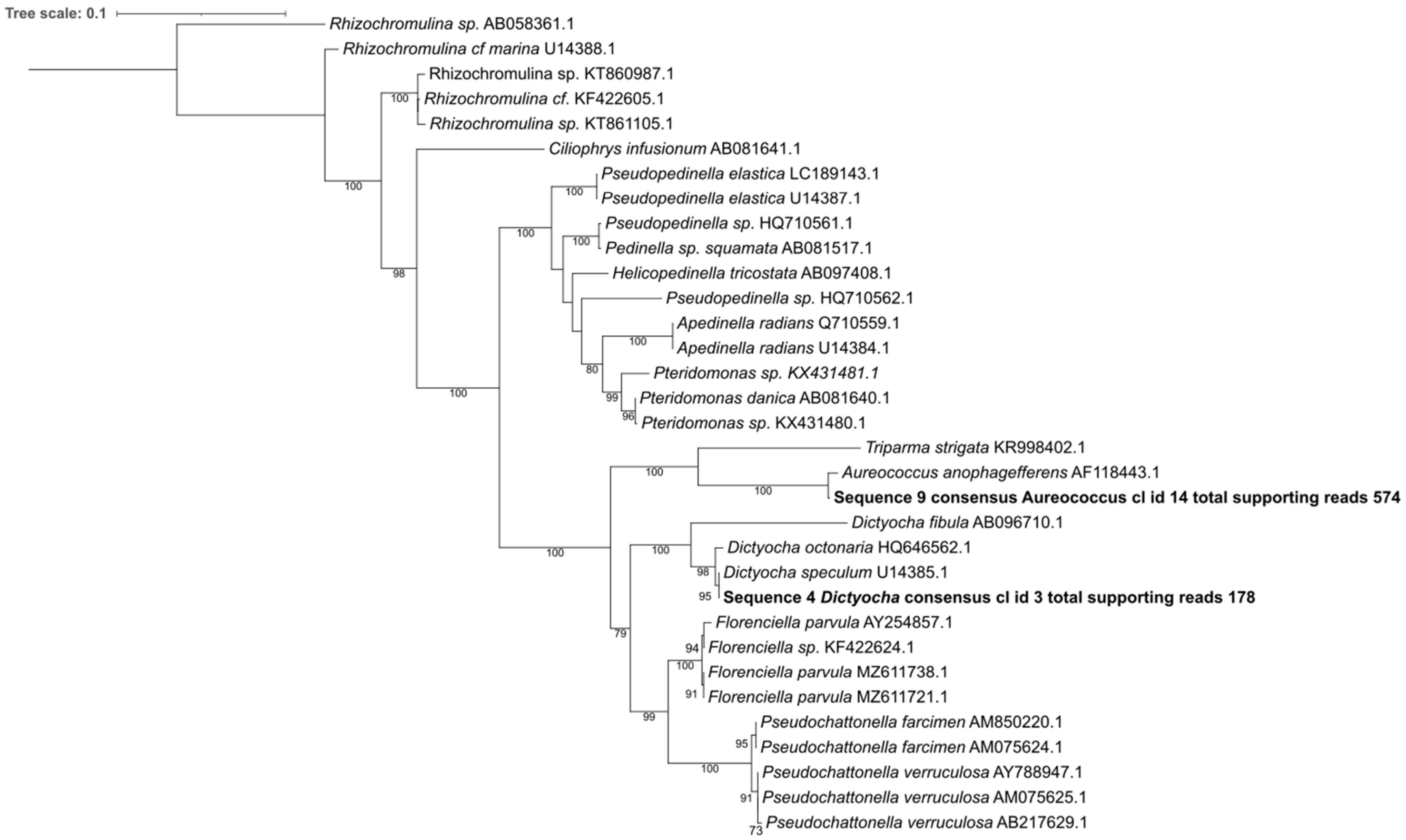

Figure S9. Negative Binomial Generalised Linear model output for the effect of Region, Time and Species on cell counts. *Pseudo nitzschia* and Mainland are set to the references.

Generalized Linear Model Regression Results

Dep. Variable:	Count	No. Observations:	299
Model:	GLM	Df Residuals:	289
Model Family:	NegativeBinomial	Df Model:	9
Link Function:	Log	Scale:	1.0000
Method:	IRLS	Log-Likelihood:	-2882.6
Date:	Mon, 22 Sep 2025	Deviance:	774.97
Time:	14:58:42	Pearson chi2:	894.
No. Iterations:	33	Pseudo R-squ. (CS):	0.9878
Covariance Type:	nonrobust		

	coef	std err	z	P>|z|	[0.025	0.975]
Intercept	5.4660	0.291	18.769	0.000	4.895	6.037
C(Species, Treatment(reference='Pseudo-nitzschia'))[T.Alexandrium]	-2.5031	0.487	-5.138	0.000	-3.458	-1.548
C(Species, Treatment(reference='Pseudo-nitzschia'))[T.Dinophysieae]	-1.5526	0.422	-3.681	0.000	-2.379	-0.726
C(Species, Treatment(reference='Pseudo-nitzschia'))[T.Prorocentrum Cordatum]	-1.7781	0.571	-3.116	0.002	-2.896	-0.660
C(Species, Treatment(reference='Pseudo-nitzschia'))[T.Prorocentrum lima]	-1.7390	0.518	-3.355	0.001	-2.755	-0.723
C(Region, Treatment(reference='Mainland'))[T.IoS]	0.3335	0.169	1.979	0.048	0.003	0.664
C(Species, Treatment(reference='Pseudo-nitzschia'))[Alexandrium]:C(Region)[T.Mainland]	1.1664	0.366	3.189	0.001	0.449	1.883
C(Species, Treatment(reference='Pseudo-nitzschia'))[Dinophysieae]:C(Region)[T.Mainland]	0.1640	0.273	0.600	0.549	-0.372	0.700
C(Species, Treatment(reference='Pseudo-nitzschia'))[Prorocentrum Cordatum]:C(Region)[T.Mainland]	-0.2199	0.445	-0.494	0.621	-1.092	0.652
C(Species, Treatment(reference='Pseudo-nitzschia'))[Prorocentrum lima]:C(Region)[T.Mainland]	0	0	nan	nan	0	0
C(Species, Treatment(reference='Pseudo-nitzschia'))[Pseudo-nitzschia]:C(Region)[T.Mainland]	4.0218	0.271	14.842	0.000	3.491	4.553
Date_Num	0.0152	0.001	13.263	0.000	0.013	0.017

Figure S10. Binomial Generalised Linear model outputs for the effect of region on concentrations of PSTs, DA and Total OA group

```
=== Total PST (µg STX eq/kg)
===
                 Generalized Linear Model Regression Results
==============================================================================
Dep. Variable:                  Count   No. Observations:                    6
Model:                            GLM   Df Residuals:                        4
Model Family:                   Gamma   Df Model:                            1
Link Function:                    log   Scale:                        0.020775
Method:                          IRLS   Log-Likelihood:                -16.197
Date:                Thu, 02 Oct 2025   Deviance:                     0.083147
Time:                        15:53:35   Pearson chi2:                   0.0831
No. Iterations:                    11   Pseudo R-squ. (CS):              1.000
Covariance Type:            nonrobust
======================================================================================
                         coef    std err          z      P>|z|      [0.025      0.975]
--------------------------------------------------------------------------------------
Intercept              1.2809      0.072     17.774      0.000       1.140       1.422
Region[T.Mainland]     6.3250      0.125     50.671      0.000       6.080       6.570
======================================================================================
Likelihood ratio test for Region effect: LR=42.071, p=0.0000 -> ✅ Statistically different
between regions
```

```
=== DA (mg/kg) ===
                 Generalized Linear Model Regression Results
==============================================================================
Dep. Variable:                  Count   No. Observations:                   17
Model:                            GLM   Df Residuals:                       15
Model Family:                   Gamma   Df Model:                            1
Link Function:                    log   Scale:                         0.22795
Method:                          IRLS   Log-Likelihood:                -4.7933
Date:                Thu, 02 Oct 2025   Deviance:                       2.8335
Time:                        15:53:35   Pearson chi2:                     3.42
No. Iterations:                     6   Pseudo R-squ. (CS):             0.4537
Covariance Type:            nonrobust
======================================================================================
                         coef    std err          z      P>|z|      [0.025      0.975]
--------------------------------------------------------------------------------------
Intercept             -0.5062      0.151     -3.353      0.001      -0.802      -0.210
Region[T.Mainland]     0.7464      0.235      3.172      0.002       0.285       1.208
======================================================================================
Likelihood ratio test for Region effect: LR=9.687, p=0.0019 -> ✅ Statistically different
between regions
```

```
=== Total OA-group (µg OA eq/kg) ===
                 Generalized Linear Model Regression Results
==============================================================================
Dep. Variable:                  Count   No. Observations:                   73
Model:                            GLM   Df Residuals:                       71
Model Family:                   Gamma   Df Model:                            1
Link Function:                    log   Scale:                         0.62795
Method:                          IRLS   Log-Likelihood:                -311.05
Date:                Thu, 02 Oct 2025   Deviance:                       33.729
Time:                        15:53:35   Pearson chi2:                     44.6
No. Iterations:                     7   Pseudo R-squ. (CS):             0.4262
Covariance Type:            nonrobust
======================================================================================
                         coef    std err          z      P>|z|      [0.025      0.975]
--------------------------------------------------------------------------------------
Intercept              2.7475      0.134     20.512      0.000       2.485       3.010
Region[T.Mainland]     1.2268      0.186      6.608      0.000       0.863       1.591
======================================================================================
Likelihood ratio test for Region effect: LR=42.273, p=0.0000 -> ☑ Statistically different
between regions
```

Figure S11. Figures showing toxin profiles across all shellfish samples in terms of a) µg/L for OA, DTX1, AZA1 and AZA2 b) proportions of OA and DTX1 in terms of OA eq/kg and c) proportions of AZA1 eq/kg for AZA1 and AZA2

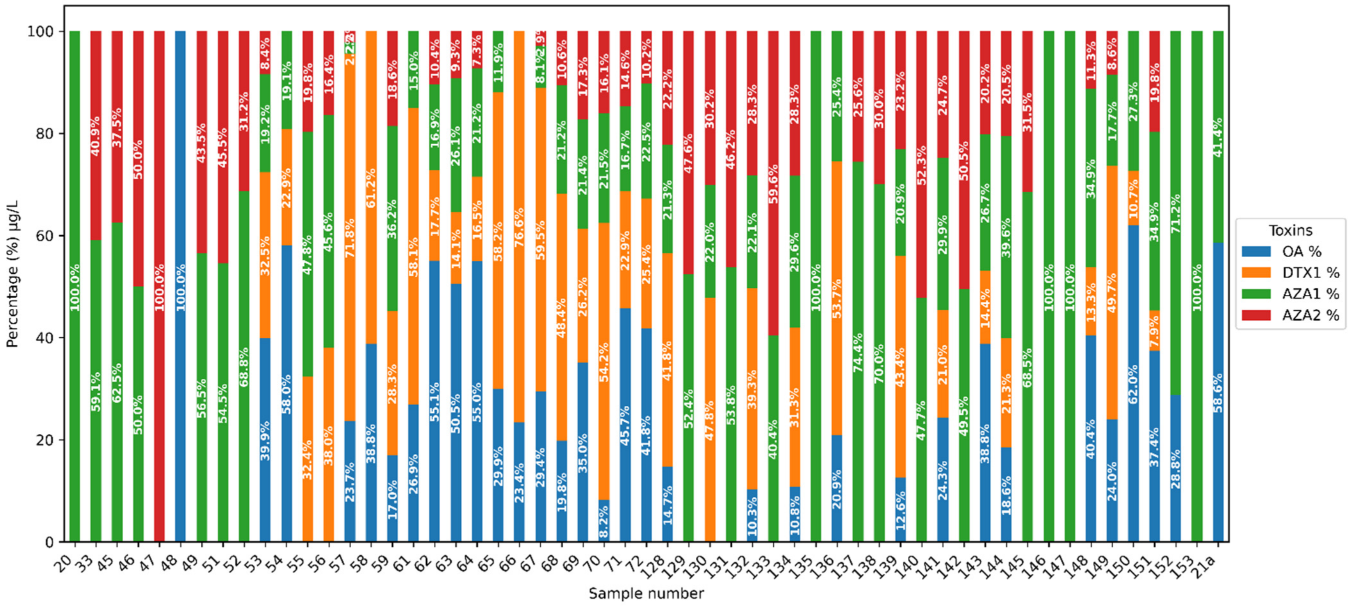

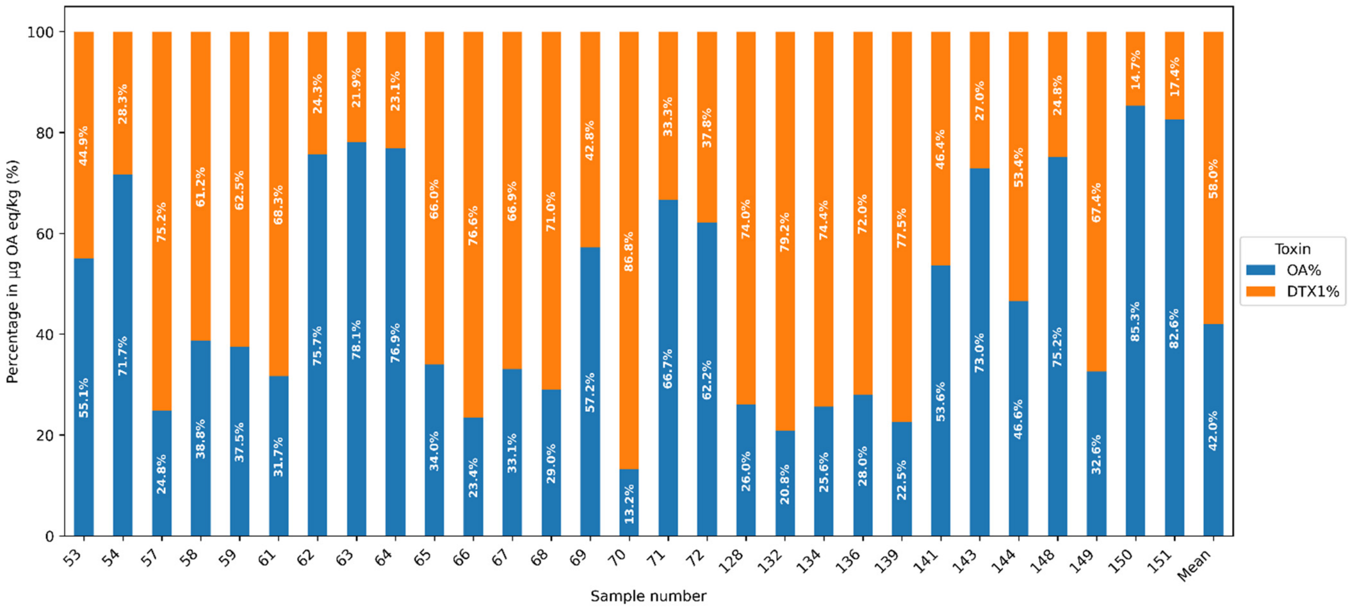
Percentage in µg OA eq/kg (%)
100
80
60
40
20
0
Sample number
53 54 57 58 59 61 62 63 64 65 66 67 68 69 70 71 72 128 132 134 136 139 141 143 144 148 149 150 151 Mean
Toxin
OA%
DTX1%
55.1% 71.7% 24.8% 38.8% 37.5% 31.7% 75.7% 78.1% 76.9% 34.0% 23.4% 33.1% 29.0% 57.2% 13.2% 66.7% 62.2% 26.0% 20.8% 25.6% 28.0% 22.5% 53.6% 73.0% 46.6% 75.2% 32.6% 85.3% 82.6% 42.0%
44.9% 28.3% 75.2% 61.2% 62.5% 68.3% 24.3% 21.9% 23.1% 66.0% 76.6% 66.9% 71.0% 42.8% 86.8% 33.3% 37.8% 74.0% 79.2% 74.4% 72.0% 77.5% 46.4% 27.0% 53.4% 24.8% 67.4% 14.7% 17.4% 58.0%

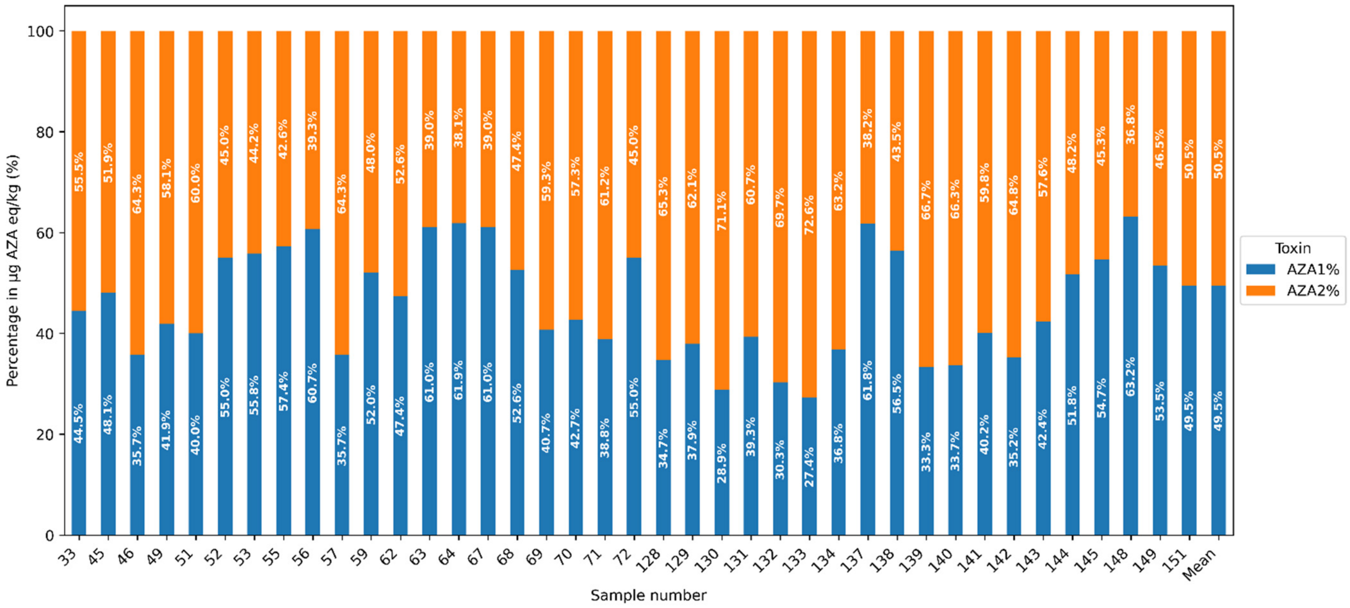
Percentage in µg AZA eq/kg (%)
100
80
60
40
20
0
Sample number
33 45 46 49 51 52 53 55 56 57 59 62 63 64 67 68 69 70 71 72 128 129 130 131 132 133 134 137 138 139 140 141 142 143 144 145 148 149 151 Mean
Toxin
AZA1%
AZA2%
44.5% 48.1% 35.7% 41.9% 40.0% 55.0% 55.8% 57.4% 60.7% 35.7% 52.0% 47.4% 61.0% 61.9% 61.0% 52.6% 40.7% 42.7% 38.8% 55.0% 34.7% 37.9% 28.9% 39.3% 30.3% 27.4% 36.8% 61.8% 56.5% 33.3% 33.7% 40.2% 35.2% 42.4% 51.8% 54.7% 63.2% 53.5% 49.5% 49.5%
55.5% 51.9% 64.3% 58.1% 60.0% 45.0% 44.2% 42.6% 39.3% 64.3% 48.0% 52.6% 39.0% 38.1% 39.0% 47.4% 59.3% 57.3% 61.2% 45.0% 65.3% 62.1% 71.1% 60.7% 69.7% 72.6% 63.2% 38.2% 43.5% 66.7% 66.3% 59.8% 64.8% 57.6% 48.2% 45.3% 36.8% 46.5% 50.5% 50.5%

Data S1: Consensus sequences generated for potential HAB genera referenced in table 2.

>Sequence_1_Alexandrium_consensus_cl_id_227_total_supporting_reads_46
ATATTACTTCGTTCAGTTACGTATTGCTGAACTTGTACAAGCATCCCAACACTTCCACTT
TAACCTACTTGCCTGTCGCTCTATCTTCCCTTGGTCCGTGTTTCAAGACGGGTCGAAGAG
AACCATTTTGTCCACCACTGTATGTCAAGATAACAAGCGCATGTAGCCTAATATGCAACC
CAAAGAGAGCAAATCAGACATCAATGATAGCACCAGACATCCGAAACCTGTTCACGTTTG
AATCTGCACCATACTTCGTTCAGTTACGTATTGCTGGTGCTGGAAACTTGGATTGTGTCA
GTATTAACCTACTTGCCTGTCGCTCTATCTTCCCTTGGTCCGTGTTTTAAGCGAGTGACC
ATTAATGAGACATGCATGGCTTAATCTTTGAGACAAGCGCAATATCAGCACCAACAGAAA
GGTTAATGGTAACTGTGAAGTGTTGGGATGCTTTACAGCACCCGTGCTGGACCATTGTGA
TGAACCCTGTTGTTTAACCTTTCTGTTGGTGCTGATATTGCGCTTGTCTCAAAGATTAAG
CCATGCATGTCTCAGTATAAGCTTTCATTTTGTGAAACTGCAAATGGCTCATTAAAACAG
TTATAATGTATTTGACAATCAACTCTATATGGATATCTGTGGAAATTCTATAGTTAATAC
ATGCATTGAAACCGATCTTTGGGGAAAGGTTGTGGTCGTTAGTTACAGAACCAATTCAAG
CATTGCTTGAATACTTGATTGATTCATAATGACAAAATGAATTACATGGCAACAGCTGGT
AATAATTCATTCCAGTTTCTGACCTATCAGCTTTCGACGGTAAGGTATTGGCTTACCGTG
GCAATGACAGGTGACGGAGGATTAGGGTTTGATTCCGGAGAGGGAGCTTGAGAAATGGCT
ACCACATCTAAGGAAGGCAGCAGGCGCGCAAATTACCCAATCCTGGCACAGGGAGGTAGT
GACAAGAAATAACAATACAAGGCATCCATGTCTTGTACTTGGAATGAATGGATTTTAAAC
CTTTCTGTAAGTATCAATTGGAGGGCAAGTCTGGTGCCAGCAGCCGCGGTAATTCCAGCT
CCAATAGCGTATATTAAAGTTGTTGCGGTTAAAAAGCTCGTAGTTGGATTTCTGCTGAGG
ATGGCTGGTCCGCCCTCTGGGTGAGTATTTGGCACAGCCTGAGCATTTATCTTGAAAGCA
CAACTGCACTTGACTGTGTGGTGTGTTATTGAGAACGTTTACTTTGAGGAAATCAGAGTG
TTTCAAGCAGGTGTTTGGCCTTGAATACATTAGCATGGAATAATAATCAAGATCGTGATT
CTTTTTTGTTGGTTTCTAGAATTGAGGTAATGATTAATAGGGATAGTTGGGGGCATTCGT
ATTTGATCGTCAGAGGTGAAATTCTTGGATTTGTTAAAGACGGACTACTGCGAAAGCATT
TGCCAAGGATGTTTTCATTGATCAAGAACGAAAGTTAAGGGATCGAAGACGATCAGATAC
CGTCCTAGTCTTAACCATAAACCATGCCAACTAGAGATTGAAGGTTGTTACTTGTATGAC
TTTTTCAGCACCCTATGAGAAATCAAAGTGTTTGGGTTCCGGGGGGAGTATGGTCGCAAG
GCTGAAACTTAAAGGAATTGACGGAAGGGCACCACCAGGAGTGGAGCCTGCGGCTTAATT
TGACTCAACACGGGGAAACTTACCAGGTCCAGACATAATGAGGATTGACAGATTGATAGC
TTTTTCTTGATTCTATGGGTGGTGGTGCATGGCCGTTCTTAGTTGGTGGAGTGATTTGTC
TGGTTAATTCCGTTAACGAACGAGACCTTAACCTGCTAAATAGTTACATGTAATTTCGAT
TATGTGGGCAGCTTCTTAGAGGGACTTTGTGTGCATAATGCAAGGAAGTTTGAGGCAATA
ACAGGTCTGTGATGCCCTTAGATGTTCTGGGCTGCACGCGCGCTACACTGATGTGTTCAA
CGAGTTTTCAACCTTGCCTGGAAAGGTTTGGTAATCTTGAACAGGCATCGTGATGGGGAT
TGTTTATTGCAATTATTAACCTTCAACGAGGAATTCCTAGTAAGCTTGAGTCATCAGCTT
GTGCTGATTATGTCCCTGCCCTTTGTACACACCGCCCGTCGCTCCTACCGATTGAGTGAT
CCGGTGAATAATTTGGACTGTAGCAATGTTCAGTTCTTGAACAACGCAATGGAAAGTTTA
ATGAACCTTATCACTTAGAGGAAGGAGAAGTCGTAACAAGGTTTCTGTAGGTGAACCTGC
GGAAGGATCATTTGCACATGTGTATCCAACTTCATTCTAATGATATTGTGGGCAAGTGCG
GGCATGTATTGCAATGTGCTTGCATGTGCCCTGGGCTGCATGACTTGTTTTACAATCATG
TGTGCTGCACTATCTAATATACTTAATCAACTGTTTGGTAATTCTTCATTGATTACAATG
ATTATGTTTTGCAAAGAATGTATTAGTTCAATAAATGATGAAGAATGCAGCAAAATGCAC
TATGCATTGTGAATTGCAGAATTCCGTGAGCTAACAGATGTTTGAATGTTACTTGTACCT
TTGGGATATTCTTGAAGGTGTGCTTGATTCAATGCAAACTGTCTTCCATATGCAATAATG
CTGCTTAGCATTGCTGTGAACAGTAGGGGTCAATGTGTGTGCATTGAACCTGGGTGTTGT
GCAGCTGTTTGCAACCTAAACATGTTTTCTTGGGGGCAAACTTGTTTCGTCATTTGCTGG
TTGATATGTGTAAAAATGCATATTTGAAACAAGTTGAATACTTGCATTCACTTAGCGTGA
AGTGAAGCACATAAACCTGCTGAATTTAAGCATATAAGTAAGTGGTGGAAATTAAACCAA
ATGGGATTTCTTTAGTAATTGCGAATGAACAAGGATATGCTTAGCTTGACAAATGGAGCT
TTTGGCTTTGAGTTGTATTGTGGAAATGTACCACCAACAGAGGTGCAAGTGTCAGCCATT
TGAAAGAAAGCGTCAATGAGGGTGAGAATCCTGTTTGTCATGTGCAGCCCTCTGTGCACG
GTGTATATTTGCTGAGTCACACTCCTTGGCATTGGAATGCAAAGTGGGTGGTAAGTTTCA
TACAAAGGTAAATATGCAATTGAGACTGATAGCACACAAGTACCATGAGGGAAATATGAA
AAGGACTTTGAAAAGAGAATTAAATGAGTTTGTATTTGCTGAACACAAAGCAAACAGACT
TGATTTGCTTGGTGAGATTGTAGTGCTTGCTTGACAATAGGTTTTGGCTGTGGGTGAAAT
GATTCTTTCTTTGCATGCCAGGTTCTATGTGTACATTTGATTACCTTTGCACATGAATGG
TAATTTTCCTGCGGGGGTGGATTGCATGTGCATGTAATGATTTGTGTTTTGATGAATGTG
TCTGGTGTATCTGTTTTTGTTCCATGTGTTTGAGGTTGCTTCCTTCCTTGGGCTTACAAG
CCCTGACACACACAATCTGGCAAAATGTTTCTGCTTGACCCGTCTTGAAACACGGACCAA
GGGGAAGATAGAGCGACAGGCAAGTAGCAATACAACAGGGTTCATCACAATGGTCCAGCA
CCAGGTGCTGAAACGGGATGACGGGAGCCGCCCAGCCTGCGCGCGCGTGCAAATAATGTT
CAAGCAGGGACCAAATGGTGCAGTGCATGGCAATCTTTGGAACCGGCGCAATATCAGCAC
CAACAAGGAAGGTTAGGGTTCTGTCTGTCTGTTTCTTCAGCATAACCATGTA

>Sequence_2_Azadinium_consensus_cl_id_37_total_supporting_reads_24
GCTAGTTACGTATTGCTGGTGCTGGAGTCTTGTGTCCCAGTTACCAGGTTAACCTTTCTG
TTGGTGCTGATATTGCGCTTGTCTCAAAGATTAAGCCATGCATGTCTCAGTATAAGCTTC
TACACGGCGAAACTGCGAATGGCTCATTAAAACAGTTATAGTTTATTTGATGGTCACTCT
TACATGGATAACCGTGGTAATTCTAGAGCTAATACATGCGCCCATACCCGACTTCGTGGA
AGGGTTGTGTTTATTAGTTACAGAACCAACCCAGGCTTCTGTCTGGTCTTGTGGTGATTC
ATAATAACCGAACGAATCGCATAGCAACAGCTGGCGATGAATCATTCAAGTTTCTGACCT
ATCAGCTTCCGACGGTAGGGTATTGGCCTACCGTGGCAATGACGGGTAACGGAGAATTAG
GTTCGATTCCGGAGAGGGAGCCTGAGAAACGGCTACCACATCTAAGGAAGGCAGCAGGCG
CGCAAATTACCCAATCCTGACACAGGGAGGTAGTGACAAGAAATAACAATACAGGGCATC
CATGTCTTGTAATTGGAATGAGTAGAATTTAAATCCCTTTGCGAGTATCGATTGAGGGCA
AGTCTGGTGCCAGCAGCCGCGGTAATTCCAGCTCCAATAGCGTATATTAAAGTTGTTGCG
GTTAAAAAGCTCGTAGTTGGATTTCTGCCGAGGACGACCGGTCCGCCCTCTGGGTGAGTA
TCTGGCTCGGCCTGGGCATCTTCTTGGAGAACGTAGCTGCACTTGACTGTGTGGTGCGGT
ATCCAGGACATTTACTTTGGAGGAAATTAGAGTGTTTCAAGCAGGCACACGCCTTGAATA
CATTAGCATGGAATAATAAGATAGGACCTCGGTTCTATTTTGTTGGTTTCTAGAGCTGAG
GTAATGATTAATAGGGATAGTTGGGGGCATTCGTATTTAACTGTCAGAGGTGAAATTCTT

GGATTTGTTAAAGACGGACTACTGCGAAAGCATTTGCCAAGGATGTTTTCATTGATCAAG
AACGAAAGTTAGGGGGATCGAAGACGATCAGATACCGTCCTAGTCTTAACCATAAACCAT
GCCGACTAGAGATTGGAGGTCGTTACCTATACGACTCCTTCAGCACCTTATGAGAAATCA
AAGTCTTTGGGTTCCGGGGGAGTATGGTCGCAAGGCTGAAACTTAAAGGAATTGACGGAA
GGGCACCACCAGGAGTGGAGCCTGCGGCTTAATTTGACTCAACACGGGGAAACTTACCAG
GTCCAGACATAGTAAGGATTGACAGATTGATAGCTCTTTCTTGATTCTATGGGTGGTGGT
GCATGGCCGTTCTTAGTTGGTGGAGTGATTTGTCTGGTTAATTCCGTTAACGAACGAGAC
CTTAACCTGCTAATAGTTACACGTAACCTCGGTTACGTGGGCAACTTCTTAGAGGGACTT
TGCGTGTCTAACGCAAGGAAGTTTGAGGCAATAACAGGTCTGTGATGCCCTTAGATGTTC
TGGGCTGCACGCGCGCTACACTGATGCGCTCAACGAGTTTACGACCTTGTCCGGAAGGAT
TGGGTAATCTTTTTTAAAACGCATCGTGATGGGGATAGATTATTGCAATTATTAATCTTC
AACGAGGAATTCCTAGTAAGCGCGAGTCATCAGCTCGTGCTGATTACGTCCCTGCCCTTT
GTACACACCGCCCGTCGCTCCTACCGATTGAGTGATCCGGTGAATAATTCGACTGCAGCA
GTGTTCAGTTCCTGGACGTTGCAGCGGAAAGTTTCGTGAACCTTATCACTTAGAGGAAGG
AGAAGTCGTAACAAGGTTTCCGTAGGTGAACCTGCGGAAGGATCATTCGCACGCATCCAA
CATGAACCATGTGCATTTGCTTCGTGAGGTTCTGAGCGGGAGCAATTACCCACTCAGAAA
CTCGAGGGCAGCAGTGTGGGGTTGGATTCACAGCCCCATTCCTGCTGTCGTGTTCCTCTT
GAACCTTTACATTGACTTGCCATGCCTTCTCGAGTGGCTATCCACTCAAACGAAAGTTAC
AACTTTCAGCGACGGATGTCTCGGCTCGAACAACGATGAAGGGCGCAGCGAAGTGTGATA
AACATTGTGAATTGCAGAATTCCGTGAACCAATAGGGAATTGAACGTATACTGCGCTCGG
GATATCCCTGAGAGCATGCCTTCTTCAGGGCCACTACCTTCTCATGCCAGTAGCTTCACA
ACCCCTTGCGGGTTAGCTGCTTGTGCGTGTTTGTGCGTCAAGTTGCATCCTGCGCCTGAC
GCATTGAGCTCATGAGAGCCTCTGCACCAATCAACTTATTGAGTGTCTCCTGGCGCCATC
TGTTGCGTTGTGTGCAAAGTGCTGCTCGATCACTAGGTGCTTTTTCAAGCCCTCTCCCAT
GGCATGAAGTGAGGTAAGAAAACCCGCTGAATTTAAGCATATAAGTAAGCGGAGGACAAG
AAACTAAATAGGATTCCCTTAGTAATGGCGAATGAACAGGGAACAGCTCAGCATGGAAAT
CGGGGCCTCCCGGCCTTGAATTGTAGTCTGGAGATGCTGCCAACGGAGGCGCAGATGTAA
GCCTCTGGAAAAGAGCATCAACGAGGGTGAGAGTCCTGTTTGTCATCTGCAGTCCCCGTG
CACGGTGCGCGTTTGACGAGTCACGTTCCTCGGGATTGGAGCGCAAAGTGGGTGGTAAAT
TTCATCTAAAGCTAAATACTGGTTCGAGACCGATAGCAAACAAGTACCATGAGGGAAAGG
TGAAAAGGACTTTGAAAAGAGAGTTAAAAGTGCCTGAAATCGCTGAAAGGGAAGCGAATG
GAACCAGTGTTCTTGGTGAGATTGTTGCGCACTCTGGGGATTGCTGGCTGTTCAACCGCA
AGGGGAGCAGTTGGTTTTGACTTAGCGGTGCGTGGTGTTTCTTGCCCTGTGTGTCACCGT
CGGTGCACCAGCGAGTAAACCACCAAGGGCATGGTACCTCACCCACGGGTGGGCGAATAC
CTTTGGCTGCACTCATTGGTGTATCGATCCCTGCGTGTCTGGTTGCAGTGCTCAGTTCGT
CTCAGTGCTTCTGCGGGGTTCGTCTCTGTGGGAGTTTCCTGGGGCGCGGGGGCATCTCCC
TGACACAAAGACGATGACTAAATGGTTCTATTCGACCCGTCTTGAAACACGGACCAAGGG
AAGATAGAGCGACAGGCAGTAGGTTAACCTGGTAACTGGGACACAAGACTCCAGCACCAG
CAATACGTAACTGAACGAAGTACAATG

>Sequence_3_Coolia_consensus_cl_id_3_total_supporting_reads_22
TTGTTGCGCTTGTCTCAAAGATTAAGCCATGCATGTCTCAGTATAAGTTCATGATTGGCA
AAACTGCAAATGGCTCATTAAAACAATTACAATGCATTTGGATATGTTTTACATGGTTAA
TTTTGGTAATTCTAAAACTAATACATGCGCACAAACTTGACTTGTCGGAATGGTTGTTGT
CATTAGATACAAAACCATCACCAGCTTCATGCTGGAACTAGGTGAATCACAATGACAAAA
TGAATTACATGGCTTCTGTTGGTGATATTCCATGGCAGTTTCTGACCTATCAGCTTCCGA
TGGTATGGTCTTGGCATACCGTGGCGTTGACGGGTGACGGAGGATTAGGGTTTGATTCCG
GAGAGGGAGCTTGAGAAATGGCTACCACATCTAAGGAAGGCAGCAGGCGCGCAAATTACC
CAATCTTGACAAAGGGAGGTAGTGACAAGAAATAACAATATAAGGCATCCATGTCTTGTA
TTTGGAATGAAATGACAACAAACCTTTCTATGAGTATCAATTGGAGGGCAAGTCTGGTGC
CAGCAGCCGCGGTAATTCCAGCTCCAATGGCGTATATTAAAATTGTTGCGGTTAAAAAGC
TCGTAGTTGAATTTCTGTTGAGGGTGGTTGGTCCGCCCTCTGGGTGAGTATCTGACACAA
CCTGAACATGTTCTTGATTGCACAACTGTACTTGACTGTGTGGGGTGGTATTGAGAAGGT
TTACTTTGAAGAAATCAGCGTGTTTCAAGCAAGTGTTGCTTTGAATACATTAGCATGGAA
TAATGTTCAAGGACTTTGGTTTCATTTTGTTGGTTTCTGGAATCTGGGTAATGACAAATA
GGGATAGTTGGTGGTATTTGTATTTAATTGTCAGAGGTGAAATTCTTGGATTTATTAAAG
ACAAACTATTGCGAAAGCATTTGCCAAGGATGTTTTCATTGATCAAGAACGAAAGTTAGG
GGATCGAAGACGATCAGATACCGTCCTAGTCTTAACCATAAACTATGCCAACTAAAGATT
GAAAGTTGTTATTTGCATGACATTTTCAGCACCTTATGAAAAATTGAAGTCTTTGGGTTC
CGGGGGAGTATGGTCGCAAGGCTGAAACTTAAAGGAATTGACGGAAGGGCACCACCAGGA
GTGGAGCCTGCGGCTTAATTTGATTCAACACGGGGAAACTTACCAGGTCCAGACATAATG
AGGATTGACAGATTGATAGCTTTTTCTTGATTCTATGGGTGGTGGTGCATGGCCGTTCTT
AGTTGGTGGAATGATTTGTCTGGTTAATTCCGTTAACGAACGAGACCTTATTCTGCTAAA
TCATTACATGTAATTTTTGTTATGTGGGCAACTTCTTAGAGAGACTTTGTGTGCATAACA
CAAGGAAGTTTGAGGCAATAACAGGTCTGTGATGCCCTTAGATGTTCTGGGCTGCACGCG
CGCTACACTGATGTGTTCAACAAGTTTGCATCCTTACTTGGAAAGGTTGGGTAATCTTGT
AGATTGCATCGTGATGGGGATCGATTATTGCAATTATTAATCTTGAACGAGGAATCCCTA
GTAAACATAAATCATCAGTTTGTGTTGATTACGTCCCTGCCCTTTGTACACACCGCCCGT
CGCTCCTACCGATTGAGTGATCCGGTGAATAATTTGGACTGAAACATTGTTCAGTTCTTG
AATAATGTTATGGAAAATTTAATGAACCTTATCACTTAGAGGAAGGAGAAGTCGTAACAA
GGTTTCTGTAGGTGAACCTGCGGAAGGATCATTTGTGTGTCTTGCAATCATTGTTTTTTG
ATTGAATAGCAGGATGGCAACATCACCATGTGTGTGTTGTCATCATTTTCAACAACCATA
ATTGTGTCATTGTATGCTGTGTGTGTAGTTTGGCAATAAATGTATAAGTTCAACATGTGA
TGAAGAATGTAGCTAAATGCAATAATATTTGTGAAATGCAGAATCCCGTGAATCAATTCA
TGTTTGAATGTGTCTTGCATCTTTGGGATATGCTTGAAGATATGTGTGATTCAATGTCAA
ATGTGTGTGCATGGATGCATGTAATGTGTTTGTATTGATTTTTTTACACATTAATGATAT
GTTTGAAAATTTTGAAAACACCCATGACATGTATCATGAACAGACAAATACAAACACATG
AATTGTGCAAGCAAACCTGCTGAATTTAAGCATATAAGTAAGTGGTGGAAGAAAACATAA
ACATGATTCTTTAAGTAATGGCGAATGAACAAAGACATAGTCATTTTGAAAATTGTGGTC
CGTGATCATGAATTGTAATATTCAAAATGCATTGCTAACAAAATTGCATATGTTAGTTTA
TTGGAAAGAACATTACAAAGGGTGAAAATCCCATGTGTCATGTGTATGTTTTGTGTACAG
TGTGCATTTCATGATTCACATTCATTGACAATTGAATGTAAAGCGAATGGTAAACTTCAT
TTAAACATAAATATGTTGTTGAAAACTTATATCGAACAAATACCATGAGGGAAATGTGAA
AAGGATTTTGAAAAGAAAATTAAAAGAGCTTACAATTGCTGGATTGATAAGCAAACAAAA
CACACATGTGTATGTGCAAGATTGTTGTCATGTTGTGCAATTCATGCAAATTAACATTGC

TTTGGTTTTTGTGTAATTTTGTCAGCTTTGGAATTGAGAATGATTTTTTTGAGTGTGAAT
GGATTGTTGTTTGTTAACAAAGGAATGCACTTTCAAAATCATTGTTTGATTTGTCGATCA
AGTTTGTGGCGTGTGTTGTGTGTCAAATTTAATTATATAATTCACAAAACACATGAAGTT
TGAGCTATTAAGGCAATTCTTGTTTTGTTTGACCCGTCTTGAAACACGGACCAAGGGAAG
ATAGGGGGCGACAGTGCAAGTAGGTTAACTGCTTCCGGATCAGTGGTA

>Sequence_4a_Dictyocha_consensus_cl_id_3_total_supporting_reads_178
GCGCGGCAAGTATTGTACTTCGTTCAGTTACGTATTGCTGGTGCTGAACTAGGCACAGCG
AGTCTTGGTTTTAACCTTTCTGTTGGTGCTGATATTGCGCTTGTCTCAAAGATTAAGCCA
TGCATGTCTAAGTATAAACGACTTTATACTGTGAAACTGCGTACAGCTCATTAAACCAGT
TATAGTTTATTTGATATTCCTCTACTACTCGGATACCCGTAGTAATTCTAGAGCTAATAC
GTGCATCAAATCCCAACTGCTTCGGCGGACGGGACGTCATTATTAGAAAGAAGCCAATGC
GACGCAAGTCGGTACTGTGGTGAGTCATGATAATTGTACGGATCGTATGGCCTTGCGCTG
ACGATGGATCATTCAAGTTTCTGCCCTATCAGCTTTGGATGGTAGGGTATTGGCCTACCA
TGGCTTTAACGGGTAACGGAGAATTAGGGTTCGATTCCGGAGAGGGAGCCTGAGAGACGG
CTACCACATCCAAGGAAGGCAGCAGGCGCGTAAATTACCCAATCCTGATACAGGGAGGTA
GTGACAAAAAATAACAATGCCGGGCTTTTTTAAGTCTGGCAATTGGAATGAGAACAATTT
AAATCCCTTATCGAGGATCAATTGGAGGGCAAGTCTGGTGCCAGCAGCCGCGGTAATTCC
AGCTCCAATAGCGTATATTAATGTTGTTGCAGTTAAAAAGCTCGTAGTTGGATTTCTGAT
GGGATCGACCCGGTCGGCTCCGAAAGGGGTCGCACTGGTGGTCTGTCCGGTCATCCTCGA
GGGGAGGCTTTCTGGCATTCATTTGTTGGGGAGTCGACGCTCGTCGTTTACTGTGAACAA
ATTAGAGTGTTCAAAGCAGGCTTAGGCCGTTGAATACATTAGCATGGAATAATAAGATAG
GACTTTGGTGGTTTATTTTGTTGGTTTGCACGCCGAAGTAATGATTAATAGGAGCAGTTG
GGGATGTCCGTATTCGATTGTCAGAGGTGAAATTCTTGGATTTATTGAAGACGAACTACT
GCGAAAGCATTCATCAAGGATGTTTTCATTAATCAAGAACGAAAGTTAGGGGATCGAAGA
AGATTAGATACCTTCGTAGTCTTATACCATAAACTATGCCGACTAGGGATTGGCGGTCGC
TTGTTCAGGCTCCGTCAGCACCTTATGAGAAATCAAAGTCTTTGGGTTCCGGGGGGAGTA
TGGTCGCAAGGCTGAAACTTAAAGAAATTGACGGAAGGGCACCACCAGGAGTGGAGCTTG
CGGCTTAATTTGACTCAACACGGGAAAACTTACCAGGTCCAGACATAGTAAGGATTGACA
GATTGAGAGCTCTTTCTTGATTCTATGGGTGGTGGTGCATGGCCGTTCTTAGTTGGTGGA
GTGATTTGTCTGGTTAATTCCGTTAACGAACGAGACCCCCGCCTGCTAAATAGTGACGGG
AATGCTTTGCATTGCTGGATCTACTTCTTAGAGGGACTTTCGGCGACTAGCCGAAGGAAG
TTGGGGGCAATAACAGGTCTGTGATGCCCTTAGATGTCCTGGGCTGCACGCGCGCTACAC
TGATGCATGCAACGAGTTTAGAACCTTGGCTGAGAGGCCTGGGTAATCTGTGAACGTGCA
TCGTGATAGGGATTGATGATTGCAACTATTCATCATGAACGAGGAATTCCTAGTAAACGT
GAGTCATCAGCTCACATTGATTACGTCCCTGCCCTTTGTACACACCGCCCGTCGCACCTA
CCGATTGAATGGCTCGGTGAGGCCTCAGGATTCTGGTCTGACACCCTAACAGGAGTCTGA
TGAGAAGAATCTGTCCAAACCTCGTCATTTAGAGGAAGGTGAAGTCGTAACAAGGTTACC
GTAGGTGAACCTGCGGTAGGATCATTCTATCACTATAATCCATATCAACACCGTGAACAC
CCCAAAATCTCAGGATTTCGGCGGACGAGATTGCATGCTGCGGCATACTGTTTCGGTCGT
TGTTTTCCGCAAATCTATAAACAACAAAACAACAACTTGCCTCCATTGCGAGACTTCGAA
AAATAATGAACTGCTTTTCGACCGACTTGCTCGGTAGGACAGCAGCTATTACAAATACCA
TTATAACTTTCAGCGACGGATGTCTTGGCTCCCATAACGATGAAGAACGCAGCGAAATGC
GATACGTAATGCGAATTGCAGAATCCAGTGAATCATCAAAACTTTGAACGCATCTTGCGC
TTCCGGGATATGCCTGGGAGCATGCTCAGTGGAGTGTCTGCTAACACAACCCTCATCCCT
CCCTGTGTGAAACTTGTTTTGCATGGAGAGGCGACGGACTCTGGGTGTCGCACCGTGTAA
CAACGGAGCGTCCCCCGAAAAGCAGACCCCATTGCGTGATACTCAATCGAAGCCTTGTGT
TTCGTATCCGCTGACGGTGTGGTAGGCATTCTAAACACTTGCTTATACTAAATCACCATT
AGTTGGGACGGAAGCTATGCTCGGCATTGAATGAGTGTCCGGGTCAGAACCCCGAGGAAA
CTTGACTCCGCCACATCGGCGGAGCTGGGAATCCCCACTAGTTCATTCAACGGAGAGGGC
GCAAGCCTTCCCGGAGAAGAATTATCCTGACCTCCTCTGAGCAAGAGGACCGGCTGAATT
TAAGCATATTACTAAGCCGAGGAAAAGAAACTAACTAGGATTCCCTCAGTAAGGGCGACT
GAAGCGGGAATAGCCCATGCTGCCAATCGCTTCGGCGAGTTGTGTCAGGCGTGCCTGGCA
GACCTCCGGGGAGAAGTCCCTTGGAACAGGGTATCAGAGAGGGTGACAGTCCCGTCTGTG
CCTCGGAGGCTCTGTGTGAAGGCCGCATTACTAGAGTCGAGTTGCTTGGGATTGCAGCTC
AAAGTTGGTGGTAAATTCCATCTAAGGCTAAACACGGGTGGGAGACCGATAGCGAACAAG
TACTGTGAAGGAAAGATGAAAAGAACTTTGAAAAGAGAGTCAAAGAGTGCCTGAAATTGC
TGAGAGGGAACCGTAGGTAACCAATGCGAGCCTTGATCATCTTCCGGCGGTCCTCTGGGA
CCGCCGCCCTGTGGTCTTGGCCATCCCAGCCTTTTCATGTTCCGGAGGAAACCATTCGAT
TTATCGGGTAGAGGCTCTGGGGGATGCGAAGGTTTGGTAAATGGTTCTCTACACCCCGTC
TTGAAACACGGACCAAGGGAAGATAGAGCGACAGGCAAGTAGGTTAAAACCAAGACTCGC
TGTGCCTAGTTCAGCACCAGCAATACGTAACTTA

>Sequence_4b_Aureococcus_cl_id_14_total_supporting_reads_574
AAACATTGTACTTCGTTCAGTTACGTATTGCTGGTGCTGGAGTCTTGTGTCCCAGTTACC
AGTTTAACCTACTTGCCTGTCGCTCTATCTTCCCTTGGTCCGTGTTTCAAGACGGGGTGA
ACGGAAGAATTTCGTCACCGAAGGTCTCAGGCGCGGCCTGAACACGGGAGATTGGTCCGA
AGACCTTCCCATGAACGACTCCCACGCCCCAACCCACGCTGACCTACGGACGCGCACAAG
TACGCCGGCCAAGAGCCCGGCGGAAAATACGCGCCCATCACCACTTCTGCCGTTCGGTTC
CCTCTTACCAGTTTCAGGCCCTTTTTAACTCTCTTTTCAAAGTTCTTTGCATCTTTCCCT
CACGGTACTTGTTCGCTATCGGTCTCCCACCCGTATTTAGCCTTAGATGGAATTTACCAC
CCATTTGATCTGCAATCCCAAGCAAATCGACTCGTCGAAGACGCACCGTACGACGCGGTC
CGAGGCACAGACGGGATTGTCACCCTCTCCGATGCCCTGTTCCAAGGGACTTCTCCCCGG
ACCTTGCCTGGCGTCGCCTCTTTAGACTACAACCCAACCTTGTATTTAAGCGGCCAGTTT
ACAATCTGGGCTGATCCCGCTTCAGTCGCCCTTACTAGGGGAATCCTTGTTAGTTTCTTT
TCCTCCGCTTAGTTATATGCTTAAATTCAGCGGGTCCTCTTGCGTCACGGAGGCTCAAGA
TTGAGTGTTTATTACTAAACAGGCGTCCTCGATCCGAAGATCAAAGACTGGGCCTCTCCG
CCGTCTCATTGCTTACTCAAAAGCTCTTCGGTCCTCGGCTGGGTGAGTTAGGTGCTTTAC
GAGAAAGCCTAGTACCCCGCCTCGGACGGACCGAATGAGGCGGCGCGACGAGAGCTTGAC
TTGGCGCCACGATCGAGAACCAATTGGTCCATCGAAGCGCAGACTCCACCGCCACCGAGT
AGTAACACGCCGTGGGGTGATGTGGGGGACAAGCACTCCGTCACGCATGCTCCCAGGCAT
ATCCCGGGAGCGCAAGATGCGTTCAAAGGTTTGATGATTCACTGGATTCTGCAATTCGCA
TTACGTATCGCATTTCGCTGCGTTCTTCATCGTTGTGGGAGCCAAGACATCCGCCGGTAA
GAGTTGTATGGATTTGTATGGCATTTCGCTTGATCAAAATTAGTTGTTACACTAATCTTT
CGGCGTCAGCATGCGCTGGTTCACCCGTTTGGTTAGAATCGGAGTGTGAGTGACTGTGGA

CGATGACCCAATCAAGAAGTACACCGAAGTCAGAGGTACTAACTGAATAGTACCGGTTCG
CTGGGGTGTGTTTAGGTGTGGATATTATGGTAATGATCCCTCCGCAGGTTCACCTACGGA
GACCTTGTTACGACTTCACCTTCCTCTAAATAGTGAGGTTTAACCAACTTCCCGAGGTAC
GTTCGCCGTGAAGCTTGCGAACCCCAGTCCGGAGGTTTCACCGAACCATTCAATCGGTAG
GTGCGACGGGCGGTGTGTACAAAGGGCAGGGACGTAATCAATGCGAGCTGATGACTCGCG
TTTACTAGGAATTCCTCGTTCATGATTGAAAATTGCAACAATCAATCCCAATCACGGCAC
ACGTTCACAGATTACCCAGGCCTGCCGACCAAGGTGATAGACTCGTTGCGTGTGCCAGTG
TAGCGCGCGTGCGGCCCAGGACATCTAAGGGCATCACAGACCTGTTATTGCCCCCAGCTT
CCTTCGGTTAGTCACCGAAAGTCCCTCTAAGAAGTTGAGGCAATGCAAGCGCATTCCCAC
AGCTATTTAGCAGGCGGGGGTCTCGTTCGTTAACGGAATTAACCAGACAAATCACTCCAC
CAACTAAGAACGGCCATGCACCACCACCCATAGAATCAAGAAAGAGCTCTCAATCTGTCA
ATCCTTACTATGTCTGGACCTGGTAAGTTTCCCCGTGTTGAGTCAAATTAAGCCGCAGGC
TCCACTCCTGGTGGTGCCCTTCCGTCAATTTCTTTAAGTTTCAGCCTTGCGACCATACTC
CCCCCGGAACCCAAAGATTTTGATTTCTCATACAGTGCTGACGGAGTCGAGAACAAACGA
CCGCCAATCCTGAATCGGCATAGTTTATGGTTAAGACTACGATGGTATCTAATCATCTTC
GATCCCCTAACTTTCGTTCTTGATTAATGAGAACGTCCTTGTTCGACGCTTTCGCAGCAG
TTCGTCTTCCATAAATCCAAGAATTTCACCTCTGACAATTGAATACGAAGAACCCCAACC
GTCCCTTTTAATCATTACCTCGGCGTGCAAACCAACAAAATAGACCGCCAAGGTCCTATC
TCATTATTCCATGCTAATGTATTCAAGGCGTAAGCCTGCGTTGAACACTCTGATTTTTTC
ACAGTAAACGTCGGGAATCCCCACCCCGACAACTGAATGCCAGGACCGCCCTTCCCAAGG
ATGGCCGAGGCCGCTCAGTGCGCACCTTTTGCAAGGCGGACCGGCTACCCCGTGCCAGGA
ATCCAACTACGAGCTTTTTAACTGCAACAACATTAATATACGCTATTGGAGCTGGAATTA
CCGCGGCTGCTGGCACCAGACTTGCCCTCCAATGGATCCTCGATAAGGGATTTAAATTGT
TCTCATTCCAATTGCCAGACTCGTAAGAGCCCGGCATTGTTATTTCTTGTCACTACCTCC
CTGTGTCAGGATTGGGTAATTTACGCGCCTGCTGCCTTCCTTGGATGTGGTAGCCGTCTC
TCAGGCTCCCTCTCCGGAATCGAACCCCAATTCTCCGTTACCCGTTAATGCCATGGTAGG
CCAATACCCTACCATCCAAAGCTGATAGGGCAGAAACTTGAATGATCCATCGCCGGCACA
AGGCCATGCGATTCGCAAAGTTATCATGACTCACCACAATACCGGGGTCTCCCCCGCATT
GGTTTCAATCTAATAAATACGTCCCGTCCGCCGAAGCAGTTGGGAGTCGATGCATGTATT
AGCTCTAGAATTACTACGGGTATCCATGTAGTAAGGGACTATCAAATAAACTATAACTGA
TATAATGAGCCATTCGCAGTTTCACAGTACAAAATCGTTTATACTTAGACATGCATGGCT
TAATCTTTGAGACAAGCGCAATATCAGCACCAACAGAAAGGTTAACCTGGTAACTGGGAC
ACAAGACTCCAGCACCAGCAATACGTAACTTCGCCTGTCGCTCTATTTCCCTTGGTCCGT
GTTTCAAAACTTGCATGGCTTAATCTTTGAGACAAGCGCAATATCAACACCAACAGAAAG
GTTAATCCATTCCCTCCGATAGATGAAACCAGCACCAGCAATACGTAACTA

>Sequence_5_Dinophysis_consensus_cl_id_49_total_supporting_reads_6625
AAAATATATTGTACTTCGTTCAGTTACGTATTGCTGGTGCTGGTAAGTTGGGTATGCAAC
GCAATGTTAACCTTTCTGTTGGTGCTGATATTGCGCTTGTCTCAAAGATTAAGCCATGCA
TGTCTCAGTATAAGCTTCAACACGGCAAAACTGCGAATGGCTCATTAAAACAGTTACAAT
TTATTTGATTGTCATTCATTACATGGATAACTGTGGTAATTCTAGAGCTAATACATGCCC
AACAATCCGACTCCGTGGAAGGGTTGTATTTATTAGATACAGAACCAACCCAAGCTCTGC
TTGGTCTCGTGGTGATTCATAATAAACGAATGAATTGCATGGTACCAACTGGCGATGTAT
CATTCAAGTTTCTGACCTATCAGCTTCCGACGGTACGGTATTGGCCTACCGTGGCAATGA
CGGGTAACGGAGAATTAGGGTTCGATTCCGGAGAGGGAGCCTGAGAAACGGCTACCACAT
CTAAGGAAGGCAGCAGGCGCGCAAATTACCCAATCCTGACACAGGGAGGTAGTGACAAGA
AATAACAATACAGGGCATCCATGTCTTGTAATTGGAATGAGTAGAATTTAAACCCCTTTA
CAAGTATCAATTGGAGGGCAAGTCTGGTGCCAGCAGCCGCGGTAATTCCAGCTCCAATAG
CGTATATTAAAGTTGTTGCGGTTAAAAAGCTCGTAGTTGGATTTCTGCTGAGGACGACCG
GTCCGCCCTCTGGGTGTGTATCTGGCTCGGCCTGGGCATCTTCTTGGAGAACGTAGCTGC
ACTTGACTGTGTGGTGCGGTATCCAGGACTTTTACTTTGAGGAAATTAGAGTGTTTCAAG
CAGGCATATGCCTTGAATACATTAGCATGGAATAATAAGATAGGACCTTGGTTCTATTTT
GTTGGCTTCTAGAGCCGAGGTAATGATTAATAGGGATAGTTGGGGGCATTCGAATTTAAC
TGTCAGAGGTGAAATTCTTGGATTTGTTAAAGACGGACTACTGCGAAAGCATTTGCCAAG
GATGTTTTCATTGATCAAGAACGAAAGTTAGGGGATCGAAGACGATCAGATACCGTCCTA
GTCTTAACCATAAACTATGCCAACTAGAGATCGGAGGTCGTTATCTATACGACTCCTTCA
GCACCTTATGAGAAATCAAAGTCTTTGGGTTCCGGGGGGAGTATGGTCGCAAGGCTGAAA
CTTAAAGGAATTGACGGAAGGGCACCACCAGGAGTGGAGCCTGCGGCTTAATTTGACTCA
ACACGGGAAAACTTACCAGGTCCAGACATAGTAAGGATTGACAGATTGATAGCTCTTTCT
TGATTCTATGGGTGGTGGTGCATGGCCGTTCTTAGTTGGTGGAGTGATTTGTCTGGTTAA
TTCCGTTAACGAACGAGATCTTACCTTGCTAAATAGTTACGTGTAACTTCGTTTACGCGG
GCAGCTTCTTAGAGGGACTTTGTGTGTATAACGCAAGGAAGTTTGAGGCAATAACAGGTC
TGTGATGCCCTTAGATGTTCTGGGCTGCACGCGCGCTACACTGATGTGCTCAACGAGTTT
ATGACCTTGCCCGGAAGGGTTGGGTAATCTGTGTTAAATCACATCGTGATGGGGATCGAT
TATTGCAATTATTAATCTTCAACGAGGAATTCCTAGTAAGCGCGAGTCATCAGCTCGTGC
TGATTACGTCCCTGCCCTTTGTACACACCGCCCGTCGCTCCTACCGATTGAGTGTTCCGG
TGAATAATTCGGACTGCAGCAGTGTTCAGTTCCTGAGCGTTGCCGTGGAAAGTCTGGTGA
ACCTTAACACTTAGAGGAAGGAGAAGTCGTAACAAGGTTTCCGTAGGTGAACCTGCGGAA
GGATCATTCGCACGCATCCAATATCCATAACTTGAAATTTCCTGTGTGAGCTTCTGGGTG
AGGTTGAACAAAGTGTTGCCTTCATGTGGAAGCTCGAGGGTAGATGAACTGAAGCAGTGT
GGTCTTGCTGTTTCGTGGGCGCTACCGTCTGCTTGGCTCACACTGCCTTGCGGTTGAACC
TTTATTTGTTTGTATGTGCATGCTGTATGTATCACAATTTTCAGCGATGGATGCCTCGGC
TCGAACAACGATGAAGGGCGCAGCGAAGTGTGATAATCTTTGTGAATTGCAGAATTCCGT
GAATCAATAGAGCTTTGAACGTGTCTTGCGCTTTCGGGCTATACCTGAAAGCATGCCTGC
GTTGGTGTCTGTATGGCCTCATTCAGTCAGCAACACTAAACCTCATTGGTAGTGTGATGT
CTGTGTGTCGGTGTGTGCAAGGTTCACCCTTGTTACACAGAATGCATGTTTATTATCGTT
GCCAACAGCTTGCTGTGCCTCTGTATAGTGGTTAACCTTCTGTTGGCACGAAATCCAAGC
ACATGCCCCAATGTGTGATTAATTAGACATGACGTTAGGCTAGATCACCTGCTGAATTTA
AGCATATAAGTAAGCGGAGGACAAGAAACTAAACAGGATCCCCATAGTAATGGCGAATGA
ACCGGGATAAACTCAGCATGGAAATTGGGGCCTTTGGCTTTGACTTGTAATCTGGTGAGG
CATTGCCAACAGAGGCGCATATGTAAGTCTCTTGGAAAAGAGCATCAACGAAGGTGAGAG
TCCTGATTGTCATGTGCAGCCCCCCTGTGCACGGTGTGCCTTCTAAGAGTCACGTTCCTT
GGGATTGGAGCGCAAATTGGGTGGTAACTTTCATCTGAAGTTAAACAATGGTTTGGGACC
GATAGGAAACAAGTACCATGAGGGAAAGGTGAAAAGGACTTTGGAAAGAGAGTTAAAAGT
GCCTGAAATTGCTGAAGGGGAAGCGAATGGAACCAGTGGTGCTGGGTGAGATTGTCACAT

GCTGCAAGGATTGGTTGCTGCCACAACGCAAGTGTGCTGGCAATTTGTGAACTGCTAGTG
TGTGGTGTGTCTTACCTAGTGGGTCATTGTGGGTTTATGCATTAAGATAAATCAAAGCAA
GCGGGAGCAAGTTTACGAGTTTGTGAATGTGTGTTGTTATGCTTGTGCGTAAGCTTTAGT
GTTTGTCTGGCTGCAGGGCTGTCCATCCTCAAGCTTTGCTGTGGTTGCGATGAGCATAAC
CTTCTGCACAGATCTTGACGAAATGGTTTCATTCGACCCGTCTTGAAACACGGACCAAGG
GAAGATAGAGCGACAGGCAAGTAGGTTAACATTGCGTTGCATACCCAACTTACCAGCACC
AGCAATACGTAATGCATAA

>Sequence_6_Karlodinium_consensus_cl_id_0_total_supporting_reads_218
GATGTACTTCGTTCAGTTACGTATTGCTGGTGCTGGTTTCATCTATCGGAGGGAATGGAT
TAACCTACTTGCCTGTCGCTCTATCTTCCCTTGGTCCGTGTTTCAAGAGCATGGCTTAAT
CTTTGAGACAAGCATTGTACTTCGTTCAGTTACGTATTGCTGGTGCTGAAGCGTTGAAAC
CTTGTGTCCCGTTCTCAGGTTAACCTTTCTGTTGGTGCTGATATTGCGCTTGTCTCAAAG
ATTAAGCCATGCATGTCTCAGTATAAGCTTCTATACGGCGAAACTGCGAATGGCTCATTA
AAACAGTTATAGTTTATTTGATGGTCATTCATTACATGGATAACTGTGGTAATTCTAGAG
CTAATACATGCGCCCAAACCCGACTCCGTGGAAGGGTTGTGTTTATTAGACACAGAACCA
ACCCAGGCTCTGCCTGGTATTGTGGTGATTCATAGTAACCGAACGAATCGCATAGCTTCA
GCTGGCGATAAATCATTCAAGTTTCTGACCTATCAGCTTCCGACGGTAGGGTATTGGCCT
ACCGTGGCAATGACGGGTAACGGAGAATTAGGGTTCGATTCCGGAGAGGGAGCCTGAGAA
ACGGCTACCACATCTAAGGAAGGCAGCAGGCGCGCAAATTACCCAATCCTGACACAGGGA
GGTAGTGACAAGAAATAACAATACAGGGCATCCATGTCTTGTAATTGGAATGAGTAGAAT
TTAAATCCCTTTTCGAGTATCAATTGGAGGGCAAGTCTGGTGCCAGCAGCCGCGGTAATT
CCAGCTCCAATAGCGTATATTAAAGTTGTTGCGGTTAAAAAGCTCGTAGTTGGATTTCTG
CCGAGGACGACCGGTCCGCCCTCTGGGTGCGTATCTGGCTCGGCCTGGGCATCTTCTTGG
AGAACGTGTCTGCACTTGACTGTGTGGTGCGGTATCCAGGACTTTTACTTTGAGGAAATT
AGAGTGTTTCAAGCAGGCACACGCCTTGAATACATTAGCATGGAATAATAAGATAGGACC
TCGGTTCTATTTTGTTGGTTTCTAGAGCTGAGGTAATGATTAATAGGGATAGTTGGGGGC
ATTCGTATTTAACTGTCAGAGGTGAAATTCTTGGATTTGTTAAAGACGGACTACTGCGAA
AGCATTTGCCAAGGATGTTTTCATTGATCAAGAACGAAAGTTAGGGGATCGAAGACGATC
AGATACCGTCCTAGTCTTAACCATAAACCATGCCGACTAGAGATTGGAGGTCGTTATCCA
TACGACTCCTTCAGCACCTTATGAGAAATCAAAGTCTTTGGGTTCCGGGGGGAGTATGGT
CGCAAGGCTGAAACTTAAAGGAATTGACGGAAGGGCACCACCAGGAGTGGAGCCTGCGGC
TTAATTTGACTCAACACGGGGAAACTTACCAGGTCCAGACATAGTAAGGATTGACAGATT
GATAGCTCTTTCTTGATTCTATGGGTGGTGGTGCATGGCCGTTCTTAGTTGGTGGAGTGA
TTTGTCTGGTTAATTCCGTTAACGAACGAGACCTTAACCTGCTAAATAGTTACACGTAAC
TTCGGTTACGTGGGCAACTTCTTAGAGGGACTTTGCGTGTCTAACGCAAGGAAGTTTGAG
GCAATAACAGGTCTGTGATGCCCTTAGATGTTCTGGGCTGCACGCGCGCTACACTGATGC
GCTCAACGAGTTTATGACCTTGCCCGGAAGGGTTGGGTAATCTTTTTAAAACGCATCGTG
ATGGGGATAGATTATTGCAATTATTAATCTTCAACGAGGAATTCCTAGTAAGCGCGAGTC
ATCAGCTCGTGCTGATTACGTCCCTGCCCTTTGTACACACCGCCCGTCGCTCCTACCGAT
TGAGTGATCCGGTGAATAATTCGGACTGCAGCAGTGTTCAGACCCTGAACGTTGCAGCGG
AAAGTTTAGTGAACCTTATCACTTAGAGGAAGGAGAAGTCGTAACAAGGTTTCCGTAGGT
GAACCTGCGGAAGGATCATTCACACGCATCAACAATCTCACTGTGAACTTCCTTTGTGAG
CTCTTGCGCGGGGATAGGGGCAGTAGCCGCTTCCTGGTCATTAGTTCGCGGGTAGTGGAG
CAGAATCGGACCGTCTTCCTTTGCTGCGAAACTACCGTTATTCGTGCAACACGTGTGTTT
CTCATTCGTACTTTCGTGGTGGCTTTGTCCACTCTTTGCATTGAACACAACTTTCAGCGA
CGGATGTCTCGGCTCGAACAACGATGAAGGGCGCAGCGAAGTGTGATAAGCATTGTGAAT
TGCAGAATTCCGTGAACCAATAGGGATTTGAACGTATACTGCGCTTTCGGGACATCCCTG
AAAGCATGCCTGCTTCAGTGTCTACTGATATTCCTGCCTCTGACGCATGCCCAGTATTTG
CTTGGCATTGTCAGATGTGCGTGTTTGTGTGTCAAAGAGCTCTGTGCCTTTGACGCATTC
AGTGCATAGGTGCTGTCCACAACGAGCAACTTAATTAGCACTTTCAGTGTCTTGTGTTGT
TCTGTTGTTGGACACTTGCCTGCTCCACCTTGTCTTTGTGATATTATTCAAGACATGAAG
TTAGGTCAGCAAACCCGCTGAATTTAAGCATATAAGTAAGCGGAGGATAAGAAACTAAAT
AGGATTCCCTCAGTAATGGCGAATGAACAGGGATCAGCTCAGCATGGAAATTGGGGCCTC
CGGCCTTGAATTGTAGTCTTGAGATGGCATTGCCAGCGGAGGCGCAGATGTAAGCCTCTT
GGAAAAGAGCATCAATGAGGGTGAGAGTCCCGTTTGTCATCTGCAGTCCCCCGTTCACGG
TGTGTCTTCTAAGAGTCACGTTCCTTGGGATTGGAGCGCAAATTGGGTGGTAAATTTCAT
CTAAAGCTAAATATTGGTTCGAGACCGATAGCAAACAAGTACCATGAGGGAAAGGTGAAA
AGGACTTTGAAAAGAGAGTTAAAAGTGCCTGAAATTGCTGAAAGGGAAGCGAATGGAACC
AGTGTTTCTTGGCTAGATTGTTGCGTGCTAAAGTGATGGCTCGCTGCTTCAACGCAAGTG
TTGCAGTGGGTTTTGATCTGGACGTGCGCGGTGCTTCTGCCTTGTGTGTCAACGTCGGTT
CAGACTTGAGGAAAACTCCAGGGACATGGTAAATCGCTCCTGGGTGATTGAATGTGCCTG
GCAGAACTCATTTCTGAACTGATTCCTGCATGTCTGGTCGCAGTGCTTTCACTGCCTGTG
TCTCGTTCGGGGTGCCTTGTGTGCTCTGTGCTGGGCCGGGACGTGGAGTATCTCCCTGAC
ATAAGTACGATGACAAAATGGTTTTATTCGACCCGTCTTGAAACACGGACCAAGGGAAGA
TAGAGCGACAGGCAAGTAGGTTAACCTGGTAACTGGGACAAAGGTTTCAACGACTTCAGC
ACCAGCAATACGTAACTCG

>Sequence_7_Karenia_consensus_cl_id_19_total_supporting_reads_58
CGGTATGCTTCGTTCAGTTACGTATTGCTGGTGCTGCAGGTAGAAAGAAGCAGAATCGGA
TTAACCTTTCTGTTGGTGCTGATATTGCGCTTGTCTCAAAGATTAAGCCATGCATGTCTC
AGTATAAGCTTCTATACGGCGAAACTGCGAATGGCTCATTAAAACAGTTATAGTTTATTT
GATGGTCATTCATTACATGGATAACTTTGGAAATTCTAGAGCTAATACATGCGCCCAAAC
CCGACTTCTTGGAAGGGTTGTGTTTATTAGATACAGAACCAACCCAGGCTCTGCCTGGTC
TCGTGGTGATTCATAATAACCGAACGAATCGCATTGCATCAGCTGGCGATAGATCATTCA
AGTTTCTGACCTATCAGTTTCCGACGGTAGGGTATTGGCCTACCGTGGCGTTGACGGGTA
ACGGAGAATTAGGGGTTCGATTCCGGAGAGGGAGCCTGAGAAACGGCTACCACATCTAAG
GAAGGCAGCAGGCGCGCAAATTACCCAATCCTGACACAGGGAGGTAGTGACAAGAAATAA
CAATACAGGGCATCCATGTCTTGTAATTGGAATGAGTAGAATTTAAATCCCTTTGCGAGT
ATCAATTGGAGGGCAAGTCTGGTGCCAGCAGCCGCGGTAATTCCAGCTCCAATAGCGTAT
ATTAAAGTTGTTGCGGTTAAAAAGCTCGTAGTTGGATTTCTGCCGAGGACGACCGGTCCG
CCCTCTGGGTGAGTATCTGGCTCGGCTTGGGCATCTTCTTGGAGAACGTAGCTGCACTTG
ACTGTGTGGTGCGGTATCCAGGACTTTTACTTTGAGGAAATTAGAGTGTTTCAAGCAGGC
ATACGCCTTGAATACATTAGCATGGAATAATAAGATAGGACCTCGGTTCTATTTTGTTGG
TTTCTAGAGCTGAGGTAATGATTAATAGGGATAGTTGGGGGCATTCGTATTTAACTGTCA

GAGGTGAAATTCTTGGATTTGTTAAAGACGGACTACTGTGAAAGCATTTGCCAAGGATGT
TTTCATTGATCAAGAACGAAAGTTAGGGGATCGAAGACGATCAGATACCGTCCTAGTCTT
AACCATAAACCATGCCGACTAGAGATTGGAGGTCGTTACTTATACGACTCCTTCAGCACC
TTATGAGAAATCAAAGTCTTTGGGTTCCGGGGGAGTATGGTCGCAAGGCTGAAACTTAAA
GGAATTGACGGAAGGGCACCACCAGGAGTGGAGCCTGCGGCTTAATTTGACTCAACACGG
GGAAACTTACCAGGTCCAGACATAGTAAGGATTGACAGATTGATAGCTCTTTCTTGATTC
TATGGGTGGTGGTGCATGGCCGTTCTTAGTTGGTGGAGTGATTTGTCTGGTTAATTCCGT
TAACGAACGAGACCTTAACCTGCTAAATAGTTACACGTAACTTCGGTTACGTGGGCAACT
TCTTAGAGGGACTTTGCGTGTCTAACGCAAGGAAGTTTGAGGCAATAACAGGTCTGTGAT
GCCCTTAGATGTTCTGGGCTGCACGCGCGCTACACTGATGCGCTCAACGAGTTTTCGACC
TTGCCCGGAAGGGTTGGGTAATCTTTTTAAAACGCATCGTGATGGGGATAGATTATTGCA
ATTATTAATCTTCAACGAGGAATTCCTAGTAAGCGCGAGTCATCAGCTCGTGCTGATTAC
GTCCCTGCCCTTTGTACACACCGCCCGTCGCTCCTACCGATTGAGTGATCCGGTGAATAA
TTCGGACTGCCGCAGTGTTCAGTTCCTGAACGTTGCAGTGGAAAGTTTAGTGAACCTTAT
CACTTAGAGGAAGGAGAAGTCGTAACAAGGTTTCCGTAGGTGAACCTGCGGAAGGATCAT
TCACACGCATCCAACTTGAATTCATTGTGAACCATCTTCTGTGAGTTGTGCCCCTGGCTG
GAGACAACTTGTCTACAACCTTGGGCAGAATCTCGCGGGTAGTTCGGGTGATATTGGACC
GTCTTCCTCTATTGTCTGGCTACCATTGTTGTTTGACCAATGTTTTGCATGATGACCAAT
CTTCCTTAAGATGTGGGTGACCACATGTTTGATTCACATACAACTTTCAGCGACGGATGT
CTCGGCTCGAACAACGATGAAGGGCGCAGCAAAGTGTGATAAGCATTGTGAATTGCAGAA
TTCCGTGAACCAATAGGGATTTGAACGTATACTGCGCTTTCGGGATATCCCTGAAAGCAT
GCCTGCTTCAGTGTCAATATTCTCTCATGCCACTGTCATCTTGTCATGTGCATTGCGCAT
GCAACTGACAGCAGTGTGTGTCTGTGCATTAAGGTGCTCTTCCTGCCCCTGATGCATTGA
ATCCATGGGTTTCGTGCTCTGTCAGCAACTTGATAAACACCTTGTGCTTTGTGTGTAACC
TGTTGCTTTGTCTGTTGCACTGATCAACCTGTCCTCTTATCTGTCACTCATTTGTGGCCT
CTTTCCATGACATGAAGTTAGGTAAGCAAACCCGCTGAATTTAAGCATATAAGTAAGCGG
AGGATAAGAAACTAAATAGGATTCCCTCAGTAATGGCGAATGAACAGGGATAAGCTCAGC
ATGGAAATTGGGGCCCTCGGCCTTGAATTGTAGTCTTGAGATGTGTTACCAACGGAGGCG
CAGATGTAAGCCTCTTGGAAAAGAGCGTCAGGGAGGGTGAGAGTCCCGTATGTCATCTGC
AGTTCTCTGTGCACGGTGCATGTTCTAAGAGTCACGTTCCTCGGGATTGGAGCGCAAATT
GGGTGGTAAATTTCATCTAAAGCTAAATATTGGTTCGAGACCGATAGCAAACAAGTACCA
TGAGGGAAAGGTGAAAAGGACTTTGAAAAGAGAGTTAAAAGTGCCTGAAATTGCTGAAAG
GGAAGCGAATGGAACCAGTTGTTCTTGGTGAGTATTGGTGTTGTCTAAGGTGATGGCTTG
CCACTTCAACGCAAGTGTGGTGGCAGGTTTTGATCTGGATGCGATACTGCTTCTCGCCTT
GCATGTCAACGTCAGTTCATAATTGAGGAAAACTCTAGGGACATGGTAATTTGCTTCCGG
GTGACTGAATGTCCTCAGTTGAACTCATTTTTGAACTGCTCTCTGTGTGTCTGGTAGCAC
TGCTCCATGTGCTTGCCTGCGATCTTCTGCTTTGCATGAAGGTTGTTGGTGCCAGGAGCA
TGTCCTTGACATAAGAACGATGACGAAATGGTTTTATTCGACCCGTCTTGAAACACGGAC
CAAGGGAAGATAGAGCGACAGGCAAGTAGGTTAATCCGATTCTGCATCAATCGCAAGCCA
GCACCAGCAATACGTAATA

>Sequence_8_Noctiluca_consensus__cl_id_16_total_supporting_reads_1465
AAAATCTTGTACTTCGTTCAGTTACGTATTGCTGGTGCTGTCGATTCCGTTTGTAGTCGT
CTGTTTAACCTTTCTGTTGGTGCTGATATTGCGCTTGTCTCAAAGATTAAGCCATGCATG
TCTCAGTATCAACTTTTACATGGTAACACTGCGAATGGCTCATTAAAACAGTTATAGTTT
ATTTGATGGCATTCATTATATGGATACCTTTGGTAATTCTAGAGCTAATACATGCATACA
GACCCGACTTATGAAGGGTTGTGTTTATTAGATTCCAAACCTTCCAACTTCGGTTGTGTT
CTTGATGATTCATGATAAAATACTGATCGCTTTTGGCGACGAGTCTTTCAAGTTTCTGAC
CTATCAGCTTTCGATGGTAGGGTATTGGCCTACCATGGCAGTGACGGGTAACAGAGAATT
AGGGTTCGATTCTGGAGAGGGAGCCTGAGAAACGGCTACCACTTCTAAGGAAGGCAGCAG
GCGCGTAAATTACCCAATCCTGACACAGGGAGGTAGTGACAAGAAATAACAATACAGGGC
ATCCATGTCTTGTAATTGGAATGAGTAAAATTTAAATCTCTTTACGAGTATCAATTGGAG
GGCAAGTCTGGTGCCAGCAGCCGCGGTAATTCCAGCTCCAATAGCGTATATTAAAGTTGT
TGCGGTTAAAAAGCTCGTAGTTGGATTTCTGCCGAGGACAGTCGGTCCGCCCTTGCGGGT
GAGCATCTGGATGGCCCTGGGCATCTTTTCCGAAGACTGTATCTGCACTTCATTGTGTGG
AGCGGTATTCGGAACCTTTACTTTGAGGAAATTAGAGTGTTTCAGGCATGCGCTTGTGGT
GAATACATTAGCATGGAATAATAAGATAGGACTCGTGGCCTACTTTGTTGGTTTGTGTGC
CATAGGTAATGATTAATAGGGATAGTTGGGGGCATTCGTATTTAATTGTCAGAGGTGAAA
TTCTTGGATTTGTTAAAGACGAACTACTGCGAAAGCATTTGCCAAGGATGTTTTCATTGA
TCAAGAACGAAAGTTAGGGGATCGAAGACGATCAGATACCGTCGTAGTCTTAACCATAAA
CCATGCCGACTAGAGATTGGGGGTCGTTAGCTTTATGACTCCTTCAGCACCTTATGAGAA
ATCAAAGTTTTTGGGTTCTGGGGGGAGTATGGTCGCAAGGCTGAAACTTAAAGGAATTGA
CGGAAGGGCACCACCAGGAGTGGAGCCTGCGGCTTAATTTGACTCAACACGGGAAAACTC
ACCAGGTCCAGACATAGTAAGGATTGACAGATTGATAGCTCTTTCTTGATTCTATGGGTG
GTGGTGCATGGCCGTTCTTAGTTGGTGGAGTGATTTGTCTGGTTAATTCCGTTAACGAAC
GAGACCTTAGCCTGCTAAATAGTGTCAAGTATGATTCTTATTTGATTACTTCTTAGAGGG
ACTTTGTGTGTCTAACGCAAGGAAGTTTGAGGCAATAACAGGTCTGTGATGCCCTTAGAT
GTTCTGGGCTGCACGCGCGCTACACTGATGCATTCAACGAGTTTATAACCTTGCCTGAAA
AGGTTGGGTAATCTGCAATGTGCATCGTGATGGGGATAGATTATTGCAATTATTAATCTT
CAACGAGGAATTCCTAGTAAGCGCGAGTCATCAGCTCGCGCTGATTACGTCCCTGCCCTT
TGTACACACCGCCCGTCGCTCCTACCGATTGAGTGATTCGGTGAATAATTCGGAGATTGA
TTTGTTCAGCTTTGCAGAACATGTCTTTCGAAGTTTAGTGAACCTCATCACTTAGAGGAA
GGAGAAGTCGTAACAAGGTTTCCGTAGGTGAACCTGCGGAAGGATCATTCACACATTCCA
ATGTCATGTTTGTGCACGTAGCGTTGTGGCCATGTACATGACGTTCAGACGTCTACGTGC
ATGGCTAAGGGTGGGAGGTGGATTTGTTGATTACGGTCAGCAAAGTTCGCCTGCCACTGT
CTTGACAATAATTTTCACCATCTTAATATTTGCCACTTTCGTTGATGGCTTGCAGTTGTA
TGAACAACACAACTTCCAGTGATGGATGTCTCGGCTCGAACAACGATGAAGGGTGCAGCG
AAGTGTGATAAGCATTGTGAATTGCAGGATTCAGTGAACCAACAGAAAGTTGAACGCATA
TTGCTCTTTCGGGTTATTCCTGAAAGCATGTTTGCTTCAGTGTCTTTTGTTTGTTCTGCG
GGGCGTGTCACTCAGGTGTCACGTTTTCGTGTTTTCATGCTTCAATGCAGCATTCGTTGC
ATTGACACATGTGTCTTGGTGGTGCTGGACATGCGCAACGTTGAACATCTTCTTCAGGAG
GGATGTTACGTGTGATCAAGCCGCCCCTGTGTGTATTTTTTAACCCTAAGGCATGAAGTT
AAGCCAGAGAACCTGCTGAATTTAAGCATATAAGTAAGCGGAGGAAAAGAAACTAAATAG
TATTCCCTTAGTAATGGCGAATGAATTGGGATAGGCTCAGCATGGAAATCCAGGCTTTCC
AGCCTTGACTTGTGATCACATGATGCAATACCAGCGTGGGCGCAGATGCAAATTCCTTGG

AAAAGGTTACCAGCGCAGGTGAGCGTCCTGTCTGTCATTTGCAGTCCCGCGCGTACGGTA
TGCACTCTCAGAGTCACGTTCCTTGGGAGTGGAGCGCAAAGTGGGTGGTAAACTTCATCT
AAAGCTTAATATATGTTTGAGACCGATAGCGAACAAGTACTATGAAGGAAAGATGCAAAG
GACTTTGAAAAGAGAGTTAAAAGTGCCTGAAATTGCTGGAAGGGAAGTGAGTTGGACCAG
TTTTGCTTGGTGAGATTGGCGCATGGCGCTGGCATTCATGTCAGTGTCTGCGTTGTTGCT
TGCCTTGTCTGTCAGAACCAGTTCACTTGTGAATTAAACATTATGCGTAGGTCGCACTCA
CTATCAGTGTGTGTTCACGCATCTGGGGATTTACAAGTGGATTGCGTTCAAACGCCAAGT
TGTTGAAGTGATAGTTCATTGCTGTTGTTCGATTCTTGGCGTTCGTTTCTGACTACATGG
TCTCGTTCGACCCGTCTTGAAACACGGACCAAGGGAAGATAGAGCGACAGGCAAGTAGGT
TAAACAGACGACTACAAACGGAATCGACAGCACCAGCAATACGTAATACG

>Sequence_9a_Prorocentrum_consensus_cl_id_162_total_supporting_reads_24_P_lima
AATTACGTATTGCTGGTGCTGAGAACGACTTCCATACTCGTGTGATTAACCTTTCTGTTG
GTGCTGATATTGCGCTTGTCTCAAAGATTAAGCCATGCATGTCTCAGCATAAGCTTCCAT
CCGGCGAAACTGCGAATGGCTCATTAAAACAGTTACAATTTATTTGGTGGTTCACTGTTA
CATGGATACCCGTGGAAATGCTAGAGCTAATACATGCGCTCCTACCCGACTTAGCAGAAG
GGTTGTGGTTATTAGTTACAGAACTAGCCCAGGCTTGCCTGGTCATGCGGTGACTCATGA
TAATGGAATTAGTCGTATGGCGTCTGCTGACGATAAATCATTCAAGCTTCTGACCTATCA
GCTTCCGACGGTAGGGTATTGGCCTACCGCGGCTTTGACGGGTAACGGAGAATTAGGGTT
TGATTCCGGAGAGGGAGCCTGAGAAATAGCTACCACATCTAAGGAAGGCAGCAGGCGCGC
AAATTACCCAATCCTGACACAGGGAGGTAGTGACAAGAAATAACAATACAGGGCATCCAT
GTCTTGTAATTGGAATGAGTAGAACTTAAATCTCTTTATGAGTACCAATTGGAGGGCAAG
TCTGGTGCCAGCAGCCGCGGTAATTCCAGCTCCAATAGCATATATTAAAGTTGTTGCGGT
TAAAAAGCTCGTAGTCGGATTTCTGCCGAGGACGACCGGTCCGCCCTCTGGGTGAGCATC
TGGCTTGATCTGGGCATCTTCTTGGAGAGCGTAGCTGCACTTGACTGTGTGGTGCGGTAT
CCAGGACTTTTACTTTGAGGAAATTAGAGTGTCCTAAGCAGGCCCATGCCATATATAATA
GCATGGAATAATAGGGTAGGACCTACTCTCTATTTTGTTGGTTTCTAGAGCAGAGGTAAT
GGTCAATGGGGATAGTTGGGGGTATCCGTATTTGACTGTCAGAGGTGAAATTCTTGGATT
TGTCAAAGACGAACCAATGCGAAAGCATTTGCCAGAGATGTTTTCCTTGATCAAGAACGA
AAGTTAGGGGATCGAAGACGATCAGATACCGTCCTAGTCTTAACCATAAACTATGCCAAC
TAGAGATTGGAGGTCGTTATGTTGACGACTCTTTCGGCACCTTATGAGAAATCAAAGTCT
TTGGGTTCCGGGGGGAGTATGGTCGCAAGGCTGAAACTTAAAGGAATTGACGGAAGGGCA
CCACCAGGAGTGGGGCCTGCGGCTTAATTTGACTCAACACGGGGAAACTTACCAGGTCCG
GACATAGTAAGGATTGACAGATTGACAGCTCTTTCTTGATTCTATGGGTGGTGGTGCATG
GCCGTTCTTAGTTGGTGGAGTGATTTGTCTGGTTAATTCCGTTAACGAACGAGACCTTAA
CTTGCTGAATAGCTACTTCTAACCCCGGTTACATGGGCAGCTTCTTAGAGGGACTTTGCT
GTGTCTAACGCAAGGAAGTTTGAGGCAATAACAGGTCTGTGATGCCCTTAGATGTTCTGG
GCTGCACGCGCGCTACACTGATGCGCCCAATGAGTTTTTGACCTTGCCTGGTAAGGTTGG
GTAATCTGTCAAAAAACGCATCGTGATGGGGATAGATTATTGCAATTATTAATCTTGAAC
GAGGAATTCCTAGTAAGTATGAGTCATCAATTCGTGTTGATTACGTCCCTGCCCTTTGTA
CACACCGCCCGTCGCTCCTACCGATTGAGTGATCCGGTGAATAATTCAGACTCTGCAGCT
CTCAACTCGCGAGTGTTGCAATGGAAAGTTTAGTGAGCCTTATCACTTAGAGGAAGGAGA
AGTCGTAACAAGGTTTCCGTAGGTGAACCTGCGGAAGGATCATTCGTGTACATCAGGGAA
CACCTGTGTGTGAAAAAGCAATATCATGGAGTCTGGGTGGGGTGGGGATAGCTCTCCGCG
CAGATGTCCGAGGGTGGCTTGGAATGGCGCAACAAGCTCCAAACCTGCCACCGTTCCTCG
CTTTCTCTCTTATATTATTTGCTTGCGCCGGCTACAAGCGTTTCATCGTGTCTTGTGGTT
TGCCTTATTTACGCTCGCTTTCAGCGATGGATGTCTTGGCTCGAACAATGATGAAGGGCG
CAGCGAAGTGCGATGAGCATTGTGAGTTGCAGGATTCCGTGAGCCAACAAGGAATTGAAT
GCGCACAGCACTTTCGGGATAGCCTTGCAAGTGTGCCTGTGTTGGCGTCTACATTGATTC
AGCGCTGCACCCTTGCTAGCCTCAAGCTTGTCACTGGTCAGCACTTGTGCATCGGGGCAC
GATGTGGCCCAATGTGCTCATGTCTTGCTGTGGCCCTTGATAAGTGCCAATGCGCCACAC
CTTGTGTCAGTGCAGCATTTCGAGATGAAGCGCTCAGATCTTACCTGACACAATGTTAGG
CCAGTGAACCCGCTGAATTTAAGCATATTAGTAGGCGGTGGAAGATAATCCAAAAGGGAT
TCCTTCAGTAATGGCGAATGAACGGGGAAAAGCTCAGCAAAGAAATCGAGGACTCCGGCC
TTGAGTTGTGGACTTGAGAGGTATCGCCAATGGTGGCACAGGTGGGGGCCTCTTGGAAAA
GGGCACCACAGAGGGTGTGAGTCCTGTTTGCCATCTGTAGTCCGCTGTGTACGGCATGCC
TCCTCCGAGTCACGTTCCTTGGAATTGGAGCGCGAAGGTGGTGGTGGCTTTCATCTCAAG
CTCAAGTATGGGTTCGAGACCGATAGCAAACAAGTACCATGAGGGAAAATTGAAAAGAGC
TTTGGAAAGAGAGTTAAAAGTGCCCGAACTTGCTGATAGGGAAGCGAAGGGAGCTGACGT
TGCTTGGTAACATCTTCCGCGCAGGACTAGGATGGGTTCTGATTGCAATGTGTGTTGTGT
TTCTTGCCATGCGTGTCAATGCCAAGTAATGAGTGGGGAGAGCTCCAGGGATAATGATTG
TTTCTGTCCCCGGGTAGACGAATATGTCTGGGTGCATCCATTTGTGGGCTGAAAAATTGG
TGGTTGGTTGCAGCTCCCTGCCTACGAGGCAATTGGGGTGTAGCCCTGACACGCAGACGA
GGACAAAGTGCTTCTGTTCGACCCGTCTTGAAACACGGACCAAGGGAAGATAGAGCGACA
GGCAAGTAGGTTAATCACACGAGTATGGAAGTCGTTCTCAGCACCAGCAATACGTAACTG
AACGAAGTACAATGATT

>Sequence_9b_Prorocentrum_consensus_cl_id_4_total_supporting_reads_87_P_spinulentum
ATTCATATTGTACTTCGTTCGGTTACGTAATACTGGTGCTGATACTCAGGAAAGTACTCT
GATTTAACCTTTCTGTTGGTGCTGATATTGCGCTTGTCTCAAAGATTAAGCCATGCATGT
CTCAGTATAAGCTTCTATACGGCGAAACTGCGAATGGCTCATTAAAACAGTTATAGTTTA
TTTGATGGTCATTCTTTACATGGATAACTGTGCTAATTGTAGAGCTAATACATGCGCCCA
AACCCGACTTATAGGAAGGGTTGTGTTTATTAGTTACAGAACCAGCCCAGGCTCCGCCTG
GTCCTTTGGTGATTCATAATAACCGAACGAATCGCATAGCTTCGCTGGCGATGAATCATT
CAAGTTTCTGACCTATCAGCTTCCGACGGTAGGGTATTGGCCTACCGTGGCAATGACGGG
TAACGGAGAATTAGGGTTCGATTCCGGAGAGGGAGCCTGAGAAACGGCTACCACATCTAA
GGAAGGCAGCAGGCGCGCAAATTACCCAATCCTGACACAGGGAGGTAGTGACAAGAAATA
ACAATACAGGGCATATCTGTCTTGTAATTGGAATGAGTAAATTTAAATCCCTTTACGAGT
ACCAATTGGAGGGCAAGTCTGGTGCCAGCAGCCGCGGTAATTCCAGCTCCAATAGCGTAT
ATTAAAGTTGTTGCGGTTAAAAAGCTCGTAGTTGGATTTCTGCCGAGGACGACCGGTCCG
CCCTCTGGGTGAGTATCTGGTTCGGCCTGGGCATCTTCTTGGAGAACGTGTCTGCACTTG
ACTGTGTGGTGCGGTATCCAGGACTTTTACTTTGAGGAAATTAGAGTGTTTCAAGCAGGC
TTACGCCTTGAATACATTAGCATGGAATAATAAGATAGGACCTCGGTTCTATTTTGTTGG
TTTCTAGAGCTGAGGTAATGATTAATAGGGATAGTTGGGGGCATTCGTATTTAACTGTCA
GAGGTGAAATTCTTGGATTTGTTAAAGACGGACTACTGCGAAAGCATTTGCCAAGGATGT

TTTCATTGATCAAGAACGAAAGTTAGGGGATCGAAGACGATCAGATACCGTCCTAGTCTT
AACCATAAACCATGCCGACTAGAGATTGGAGGTCGTTATTTATACGACTCCTTCAGCACC
TTATGAGAAATCAAAGTCTTTGGGTTCGGGGGGAGTATGGTCGCAAGGCTGAAACTTAAA
GGAATTGACGGAAGGGCACCACCAGGAGTGGAGCCTGCGGCTTAATTTGACTCAACACGG
GGAAACTTACCAGGTCCAGACATAGTAAGGATTGACAGATTGATAGCTCTTTCTTGATTC
TATGGGTGGTGGTGCATGGCCGTTCTTAGTTGGTGGAGTGATTTGTCTGGTTAATTCCGT
TAACGAACGAGACCTTAACCTGCTAAATAGTTACACGTAACTTCGGTTACGTGGGCAACT
TCTTAGAGGGACTTTGCGTGTCTAACGCAAGGAAGTTTGAGGCAATAACAGGTCTGTGAT
GCCCTTAGATGTTCTGGGCTGCACGCGCGCTACACTGATGCGTTCAACGAGTTTATGACC
TTGCCTGGAAAGGTTGGGTAATCTTTTTAAATCGCATCGTGATGGGGATAGATTATTGCA
ATTATTAATCTTCAACGAGGAATTCCTAGTAAGCGCGAGTCATCAGCTCGTGCTGATTAC
GTCCCTGCCCTTTGTACACACCGCCCGTCGCTCCTACCGATTGAGTGATCCGGTGAATAA
TTCGGACTGCTGCGGTGTCCAGTTCCTGGACCTGCTTGCGGAAAGTTTAGTGAACCTTAT
CACTTAGAGGAAGGAGAAGTCGTAACAAGGTTTCCGTAGGTGAACCTGCGGAAGGATCAT
TCGCACGCATCCATTTGATTCATTGTGAACAACAGTTGGTGAGGATCTGGGTGGGGATGG
AGATAGCATCGATGCCCCCATGCGGATACTCGGGGGCAGCAAGCCAGGCTCAGACCGTCT
TCTGGGCTTGTCCTTGCTGCCAGTGTTAGTTTGATACTCCGTATCTTCCAATTTGTTCTG
AGTGGTCTTCCACTCTTTATCTTCTTACAACTTTCAGCGACGGATGTCTCGGCTCGAACA
ACGATGAAGGGCGCAGCGAAGTGTGATAAGCATTGTGAATTGCAGAATTCCGTGAACCAA
TAGGGACTTGAACGTATACTGCGCTTTCGGGATATCCCTGAAAGCATGCCTGCTTCAGTG
TCTATTCTTATTATTCCAGCATCTGACTTGTTCGGATTGCTTGTGTGTATTTGTGTGCCA
GGGCGCCCTCACAGGCCTCTGGCGCATTCAGTGCACTGGGTCTTCCCGCGCAAACAACTA
GAAGAGTATCTCTGATGCTATCTGTTGTCTTGTTGTCGGGCTGGGCCTCGTTGTCTAGTG
CATATCGCACTTATACCAAGACATGAAGTTAGGCCAGCAAACCCGCTGAATTTAAGCATA
TAAGTAAGCGGAGGATAAGAAACTAAATAGGATTCCCTTAGTAATGGCGAATGAACAGGG
ATCAGCTCAGCATGGAAATTGGGGCCCTCGGCCTTGAATTGTAGTCTCGAGATGTATCGC
CAACGGAGGCGCAGATGTAAGCCTCTTGGAAAGGAGCATCAACGAGGGTGAGAGTCCCGT
TTGTCATCTGCAGTCCCCCGTGCACGGCATACCTTCTAAGAGTCGCGTTCCTCGGAATTG
GAGCGTAAATTGGGTGGTAAATTTCATCTCAAGCTAAATATTGGTTCGAGACCGATAGCA
AACAAGTACCATGAGGGAAAGATGAAAAGGACTTTGAAAAGAGAGTTAAAAGTGCCTGAA
ATTGCTGAAAGGGAAGCGAATGGAACCAGTGTTGCTTGGCAGGATTGTCGGGTGCATGAC
GCTCCTGATCGTCTCCTGCCTTGTGTGTCAACGCCAGTTCGCGATCGAGGAAAACTCCAG
GGTCATGGTAGCTTGTCTTCGGGTGAGTGAATAGCCTTGGCAGAACTCATTTGCGGACTG
TTTCTTTCGTGTCTGGTTGCAGTGTCTTTGGCACCCTGGAGATCTCGGCTTGGCTTGCCA
CTGTCGGGTGACCCAGGTGACTTGGGCATTTCCCTGACGCAAAGACGATGACTAAATGGT
TCTATTCGACCCGTCTTGAAACACGGACCAAGGGAAGATAGAGCGACAGGCAAGTAGGTT
AAATCAGAGGGTACTTTCCAGAA

>Sequence_10b_Pseudo_nitzschia_consensus_cl_id_0_total_supporting_reads_273
TAAGTATTGTACTTCGTTCAGTTACGTATTGCTGGTGCTGGAGTCTTGTGTCCCAGTTAC
CAGGTTAACCTTTCTGTTGGTGCTGATATTGCGCTTGTCTCAAAGATTAAGCCATGCATG
TCTAAGTATAAATATTTTACTTTGAAACTGCGAACGGCTCATTATATCAGTTATAGTTTA
TTTGATAGTCCCTTACTACTTGGATACCCGTAGTAATTCTAGAGCTAATACATGCGTCAA
TACCCTTCTGGGGTAGTATTTATTAGATTGAAACCAACCCCTTCGGGGTGATGTGGTGAT
TCATAATAAGCTTGCGGATCGCATGCCTCTGGCGGCGATGGATCATTCAAGTTTCTGCCC
TATCAGCTTTGGATGGTAGGGTATTGGCCTACCATGGCTTTAACGGGTAACGGGAAATTA
GGGTTTGATTCCGGAGAGGGAGCCTGAGAGACGGCTACCACATCCAAGGAAGGCAGCAGG
CGCGTAAATTACCCAATCCTGACACAGGGAGGTAGTGACAATAAATAACAATGCCGGGCC
TTCTTAGGTCTGGCAATTGGAATGAGAACAATTTAAACCCCTTATCGAGTATCAATTGGA
GGGCAAGTCTGGTGCCAGCAGCCGCGGTAATTCCAGCTCCAATAGCGTATATTAAAGTTG
TTGCAGTTAAAAAGCTCGTAGTTGGATTTGTGGTGTGTCCAGTCGACCTTTGCTCTTTGA
GTGATTGTGTTGTACTGGTCTGCCATGTTTGGGTGGAATCTGTGTGGCATTAAGTTGTCG
TGCAGGGGATGCCCATCGTTTACTGTGAAAAAATTAGAGTGTTCAAAGCAGGCTTATGCC
GTTGAATATATTAGCATGGAATAATGATATAGGACCTTGGTACTATTTTGTTGGTTTGCG
CACTAAGGTAATGATTAAGAGGGACAGTTGGGGGTATTTGTATTCCATTGTCAGAGGTGA
AATTCTTGGATTTTTGGAAGACAAACTACTGCGAAAGCATTTACCAAGGATGTTTTCATT
AATCAAGAACGAAAGTTAGGGGATCGAAGATGATTAGATACCATCGTAGTCTTAACCATA
AACTATGCCGACAAAGGATTGGTGGAGTCTCGTTTCGTCTCCATCAGCACTTTGTGAGAA
ATCATAAGTCTTTGGGTTCCGGGGGAGTATGGTCGCAAGGCTGAAACTTAAAGAAATTGA
CGGAAGGGCACCACCAGGAGTGGAGCCTGCGGCTTAATTTGACTCAACACGGGAAAACTT
ACCAGGTCCAGACATAGTGAGGATTGACAGATTGAGAGCTCTTTCTTGATTCTATGGGTG
GTGGTGCATGGCCGTTCTTAGTTGGTGGAGTGATTTGTCTGGTTAATTCCGTTAACGAAC
GAGACCCCTGCCTGCTAAATAGCACGCATAGTGTTTATCACTGTGTAGTGCTTCTTAGAG
GACGTGCGTTCTATTAGACGCAGGAAGATAGGGGCAATAACAGGTCTGTGATGCCCTTAG
ATGTTCTGGGCCGCACGCGCGCTACACTGATGCATTCAACGAGTTCTACCTTGGCCGAGA
GGCCTGGGCAATCTTTTGAACCTGCATCGTGATAGGGATAGATTATTGCAATTATTAATC
TTGAACGAGGAATTCCTAGTAAACGCAGTTCATCAAACTGCATTGATTACGTCCCTGCCC
TTTGTACACACCGCCCGTCGCACCTACCGATTGAATGGTCCGGTGAAGCCTCGAGATTGT
GGTTAGTTTCCTTTATTGGAAGTTAACCACGAGAACTTGTCTAAACCTTATCATTTAGAG
GAAGGTGAAGTCGTAACAAGGTTTCCGTAGGTGAACCTGCGGAAGGATCATTACCACACC
GATCCAAGATCAGTCTTCATTGTGAATCTGATTTCAGAGGCAGCCTCGCTGTTCTCTGTC
GTCAACTTTAACCCTGGTGCTATCCAGCCCGTGCAATACCTTGTGTTTGTGTGGGTTGGT
TGGTGTCCACTTTACCCCCCATATTCTATATGGAAACTGGAAAGAACCAAATGACCTAAA
GCTAAAGTGCAGTTGGTCTGGTGTTGCGCCTTGTGCCAACACCGCCCTGTACACAAACCT
AATGACTTACAACTTTCAGCGGTGGATGTCTAGGTTCCCACAACGATGAAGAACGCAGCG
AAATGCGATACGTAATGCGAATTGCAAGACCTCGTGAATCATTAAGATTTTGAACGCACA
TTGCGCTTTCGGGATTTTCCCGGTAGCATGCTTGTCTGAGTGTCTGTGGATCCCACTCAG
CACTGGTCTATTCTGTAGGCTAGTCGCTGGTTGCTTTGGCCTTGACTGCCTCTGGCTGTC
TCTGCTTAAATTCTACAACACGTACGTGCATAGATCTAGAAACGTCTCAGCCCGTTCTAA
CTACGGCGTTGACATTCTCATCTATCTCTGGTAGCTTTTGTCTCTGGAGTTCAATTGAGT
TTGTTATCAGCTGTTGGAAGGACTACTGAGTCTGACACTAAGTCTAGCTTGCAGTTTTCT
GCTCGCTATCTAATTCCGGATCTCAGATCAAGCAAGATGACCCGCCGAATTTAAGCATAT
AATTAAGCGGAGGAAAAGAAACTAACTAGGATTCCCTCAGTAACGGCGAGTGAAGCGGGA
CTAGCTCAGGATGTGAATCTGCGCTTTTATGGCGCCGAATTGTGGTCTATAGACTTTGAC
GTTATCTGCCGGGCCAAGTTCCTTGGAAAAGGACAGCTGAGAGGGTGAGACTCCCGTCCG

CCTGGTAGAATGAGTCTTTGTCAACGAGTCGAGTTGTTTGGGATTGCAGCTCTAATTTGG
TGGTAAATTCCATCTAAAGCTAAATATTGGTGGGAGACCGATAGCGTACAAGTACCGTGG
GAAAGATGCAAAGAACTTTGAAAAGAGAGTTAAAGAGTACCTGAAATTGCTGAAACGGAA
GCGAAGGAAACCAGTGTTTGTTGGTTCATATTTCCCTGGCCACTTGTGGTTTGGGCGCTG
TGAGCTTGCGTGGGTTTGCTTTGGTTGATCCCTTTGGAAGAGCGCAGACAGAGTTGATGT
CTGTTGCTAGCACTGGGTTTGACTAATGCAGACGAAATGGTTTTCTTTACCCCGTCTTGA
AACACGGACCAAGGGAAGATAGAGCGACAGGCAAGTAGGTTAACCTGGTAACTGGGACAC
AAGACTCCAGCACCAGCAATACGTAACTTAA

>Sequence_10a_Pseudo-nitzschia_consensus_cl_id_2_total_supporting_reads_29_P_plurisecta
TTATTACGTATTGCTGGTGCTGAAGAAAGTTGTCGGTGTCTTTGTGTTAACCTTTCTGTT
GGTGCTGATATTGCGCTTGTCTCAAAGATTAAGCCATGCATGTCTAAGTATAAATATTTT
ACTTTGAAACTGCGAACGGCTCATTATATCAGTTATAGTTTATTTGATAGTCCCTTACTA
CTTGGATACCCGTAGTAATTCTAGAGCTAATACATGCGTCAATACCCTTCTGGGGTAGTA
TTTATTAGATTGAAACCAACCCTTCGGGGTGATGTGGTGATTCATAATAAGCTTGCGGAT
CGCATGCCTCTGGCGGCGATGGATCATTCAAGTTTCTGCCCTATCAGCTTTGGATGGTAG
GGTATTGGCCTACCATGGCTTTAACGGGTAACGGGAAATTAGGGTTTGATTCCGGAGAGG
GAGCCTGAGAGACGGCTACCACATCCAAGGAAGGCAGCAGGCGCGTAAATTACCCAATCC
TGACACAGGGAGGTAGTGACAATAAATAACAATGCCGGGCCTTCTTAGGTCTGGCAATTG
GAATGAGAACAATTTAAACCCCTTATCGAGTATCAATTGAGGGCAAGTCTGGTGCCAGCA
GCCGCGGTAATTCCAGCTCCAATAGCGTATATTAAAGTTGTTGCAGTTAAAAAGCTCGTA
GTTGGATTTGTGGTGTGTCCAGTTGGCCTTTGCTCTTTGAGTGATTGCGCTGTACTGGTC
TGCCATCTTTGGGTGGAATCTGTGTGGCATTAAGTTGTCGTGCAGGGGATGCCCATCGTT
TACTGTGAAAAAATTAGAGTGTTCAAAGCAGGCTTATGCCGTTGAATATATTAGCATGGA
ATAATAATATAGGACCTTGGTACTATTTTGTTGGTTTGCGCACTAAGGTAATGATTAAGA
GGGACAGTTGGGGGTATTTGTATTCCATTGTCAGAGGTGAAATTCTTGGATTTTTGGAAG
ACAAACTACTGCGAAAGCATTTACCAAGGATGTTTTCATTAATCAAGAACGAAAGTTAGG
GGATCGAAGATGATTAGATACCATCGTAGTCTTAACCATAAACTATGCCGACAAAGGATT
GGTGGAGTCTCGTTTCGTCTCCATCAGCACTTTGTGAGAAATCATAAGTCTTTGGGTTCC
GGGGGAGTATGGTCGCAAGGCTGAAACTTAAAGAAATTGACGGAAGGGCACCACCAGGAG
TGGAGCCTGCGGCTTAATTTGACTCAACACGGGAAAACTTACCAGGTCCAGACATAGTGA
GGATTGACAGATTGAGAGCTCTTTCTTGATTCTATGGGTGGTGGTGCATGGCCGTTCTTA
GTTGGTGGAGTGATTTGTCTGGTTAATTCCGTTAACGAACGAGACCCCTGCCTGCTAAAT
AGCTCGCATAGTGCTTGTCACTGTGTAGAGCTTTAGAGGGACGTGCGTTCTATTAGACGC
AGGAAGATAGGGGCAATAACAGGTCTGTGATGCCCTTAGATGTTCTGGGCCGCACGCGCG
CTACACTGATGCATTCAACGAGTTCTACCTTGGCCGAGAGGCCTGGGCAATCTTTTGAAC
CTGCATCGTGATAGGGATAGATTATTGCAATTATTAATCTTGAACGAGGAATTCCTAGTA
AACGCAGATCATCAATCTGCATTGATTACGTCCCTGCCCTTTGTACACACCGCCCGTCGC
ACCTACCGATTGAATGGTCCGGTGAAGCCTCGGGATTGTGGTTAGTTTCCTTTATTGGAA
GGTAACCACGAGAACCTGTCTAAACCTTATCATTTAGAGGAAGGTGAAGTCGTAACAAGG
TTTCCGTAGGTGAACCTGCGGAAGGATCATTACCACACCGATCCAAGATCTGTCTTCATT
GTGAATCTGATTGCGCGAGTACCATTAGCATTAGCTTTGCTGCTAGCGCCGTCAAACTTA
TATATTTCCTTGTGGTCCCGCCCGAGCTGGTTTTACTAGCGAAGGCCGCACTGCCTGGAT
CTTACTTTCAGGAGCGAGCACTGCTTAGCAACTTACACGCCCACCCCCATGTATATTATT
TTATATGGTAACTGGACCGAACCAAATGACCTAAAGCTTAAAGTGCAGTAGGAAGCATCG
TGCTACTTGTAGCCGGTGCGGCCCTGTACAAAAACCTAATGACTTACAACTTTCAGCGGT
GGATGTCTAGGTTCCCACAACGATGAAGAACGCAGCGAAATGCGATACGTAATGCGAATT
GCAAGACCTCGTGAATCATTAAGATTTTGAACGCACATTGCGCTTTCGGGATTTTCCCGG
TAGCATGCTTGTCTGAGTGTCTGTGGATCCCACTCAGCACTGGTGATTACTTTATTTTAT
TTTATCGTACTCACTAGTAGCTGGTTGCTTTGGCTTTGACGGTCTGAACTGACTGTCTTT
GCTTAAATTCTACATTCATGTACGTGCATAGATCTAGAAACGTCTTGGCCCGTTGAATAA
CGGTGCTGGCATTCTCGTCTATCTCTGGTAGCTTTAGGGCTTTCTTGAGTTTGATGGTGA
GATTGTTAAAGCCTGTCTTTTGGCAAGCCTTGATTGTGGCTAAATCTAGCTATGCTGTTA
ATGTTAAATTAACCGGCTAGCTATCCAATTCCGGATCTCAGATCAAGCAAGATGACCCGC
CGAATTTAAGCATATAATTAAGCGGAGGAAAAGAAACTAACTAGGATTCCCTCAGTAACG
GCGAGTGAAGCGGACTAGCTCAGGATGTGAATCTGCGCTTTAATGGCGCCGAATTGTGGT
CTATAGACCTTGACGTTATCTGCCGGGCCAAGTTCCTTGGAAAAGGACAGCTGAGAGGGT
GAGACTCCCGTCCGCCTGGTAGAGTGAGTCATGTGTCAACGAGTCGAGTTGTTTGGGATT
GCAGCTCTAATTTGGTGGTAAATTCCATCTAAAGCTAAATATTGGTGGGAGACCGATAGC
GTACAAGTACCGTGAGGGAAAGATGCAAAGAACTTTGAAAAGAGAGTTAAAGAGTACCTG
AAATTGCTGAAACGGAAGCGAAGGAAACCAGTGTTTGTTGGTTCATATTTCCCTGGCCAC
TTGTGGTTTGGGCGCTGTGAGCTTGCGTGTGTTTGTCTTGGTTGATCCCTTTGGAAGAGC
GCAGTCAGAGTTGATGTCTGTTGCTAGCACTGGGTTTGACTGATGCAGACGAAATGGTTT
TCTTTACCCCGTCTTGAAACACGGACCAAGGGAAGATAGAGCGACAGGCAAGTAGGTTAA
CACAAAGACACCGACAACTTTCTTCAGCACCAGCAATACGTAACTGAACGAAGTACAAGT
GATTGGG

>Sequence_10b_Pseudo-nitzschia_consensus_cl_id_1_total_supporting_reads_104_P_delicatissima
TTTTCTTTTCTTTAAGTTACGTATTGCTGGTGCTGAACTAGGCACAGCGAGTCTTGGTTT
TAACCTTTCTGTTGGTGCTGATATTGCGCTTGTCTCAAAGATTAAGCCATGCATGTCTAA
GTATAAATATTTTACTTTGAAACTGCGAACGGCTCATTATATCAGTTATAGTTTATTTGA
TAGTCCCTTACTACTTGGATACCCGTAGTAATTCTAGAGCTAATACATGCGTCAATACCC
TTCTGGGGTAGTATTTATTAGATTGAAACCAATCCCTTCGGGGTGATGTGGTGATTCATA
ATAAGCTTGCGGATCGCATGCCTCTGGCGGCGATGGATCATTCAAGTTTCTGCCCTATCA
GCTTTGGATGGTAGGGTATTGGCCTACCATGGCTTTAACGGGTAACGGGAAATTAGGGTT
TGATTCCGGAGAGGGAGCCTGAGAGACGGCTACCACATCCAAGGAAGGCAGCAGGCGCGT
AAATTACCCAATCCTGACACAGGGAGGTAGTGACAATAAATAACAATGCCGGGCCTTCTT
AGGTCTGGCAATTGGAATGAGAACAATTTAAACCCCTTATCGAGTATCAATTGGAGGGCA
AGTCTGGTGCCAGCAGCCGCGGTAATTCCAGCTCCAATAGCGTATATTAAAGTTGTTGCA
GTTAAAAAGCTCGTAGTTGGATTTGTGGTGTGTCCAGTCGACCTTTGCTCTTTGAGTGAT
TGTGTTGTACTGGTCTGCCATGTTTGGGTGGAATCTGTGTGGCATTAAGTTGTCGTGCAG
GGGATGCCCATCGTTTACTGTGAAAAAATTAGAGTGTTCAAAGCAGGCTTATGCCGTTGA
ATATATTAGCATGGAATAATGATATAGGACCTTGGTACTATTTTGTTGGTTTGCGCACTA
AGGTAATGATTAAGAGGGACAGTTGGGGGTATTTGTATTCCATTGTCAGAGGTGAAATTC
TTGGATTTTTGAAGACAAACTACTGCGAAAGCATTTACCAAGGATGTTTTCATTAATCAA

GAACGAAAGTTAGGGGATCGAAGATGATTAGATACCATCGTAGTCTTAACCATAAACTAT
GCCGACAAAGGATTGGTGGAGTCTCGTTTCGTCTCCATCAGCACTTTGTGAGAAATCATA
AGTCTTTGGGTTCCGGGGGAGTATGGTCGCAAGGCTGAAACTTAAAGAAATTGACGGAAG
GGCACCACCAGGAGTGGAGCCTGCGGCTTAATTTGACTCAACACGGGAAAACTTACCAGG
TCCAGACATAGTGAGGATTGACAGATTGAGAGCTCTTTCTTGATTCTATGGGTGGTGGTG
CATGGCCGTTCTTAGTTGGTGGAGTGATTTGTCTGGTTAATTCCGTTAACGAACGAGACC
CCTGCCTGCTAAATAGCACGCATAGTGTTTATCACTGTGTAGTGCTTTAGAGGGACGTGC
GTTCTATTAGACGCAGGAAGATAGGGGCAATAACAGGTCTGTGATGCCCTTAGATGTTCT
GGGCCGCACGCGCGCTACACTGATGCATTCAACGAGTTCTACCTTGGCCGAGAGGCCTGG
GCAATCTTTTGAACCTGCATCGTGATAGGGATAGATTATTGCAATTATTAATCTTGAACG
AGGAATTCCTAGTAAACGCAGTTCATCAAACTGCATTGATTACGTCCCTGCCCTTTGTAC
ACACCGCCCGTCGCACCTACCGATTGAATGGTCCGGTGAAGCCTCGAGATTGTGGTTAGT
TTCCTTTATTGGAAGTTAACCACGAGAACTTGTCTAAACCTTATCATTTAGAGGAAGGTG
AAGTCGTAACAAGGTTTCCGTAGGTGAACCTGCGGAAGGATCATTACCACACCGATCCAA
GATCAGTCTTCATTGTGAATCTGATTTCAGAGGCAGCCTCGCTGTTCTCTGTCGTCAACT
TTAACCCTGGTGCTATCCAGCCCGTGCAATACCTTGTGTTTGTGTGGGTTGGTTGGTGTC
CACTTTACCCCCATATTCTATATGGAAACTGGAAAGAACCAAATGACCTAAAGCTAAAGT
GCAGTTGGTCTGGTGTTGCGCCTTGTGCCAACACCGCCCTGTACACAAACCTAATGACTT
ACAACTTTCAGCGGTGGATGTCTAGGTTCCCACAACGATGAAGAACGCAGCGAAATGCGA
TACGTAATGCGAATTGCAAGACCTCGTGAATCATTAAGATTTTGAACGCACATTGCGCTT
TCGGGATTTTCCCGGTAGCATGCTTGTCTGAGTGTCTGTGGATCCCACTCAGCACTGGTC
TATTCTGTAGGCTAGTCGCTGGTTGCTTTGGCCTTGACTGCCTCTGGCTGTCTCTGCTTA
AATTCTACAACACGTACGTGCATAGATCTAGAAACGTCTCAGCCCGTTCTAACTACGGCG
TTGACATTCTCATCTATCTCTGGTAGCTTTTGTCTCTGGAGTTCAATTGAGTTTGTTATC
AGCTGTTGGAAGGACTACTGAGTCTGACACTAAGTCTAGCTTGCAGTTTTCTGCTCGCTA
TCTAATTCCGGATCTCAGATCAAGCAAGATGACCCGCCGAATTTAAGCATATAATTAAGC
GGAGGAAAAGAAACTAACTAGGATTCCCTCAGTAACGGCGAGTGAAGCGGGACTAGCTCA
GGATGTGAATCTGCGCTTTTATGGCGCCGAATTGTGGTCTATAGACTTTGACGTTATCTG
CCGGGCCAAGTTCCTTGGAAAAGGACAGCTGAGAGGGTGAGACTCCCGTCCGCCTGGTAG
AATGAGTCTTTGTCAACGAGTCGAGTTGTTTGGGATTGCAGCTCTAATTTGGTGGTAAAT
TCCATCTAAAGCTAAATATTGGTGGGAGACCGATAGCGTACAAGTACCGTGAGGGAAAGA
TGCAAAGAACTTTGAAAAGAGAGTTAAAGAGTACCTGAAATTGCTGAAACGGAAGCGAAG
GAAACCAGTGTTTGTTGGTTCATATTTCCCTGGCCACTTGTGGTTTGGGCGCTGTGAGCT
TGCGTGGGTTTGCTTTGGTTGATCCCTTTGGAAGAGCGCAGACAGAGTTGATGTCTGTTG
CTAGCACTGGGTTTGACTAATGCAGACGAAATGGTTTTCTTTACCCCGTCTTGAAACACG
GACCAAGGGAAGATAGAGCGACAGGCAAGTAGGTTAAAACCAAGACTCGCTGTGCCTAGT
TCAGCACCAGCAATACGTAACTGAACGAAGTACAATTT

>Sequence_10c_Pseudo-nitzschia_consensus_cl_id_2_total_supporting_reads_19_P_fraudulenta
ACAAATCAATGTGCTTCGTTCAGTTACGTATTGCTGGTGCTGACTGGTGCAGCTTTGAAC
ATCTAGTTAACCTTTCTGTTGGTGCTGATATTGCGCTTGTCTCAAAGATTAAGCCATGCA
TGTCTAAGTATAAATTTTTTACTTTGAAACTGCGAACGGCTCATTATATCAGTTATAGTT
TATTTGATAGTCCCTTACTACTTGGATACCCGTAGTAATTCTAGAGCTAATACATGCGTC
AATACCCTTCTGGGGTAGTATTTATTAGATTGAAACCAACCCCTTCGGGGTGATGTGGTG
ATTCATAATAAGCTTGCGGATCGCATGCCTCTGGCGGCGATGGATCATTCAAGTTTCTGC
CCTATCAGCTTTGGATGGTAGGGTATTGGCCTACCATGGCTTTAACGGGTAACGGGAAAT
TAGGGTTTGATTCCGGAGAGGGAGCCTGAGAGACGGCTACCACATCCAAGGAAGGCAGCA
GGCGCGTAAATTACCCAATCCTGACACAGGGAGGTAGTGACAATAAATAACAATGCCGGG
CCTTCTTAGGTCTGGCAATTGGAATGAGAACAATTTAAACCCCTTATCAGTATCAATTGG
AGGGCAAGTCTGGTGCCAGCAGCCGCGGTAATTCCAGCTCCAATAGCGTATATTAAAGTT
GTTGCAGTTAAAAAGCTCGTAGTTGGATTTGTGGTGTGTCTAGTCGGCCTTTGCTCTTTG
AGTGATTGTGCTGTACTGGTCTGCCATGTTTGGGTGGAATCTGTGTGGCATTAGGTTGTC
GTGCAGGGGATGCCCATCGTTTACTGTGAAAAAATTAGAGTGTTCAAAGCAGGCTTATGC
CGTTGAATATATTAGCATGGAATAATGATATAGGACCTTGGTACTATTTTGTTGGTTTGC
GCACTAAGGTAATGATTAAGAGGGACAGTTGGGGGTATTTGTATTCCATTGTCAGAGGTG
AAATTCTTGGATTTTGGAAGACAAACTACTGCGAAAGCATTTACCAAGGATGTTTTCATT
AATCAAGAACGAAAGTTAGGGGATCGAAGATGATTAGATACCATCGTAGTCTTAACCATA
AACTATGCCGACAAGGGATTGGTGGAGTTTCGTTTCGTCTCCATCAGCACCTTGTGAGAA
ATCATAAGTCTTTGGGTTCCGGGGGAGTATGGTCGCAAGGCTGAAACTTAAAGAAATTGA
CGGAAGGGCACCACCAGGAGTGGAGCCTGCGGCTTAATTTGACTCAACACGGGAAAACTT
ACCAGGTCCAGACATAGTGAGGATTGACAGATTGAGAGCTCTTTCTTGATTCTATGGGTG
GTGGTGCATGGCCGTTCTTAGTTGGTGGAGTGATTTGTCTGGTTAATTCCGTTAACGAAC
GAGACCCCTGCCTGCTAAATAGCACGCATAGTGTTTATCACTGTGTAGTGCTTCTTAGAG
GGACGTGCGTTCTATTAGACGCAGGAAGATAGGGGCAATAACAGGTCTGTGATGCCCTTA
GATGTTCTGGGCCGCACGCGCGCTACACTGATGCATTCAACGAGTTCTACCTTGGCCGAG
AGGCCTGGGCAATCTTTTGAACTTGCATCGTGATAGGGATAGATTATTGCAATTATTAAT
CTTGAACGAGGAATTCCTAGTAAACGCAGATCATCAATCTGCATTGATTACGTCCTGCCC
TTTGTACACACCGCCCGTCGCACCTACCGATTGAATGGTCCGGTGAAGCCTCGGGATTGT
GATTAGTTTCCTTTATTGGAAGGTAGTTATGAGAACCTGTCTAAACCTTATCATTTAGAG
GAAGGTGAAGTCGTAACAAGGTTTCCGTAGGTGAACCTGCGGAAGGATCATTACCACACC
GATCAAGATCAGTCTTCATTGTGAATTTGATTTGCGTGGCACTCTGGCTTCGGCCCGTTT
GCCTCAAAAGTCAACTTGTATACAGGTGGCCTTGTCCCCCCTGCGCACAGTATACCTGTG
TTCGGCGCGCGACATCGCACCAGTGGCATTAAGTGAAATGTCCACACCCCATCTTTTACG
ATCGGTAACTTGATAGAACCAAATGACCTAAAGCTTAAAGTGCAGTGGTCCGGCAACTGA
GCCTTGTGCCAGGAGCCGCCCTGTACATAAAACCTAATGACTTACAACTTTCAGCGGTGG
ATGTCTAGGTTCCCACAACGATGAAGAACGCAGCGAAATGCGATACGTAATGCGAATTGC
AAGACCTCGTGAATCATTAAGATTTTGAACGCACATTGCGCTTTCGGGATTTTCCCGGTA
GCATGCTTGTCTGAGTGTCTGTGGATCCCACTCAGCGCTGGTCTACTCTGTAGGCCCAGT
TGCTGGTTGCTATGGCTCTGACCGCTCTAGTAGCGGTCTCTGCTTAAATTCTACAACATT
GTACGTGCATAGATCTAGAAAAGTCTTGTGTTGTCTGAATGACGGCGTTGACGCTCTTGT
CTATCTCTGGTAGCTTGTATGAAAATGCATTCTCTGGAGTTTAACTAGGGGTGTCTATTA
GCTGTCTGCCAAACTATCGAGTTTTTCTCTGGGTCTGGCCACGCAGCTGTGTAAAAGCAG
TCTGCTGGCTATCCAATTCCGGATCTCAGATCAAGCAAGAGGACCCGCCGAATTTAAGCA
TATAATTAAGCGGAGGAAAAGAAACTAACTAGGATTCCCTCAGTAACGGCGAGTGAAGCG
GGACTAGCTCAGGATGTGAATCTGCGCTTTTATGGCGCCGAATTGTGGTCTGTAGACTTT

GACGTTATCTGCCGGGCCAAGTTCCTTGGAAAAGGACAGCTGAGAGGGTGAAACTCCCGT
CCGCCTGGTAGAATGAGTCTTTGTCAACGAGTCGAGTTGTTTGGGATTGCAGCTCTAATT
TGGTGGTAAATTCCATCTAAAGCTAAATATTGGTGGGAGACCGATAGCGTACAAGTACCG
TGAGGGAAAGATGCAAAGAACTTTGAAAAGAGAGTTAAAGAGTACCTGAAATTGCTGAAA
CGGAAGCGAAGGAAACCAGTGTTTGTTGGTTCATATTTCCCTGGCCACTTGTGGTTTGGG
CGCTGTGAGCTTGCGTGGGTTTGGGTTGGTTGATCCCTTTGGAAGAGCGCAGTCAGAGTT
GATGTCTGTTGCTAGCACTGGGTTTGACTGATGCAGACGAAATGGTTTTCTTTACCCCGT
CTTGAAACACGGACCAAGGGAAGATAGAGCGACAGGCAAGTAGGTTAACTAGATGTTCAA
AGCTGCACCAGTCAGCACCAGCAATACGTAATAA

>Sequence_10d_Pseudo-nitzschia_consensus_cl_id_6_total_supporting_reads_124_P_australis
ACAAAATCATATGTACTTCGTTCAGTTACGTATTGCTGGTGCTGAACGAGTCTCTTGGGA
CCCATAGATTAACCTTTCTGTTGGTGCTGATATTGCGCTTGTCTCAAAGATTAAGCCATG
CATGTCTAAGTATAAATATTTTACTTTGAAACTGCGAACGGCTCATTATATCAGTTATAG
TTTATTTGATAGTCCCTTACTACTTGGATACCCGTAGTAATTCTAGAGCTAATACATGCG
TCAATTCCCTTCTGGGGAAGTATTTATTAGATTGAAACCAACCCCTTCGGGGTGATGTGG
TGATTCATAATAAGCTTGCGGATCGCATGCCTCTGGCGGCGATGGATCATTCAAGTTTCT
GCCCTATCAGCTTTGGATGGTAGGGTATTGGCCTACCATGGCTTTAACGGGTAACGGGAA
ATTAGGGTTTGATTCCGGAGAGGGAGCCTGAGAGACGGCTACCACATCCAAGGAAGGCAG
CAGGCGCGTAAATTACCCAATCCTGACACAGGGAGGTAGTGACAATAAATAACAATGCCG
GGCCTTCTTAGGTCTGGCAATTGGAATGAGAACAATTTAAACCCCTTATCGAGTATCAAT
TGGAGGGCAAGTCTGGTGCCAGCAGCCGCGGTAATTCCAGCTCCAATAGCGTATATTAAA
GTTGTTGCAGTTAAAAAGCTCGTAGTTGGATTTGTGGTGTGTCCAGTCGGCCTTTGCTCT
TTGAGTGATTGCGCTGTACTGGTCTGCCATGTTTGGGTGGAATCTGTGTGGCATTAAGTT
GTCGTGCAGGGGATGCCCATCGTTTACTGTGAAAAAATTAGAGTGTTCAAAGCAGGCTTA
TGCCGTTGAATATATTAGCATGGAATAATGATATAGGACCTTGGTACTATTTTGTTGGTT
TGCGCACTAAGGTAATGATTAAGAGGGACAGTTGGGGGTATTTGTATTCCATTGTCAGAG
GTGAAATTCTTGGATTTTGGAAGACAAACTACTGCGAAAGCATTTACCAAGGATGTTTTC
ATTAATCAAGAACGAAAGTTAGGGGATCGAAGATGATTAGATACCATCGTAGTCTTAACC
ATAAACTATGCCGACAAGGGATTGGCGGAACGACGTTACGTCTCCGTCAGCACCTTGTGA
GAAATCATAAGTCTTTGGGTTCCGGGGGAGTATGGTCGCAAGGCTGAAACTTAAAGAAAT
TGACGGAAGGGCACCACCAGGAGTGGAGCCTGCGGCTTAATTTGACTCAACACGGGAAAA
CTTACCAGGTCCAGACATAGTGAGGATTGACAGATTGAGAGCTCTTTCTTGATTCTATGG
GTGGTGGTGCATGGCCGTTCTTAGTTGGTGGAGTGATTTGTCTGGTTAATTCCGTTAACG
AACGAGACCCCTGCCTGCTAAATAGCACGCATAATGTTTATCATTGTGTAGTGCTTCTTA
GAGGACGTGCGTTCTATTAGACGCAGGAAGATAGGGGCAATAACAGGTCTGTGATGCCCT
TAGATGTTCTGGGCCGCACGCGCGCTACACTGATGCATTCAACGAGTTCTACCTTGGCCG
AGAGGCCTGGGCAATCTTTTGAACTTGCATCGTGATAGGGATAGATTATTGCAATTATTA
ATCTTGAACGAGGAATTCCTAGTAAACGCAGATCATCAATCTGCATTGATTACGTCCCTG
CCCTTTGTACACACCGCCCGTCGCACCTACCGATTGAATGGTCCGGTGAAGCCTCGGGAT
TGTGGTTAGTTTCCTTTATTGGAAGGTAGTCACGAGAACCTGTCTAAACCTTATCATTTA
GAGGAAGGTGAAGTCGTAACAAGGTTTCCGTAGGTGAACCTGCGGAAGGATCATTACCAC
ACCGATCCAAGATCAGTCTTCATTGTGAATCTGATTTCAGAGGCGGCTTGCTTTTAAAGC
TCTCCGTCTCTGTCGTCAAACTTTTTTAGTGATGCGCTAGCTACTAGCGTATTACAACCC
CCATTCTTAACGATTGGTAACTGGAAAGAACCAAATGACCTAAAGCTAAAATGCAGTGGT
CTGGTGTTGCGCCTCGTGCCAACACCGCCCTGTACATTATACCAATTCATTACAACTTTC
AGCGGTGGATGTCTAGGTTCCCACAACGATGAAGAACGCAGCGAAATGCGATACGTAATG
CGAATTGCAAGACCTCGTGAATCATTAAGATTTTGAACGCACATTGCGCTTTCGGGATTT
TCCCGGTAGCATGCTTGTCTGAGTGTCTGTGGATCCCACTCAGCGCTGGTTTAGGCCAGT
CGCTGGTTGTTTTGGCCTTGACAGCTACTAGTAGCTGTCTCTGCTTAAGTTCTACGGTAT
CGTACGTGCATAGATTTAGAGACCTCAACCTGTCTTGTCAAGACGGCGTTGACATTCTTG
TCTATCTCTGGTAGCTTGTATTCATTACAAATATCTGGAGTTTGTTAAGATTGTCATGTT
CAGCTGTTTGAAACTTTTCGATTGAGCGTCACTAGATCTAGCTAGCTATACTTTCTAGTA
CTAGCTAGCTATCCAATTCCGGATCTCAGATCAAGCAAGAGGACCCGCCGAATTTAAGCA
TATAATTAAGCGGAGGAAAAGAAACTAACTAGGATTCCCTCAGTAACGGCGAGTGAAGCG
GGACTAGCTCAGGATGTGAATCTGCGCTTTTATGGCGCCGAATTGTGGTCTGTAGACTTT
GACATTATCTGCCGGGCCAAGTTCCTTGGAAAAGGACAGCTGAGAGGGTGAGACTCCCGT
CCGCCTGGTGGAGTGAGTCATTTGTCAACGAGTCGAGTTGTTTGGGATTGCAGCTCTAAT
TTGGTGGTAAATTCCATCTAAAGCTAAATATTGGTGGGAGACCGATAGCGTACAAGTACC
GTGAGGGAAAGATGCAAAGAACTTTGAAAAGAGAGTTAAAGAGTACCTGAAACTGCTGAA
ACGGAAGCGAAGGAAACCAGTGTTTGTTGGTTCATATTTCCCCGGCCACTTGTGGTTTGG
GCGCTGTGAGCTTGCGTGAGTTTGGGTTGGTTGAATCCTTTGGAAGAGCGCAGTCAGAGT
TGATGTCTGTTGCTAGCACTGGATTTGACTGATGCAGACGAAATGGTTTTCTTTACCCCG
TCTTGAAACACGGACCAAGGGAAGATAGAGCGACAGGCAAGTAGGTTAATCTATGGGTCC
CAAGAGACTCGTTCAGCACCAGCAATACGTAATAA
