# Supplementary material for: ScillyHAB: A Multi-Disciplinary Survey of Harmful Marine Phytoplankton and Shellfish Toxins in the Isles of Scilly: Combining Citizen Science with State-of-the-Art Monitoring in an Isolated UK Island Territory
Source: Mar Drugs. 2025 Dec 15;23(12):478. doi: 10.3390/md23120478 (PMC12734806; doi:10.3390/md23120478)
Supplement: Supplementary file 1 [file marinedrugs-23-00478-s001.zip › marinedrugs-4017030-supplementary/ScillyHAB supplementary tables v1.pdf]

**Table S1**: Summary of sampling sites utilised during study

| Site no | Sample type | Island | Place | Lat East | Long North |
|---|---|---|---|---|---|
| 1 | Water | Bryher | Great Par | 49.949 | -6.358 |
| 2 | Water | Bryher | Great Porth | 49.951 | -6.358 |
| 3 | Water | Bryher | Green Bay | 49.952 | -6.350 |
| 4 | Shellfish | Bryher | Green Bay Quay offshore | 49.951 | -6.348 |
| 5 | Both | Bryher | Hangman's Point | 49.958 | -6.351 |
| 6 | Water | Bryher | Hell Bay | 49.962 | -6.358 |
| 7 | Water | Bryher | Mussel Rock | 49.963 | -6.360 |
| 8 | Water | Bryher | Popplestone Bay | 49.953 | -6.360 |
| 9 | Water | Bryher | Stony Porth | 49.945 | -6.356 |
| 10 | Water | St Mary's | Airport beach | 49.911 | -6.297 |
| 11 | Water | St Mary's | Bar Point | 49.937 | -6.299 |
| 12 | Both | St Mary's | Deep Point | 49.922 | -6.276 |
| 13 | Water | St Mary's | Little Porth | 49.923 | -6.309 |
| 14 | Water | St Mary's | Main Harbour - Quay | 49.917 | -6.317 |
| 15 | Both | St Mary's | Old Town | 49.911 | -6.302 |
| 16 | Both | St Mary's | Old Town east | 49.911 | -6.301 |
| 17 | Both | St Mary's | Old Town west | 49.910 | -6.303 |
| 18 | Water | St Mary's | Pelistry Bay | 49.929 | -6.280 |
| 19 | Both | St Mary's | Porth Hellick | 49.915 | -6.283 |
| 20 | Water | St Mary's | Porth Mellon | 49.917 | -6.309 |
| 21 | Water | St Mary's | Porthloe | 49.931 | -6.288 |
| 22 | Water | St Mary's | Watermill cove | 49.931 | -6.288 |
| 23 | Both | St. Agnes | Bar beach | 49.894 | -6.336 |
| 24 | Water | St. Agnes | Bellend Beach | 49.886 | -6.340 |
| 25 | Water | St. Agnes | Covean Bay | 49.891 | -6.338 |
| 26 | Water | St. Agnes | Harbour | 49.894 | -6.337 |
| 27 | Water | St. Agnes | Harbour quay pool | 49.895 | -6.338 |

| | | | | | |
|---|---|---|---|---|---|
| 28 | Water | St. Agnes | Peraskin Cove | 49.885 | -6.338 |
| 29 | Water | St. Agnes | Periglis cove | 49.894 | -6.345 |
| 30 | Water | St. Agnes | Porth Coose | 49.896 | -6.349 |
| 31 | Water | St. Agnes | Porth Warna cove | 49.889 | -6.340 |
| 32 | Water | St. Agnes | Santa Warna | 49.889 | -6.347 |
| 33 | Water | Gugh | Bar - southside | 49.894 | -6.335 |
| 34 | Water | Gugh | Dropnose Porth | 49.894 | -6.336 |
| 35 | Both | Tresco | 1/2 way Tresco/Bryher | 49.953 | -6.345 |
| 36 | Water | Tresco | Appletree Bay South | 49.948 | -6.323 |
| 37 | Water | Tresco | Crab's Ledge | 49.944 | -6.324 |
| 38 | Water | Tresco | Crown Well | 49.962 | -6.348 |
| 39 | Shellfish | Tresco | English Island Point | 49.960 | -6.270 |
| 40 | Water | Tresco | Gimble Porth | 49.963 | -6.339 |
| 41 | Water | Tresco | Long Point Quay | 49.961 | -6.333 |
| 42 | Water | Tresco | Pentle Bay | 49.949 | -6.319 |
| 43 | Both | Tresco | Ravens Porth | 49.961 | -6.332 |
| 44 | Water | St. Martins* | English Island Point | 49.958 | -6.271 |
| 45 | Water | St. Martins* | Pigs Ledge | 49.957 | -6.288 |
| 46 | Water | St. Martins* | Porth Moran | 49.974 | -6.291 |
| 47 | Water | St. Martins* | Porth Seal | 49.970 | -6.298 |
| 48 | Shellfish | St. Martins* | St. Martins Flats A | 49.958 | -6.288 |
| 49 | Shellfish | St. Martins* | St. Martins Flats B | 49.960 | -6.294 |
| 50 | Water | St. Martins* | The Cove | 49.974 | -6.292 |

*Sampled only once during first March visit

**Table S2**. Summary of HABs detected and quantified (cell densities in cells/L) by laboratory light microscopy across all sampling sites from the Isles of Scilly, March to October 2020

| Sample | Island | Site Name | Sample | *Vulcanodinium* sp. | *Alexandrium* sp. | *Amphidinium cartarae* | *Dinophysis* sp. | *Dinophysis acuminata* | *Gymnodinium* | *Heterocapsa minima/Azadinium/Amphidoma* group | Indet. dinoflagellate | *Karenia* sp. | *Prorocentrum cordatum/balticum* | *Prorocentrum lima* | Prymnesiophytes | *Pseudo-Nitzschia delicatissima* group (≤4.9µm) | *Pseudo-nitzschia multistriata* | *Pseudo-Nitzschia Seriata* group (≥5µm) |
|---|---|---|---|---|---|---|---|---|---|---|---|---|---|---|---|---|---|---|
| 2 | St Mary's | Deep Point | 7/3/20 | - | - | - | - | - | - | - | - | - | - | - | - | - | - | - |
| 11 | St. Agnes | High Town Bay | 8/3/20 | - | - | - | - | - | - | - | - | - | - | - | - | - | - | - |
| 24 | Tresco | Ravens Porth | 9/3/20 | - | - | - | - | - | - | - | - | - | - | - | - | - | - | - |
| 36 | Bryher | Green Bay | 13/3/20 | - | - | - | - | - | - | - | - | - | - | - | - | - | - | - |
| 62 | St Mary's | Deep Point | 19/4/20 | - | - | - | - | - | - | - | - | - | - | - | - | - | - | - |
| 97 | Tresco | Gimble Porth | 26/4/20 | - | - | - | 100 | 100 | - | - | - | - | - | 300 | - | - | - | - |
| 101 | Tresco | Gimble Porth | 10/5/20 | - | 100 | - | - | - | - | - | - | - | - | 100 | - | - | - | - |
| 112 | Bryher | Green Bay | 11/5/20 | - | 200 | - | - | 100 | - | - | - | - | - | - | - | - | - | - |
| 69 | St Mary's | Deep Point | 12/5/20 | - | - | - | - | - | - | - | - | - | - | - | - | - | - | - |
| 126 | St Agnes | Perasliin | 19/5/20 | - | - | - | 100 | 100 | - | - | - | - | - | - | - | - | - | - |
| 72 | St Mary's | Deep Point | 31/5/20 | - | - | - | - | 200 | - | - | - | - | - | - | - | - | - | - |
| 104 | Tresco | Apple Tree South | 9/6/20 | - | - | - | 200 | 400 | - | - | - | - | - | - | - | - | - | - |
| 76 | St Mary's | Deep Point | 10/6/20 | - | - | - | - | - | - | - | - | - | - | - | - | - | - | - |
| 105 | Tresco | Crab Ledge | 20/6/20 | - | - | - | - | - | - | - | - | - | - | 100 | - | 4800 | 1200 | 1200 |
| 78 | St Mary's | Porth Hellick | 21/6/20 | - | - | - | - | 100 | - | - | - | - | - | 100 | - | - | - | - |
| 106 | Tresco | Gimble Porth | 21/6/20 | - | 100 | - | - | - | - | - | - | 100 | - | - | - | - | - | - |
| 80 | St Mary's | Porth Mellon | 22/6/20 | - | - | - | - | 100 | - | - | - | 100 | - | - | - | - | - | - |

| | | | | | | | | | | | | | | | | | | |
|---|---|---|---|---|---|---|---|---|---|---|---|---|---|---|---|---|---|---|
| 84 | St Mary's | Porth Hellick | 22/6/20 | - | 100 | - | 100 | 600 | - | - | - | - | - | - | - | - | - | - |
| 143 | St Agnes | Covean | 1/7/20 | - | - | - | - | - | - | - | - | - | 800 | - | - | - | - | 1600 |
| 132 | St Agnes | Santa Warna | 5/7/20 | - | - | - | - | - | - | - | - | - | - | - | - | 400 | - | - |
| 85 | St Mary's | Deep Point | 10/7/20 | 1800 | - | - | - | - | - | - | - | - | - | - | - | 1200 | 200 | 200 |
| 134 | St Agnes | Santa Warna | 10/7/20 | - | - | - | - | - | - | - | 100 | - | - | - | - | - | - | - |
| 131 | St Agnes | Covean | 11/7/20 | - | - | - | - | 1600 | - | - | - | - | 200 | - | - | - | - | - |
| 86 | St Mary's | Pelistry | 14/7/20 | - | - | 100 | - | 200 | - | 21000 | - | - | 400 | - | - | - | 400 | 400 |
| 87 | St Mary's | Little Porth | 14/7/20 | - | 100 | - | - | 500 | - | - | - | - | - | - | - | - | - | - |
| 88 | St Mary's | Porth Mellon | 14/7/20 | - | 200 | - | - | 1700 | - | - | - | 200 | - | - | - | 1800 | 200 | 200 |
| 90 | St Mary's | Porth Hellick | 26/7/20 | - | 100 | - | - | 400 | - | - | - | - | - | - | - | - | - | - |
| 108 | Tresco | Long Point Quay | 29/7/20 | - | 100 | - | - | - | - | - | - | - | - | - | - | - | - | - |
| 109 | Tresco | Gimble Porth | 29/7/20 | - | - | - | - | 100 | 100 | - | - | - | - | - | - | - | - | - |
| 91 | St Mary's | Deep Point | 9/8/20 | - | - | - | - | - | - | 48600 | - | - | - | - | - | - | - | - |
| 92 | St Mary's | Porth Hellick | 9/8/20 | - | 400 | - | 400 | 800 | - | - | - | - | - | 200 | - | - | - | - |
| 93 | St Mary's | Deep Point | 18/8/20 | - | - | - | - | 200 | - | - | - | - | - | - | - | - | - | - |
| 94 | St Mary's | Porth Hellick | 18/8/20 | - | - | - | 100 | 400 | - | - | - | 400 | 400 | 2200 | 100 | 800 | 600 | 2000 |
| 95 | St Mary's | Deep Point | 7/9/20 | - | - | - | - | - | - | 7201000 | - | - | - | - | - | - | - | - |
| 96 | St Mary's | Porth Hellick | 7/9/20 | 400 | - | - | - | 100 | - | - | - | - | - | 100 | - | - | - | - |
| 114 | Bryher | Stony Porth | 8/9/20 | - | - | - | - | - | - | - | - | - | 600 | - | - | - | - | 400 |
| 115 | Bryher | Great Pal | 8/9/20 | - | - | - | - | - | - | - | - | - | - | - | - | - | - | - |
| 228 | St Mary's | Porth Hellick | 2/10/20 | - | - | - | - | 200 | - | - | - | - | - | - | - | - | - | - |
| 229 | Tresco | Long Point Quay | 3/10/20 | - | - | - | - | - | - | - | - | - | - | - | - | - | - | - |
| 230 | Tresco | Gimble Porth | 3/10/20 | - | - | - | - | - | - | - | - | - | - | - | - | - | - | - |
| 231 | St Mary's | Deep Point | 5/10/20 | - | - | - | - | - | - | - | - | - | - | - | - | - | - | - |
| 232 | Bryher | Stony Porth | 9/10/20 | - | - | - | - | - | - | - | - | - | - | - | - | - | - | - |
| 233 | Bryher | Great Pal | 9/10/20 | - | - | - | - | - | - | - | - | - | - | - | - | - | - | - |
| 234 | St Agnes | Santa Warna | 14/10/20 | | | | | | | | | | | | | | | |
| 235 | St Agnes | Covean | 16/10/20 | | | | | | | | | | | | | | | |

**Table S3**: List of MRM transitions for targeted analysis of each method highlighting primary (1°) and secondary transitions (2°) and associated collision energies (CE) and cone voltages (CV)

| Method | Toxin | Pos 1° | Pos 2° | CE | CV | Neg 1° | Neg 2° | CE | CV |
|---|---|---|---|---|---|---|---|---|---|
| LT | OA, DTX2 | - | - | - | - | 803.5>255.1 | 803.5>113 | 48;55 | 70 |
| LT | DTX1 | - | - | - | - | 817.5>255.1 | 817.5>113 | 45;60 | 70 |
| LT | YTX | - | - | - | - | 570.5>467.4 | 570.5>396.2 | 30 | 38 |
| LT | homo YTX | - | - | - | - | 577.5>474.2 | 577.5>403.2 | 30 | 38 |
| LT | 45 OH YTX | - | - | - | - | 578.5>467.4 | 578.5>396.2 | 30 | 38 |
| LT | 45 OH homo YTX | - | - | - | - | 585.5>474.2 | 585.5>403.2 | 30 | 38 |
| LT | AZA1 | 842.5>654.4 | 842.5>362.3 | 50 | 42 | - | - | - | - |
| LT | AZA2 | 856.6>654.4 | 856.6>362.3 | 50 | 42 | - | - | - | - |
| LT | AZA3 | 828.5>658.4 | 828.5>362.3 | 50 | 42 | - | - | - | - |
| LT | PTX1/PTX11 | 892.5>821.5 | 892.5>213.1 | 25;37 | 32 | - | - | - | - |
| LT | PTX2 | 876.6>823.5 | 876.6>213.1 | 25;38 | 32 | - | - | - | - |
| LT | SPX1 | 692.5>164.1 | 692.5>444.3 | 45;35 | 42 | - | - | - | - |
| LT | GYM | 508.4>136.1 | 508.4>162.1 | 38 | 36 | - | - | - | - |
| Emerging LT | PnTx E | 784.2 > 164.0 | 784.2 > 446.2 | 55;45 | 40 | - | - | - | - |
| Emerging LT | PnTx F | 766.4 > 164.0 | 766.4 > 488.2 | 55;45 | 40 | - | - | - | - |
| Emerging LT | PnTx G | 694.5 > 164.0 | 694.5 > 458.1 | 55;50 | 40 | - | - | - | - |
| Emerging LT | BTX B2 | 1034.5 > 929.0 | 1034.5 > 947.0 | 40 | 40 | - | - | - | - |
| Emerging LT | BTX B4 | 1272.7 > 929.4 | 1272.7 > 326.2 | 35 | 80 | - | - | - | - |
| Emerging LT | BTX B5 | 911.5 > 875.5 | 911.5 > 839.0 | 20 | 60 | - | - | - | - |
| Emerging LT | PbTx 2 | 895.5 > 319.2 | 895.5 > 877.5 | 30 | 60 | - | - | - | - |
| Emerging LT | PbTx 3 | 897.5 > 725.5 | 897.5 > 129.0 | 30 | 60 | - | - | - | - |
| Emerging LT | S desoxy BTX B2 | 1018.6 > 248.2 | 1018.6 > 204.1 | 40 | 40 | - | - | - | - |
| Emerging LT | 13,19 didesmethyl spirolide C | 678.5 > 164.1 | 678.5 > 430.1 | 50;35 | 40 | - | - | - | - |
| Emerging LT | 20-methyl spirolide C | 706.5 > 163.6 | 706.5 > 346.2 | 50;35 | 40 | - | - | - | - |
| Emerging LT | 12methyl GYM | 522.7 > 135.0 | 522.7 > 120.5 | 40 | 40 | - | - | - | |
| Emerging LT | PbTX 1 | 867.2 > 221.0 | 867.2 > 611.0 | 30 | 60 | - | - | - | |

| | | | | | | | | |
|---|---|---|---|---|---|---|---|---|
| Emerging LT | Pinnatoxin A | 712.5 > 458.3 | 712.5 > 164.1 | 40 | 40 | - | - | - |
| Emerging LT | Pinnatoxin D | 782.0 > 164.0 | - | 55 | 40 | - | - | - |
| Emerging LT | Pinnatoxin H | 708.0 > 164.0 | - | 55 | 40 | - | - | - |
| PST | STX | 300.1>204.1 | 300.1>138.0 | 31, 24 | - | - | - | - |
| PST | NEO | 316.1>126.1 | 316.1>298.1 | 30, 20 | - | - | - | - |
| PST | dcSTX | 257.1>126.1 | 257.1>222.0 | 20, 20 | - | - | - | - |
| PST | dcNEO | 273.1>126.1 | 273.1>225.1 | 24, 18 | - | - | - | - |
| PST | doSTX | 241.1>60.0 | 241.1>206.1 | 25, 20 | - | - | - | - |
| PST | GTX2 | - | | | | 394.1>351.1 | 394.1>333.1 | 20, 18 |
| PST | GTX3 | 396.1>298.1 | - | 18 | | - | 394.1>333.1 | 22 |
| PST | GTX1 | - | - | | | 410.1>367.1 | 410.1> 349.1 | 15, 20 |
| PST | GTX4 | 412.1>314.1 | - | 18 | | - | 410.1>367.1 | 15 |
| PST | GTX5 | 380.1> 300.1 | - | 15 | | - | 378.1>122.0 | 22 |
| PST | GTX6 | 396.1> 316.1 | - | 12 | | - | 394.1>122.0 | 24 |
| PST | dcGTX2 | - | - | | | 351.1>164.0 | 351.1>333.1 | 22, 12 |
| PST | dcGTX3 | 353.1>255.1 | - | 15 | | - | 351.1>333.1 | 18 |
| PST | dcGTX1 | - | - | | | 367.1>274.1 | 367.1>349.1 | 20, 17 |
| PST | dcGTX4 | 369.1>271.1 | - | 20 | | - | 367.1>349.1 | 16 |
| PST | C1 | - | - | | | 474.1>122.0 | 474.1>351.1 | 38, 30 |
| PST | C2 | 396.1>298.1 | - | 15 | | - | 474.1>122.0 | 38 |
| PST | C3 | 412.1>332.1 | - | 12 | | - | 490.1>410.1 | 16 |
| PST | C4 | 412.1>314.1 | - | 14 | | - | 490.1>392.1 | 20 |
| TTXs | TTX | 320.1>302.1, 162.1 | - | 28, 44 | | - | - | - |
